# De Novo Assembly and Comparative Analysis of the Complete Mitochondrial Genome of *Mesenchytraeus* (Annelida, Enchytraeidae)

**DOI:** 10.1002/ece3.74293

**Published:** 2026-09-04

**Authors:** Juanjuan Chen, Horacio Naveira, Junqian Zhang, Naicheng Wu, Zhicai Xie

**Affiliations:** ^1^ The Key Laboratory of Aquatic Biodiversity and Conservation, Institute of Hydrobiology Chinese Academy of Sciences Wuhan China; ^2^ School of Geography and Remote Sensing, Zhejiang‐Germany Joint Laboratory on Remote Sensing of Coastal Ecosystem Ningbo University Ningbo China; ^3^ Grupo de Investigación en Bioloxía Evolutiva, Departamento de Bioloxía, Facultade de Ciencias, CICA Universidade da Coruña A Coruña Spain

**Keywords:** codon usage patterns, *Mesenchytraeus*, mitochondrial genome, nucleotide composition biases, phylogenetic analysis

## Abstract

The Changbai Mountain range is one of the key glacial refugia in Northeast Asia. *Mesenchytraeus* exhibits high species diversity, strong endemism, and widespread cryptic species in this region, for which mitogenomes provide useful molecular markers for exploring cryptic species complexes. This makes *Mesenchytraeus* an ideal model for studying mitogenome evolution among closely related lineages; however, no mitogenome data have been reported for this genus to date. In this study, we performed *de novo* assembly, annotation, and comparative analysis of the mitogenomes of 13 *Mesenchytraeus* species (14 individuals) from Changbai Mountain. All mitogenomes are typical circular molecules containing 37 genes, but putative control regions are rearranged and consistently located between *ATP6* and *trnR*. All species exhibit annelid‐specific strand nucleotide biases, characterized by negative GC skew and near‐zero AT skew. Codon usage analysis reveals that codon families with wobble U are significantly biased toward mtDNA codons, whereas those with wobble C or G are biased toward non‐mtDNA codons, suggesting a conserved mitochondrial codon usage pattern in annelids. All tRNAs form typical cloverleaf secondary structures except *trnS2*, which lacks the D‐stem and the dihydrouridine (DHU) arm in some species. The putative control regions commonly contain complex palindromic repeats, hairpins, and repetitive elements, and may harbor dual replication origins. Phylogenetic analyses support the monophyly of *Mesenchytraeus* and reveal significant molecular divergence among morphologically cryptic species. This study provides the first mitogenome dataset for *Mesenchytraeus* and offers new insights into the evolution and replication mechanisms of mitogenomes in Clitellata and broader Annelida.

## Graphical abstract

This present the first mitochondrial genome dataset for 13 Mesenchytraeus species from the Changbai Mountain glacial refugium. Beyond characterizing standard genomic features, we perform comparative analyses of gene order, nucleotide composition, codon usage, and control region structure.

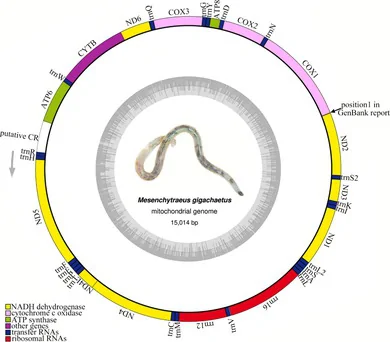

## 1 Introduction

The Changbai mountain range is considered to have served as one of the most important glacial refugia for cool‐temperate deciduous forests and their associated biota in northern East Asia (Bai et al. 2010; Qiu et al. 2011; Zeng et al. 2015). Now, it is home to a large number of highly divergent species and genetic lineages in many types of organisms, which experienced prolonged isolation and persisted throughout multiple glacial cycles (Zou et al. 2016; Zhang, Ganin, et al. 2020; Yu et al. 2021; Pan et al. 2025; Tian et al. 2025). This could also be the case for the rich soil enchytraeid fauna of this region, with *Mesenchytraeus* showing high species diversity and local endemism, accounting for ca. 20% of the known global record (Lian et al. 2011; Jiang et al. 2019; Chen et al. 2024). Moreover, the presence of morphologically cryptic species within *Mesenchytraeus* from this region provides an ideal system for investigating mitogenome evolution among closely related lineages.

The mitogenomes of metazoans are typically compact circular molecules, containing 13 protein‐coding genes (PCGs), 22 transfer RNA genes (tRNAs), 2 ribosomal RNA genes (rRNAs), and a non‐coding control region (CR) (Clary and Wolstenholme 1984; Shadel and Clayton 1997; Boore 1999). Due to the long‐term functional interactions and coevolution between mitochondria and host since the origin of endosymbiosis, mitogenomes are not only involved in energy metabolism but also closely associated with a wide range of traits and adaptive processes (Boore 1999; Hill et al. 2019). Strong internal selection between mitochondrial and nuclear genomes across different lineages has led to many different stable evolutionary configurations, shaped by lineage‐specific mitochondrial DNA constraints at different taxonomic levels, dating back to distant events of eukaryotic lineage splitting (Wolff et al. 2014; Zardoya 2020; Estes et al. 2023). These evolutionary constraints leave certain footprints in terms of gene order, nucleotide composition biases, codon usage patterns, and secondary structures, which are fundamental to our understanding of the origin and maintenance of mitochondrial DNA diversity.

These footprints are reflected at multiple levels of mitochondrial structure and composition. At the level of genome organization, annelid mitogenomes generally possess relatively conserved gene order, yet gene rearrangements and control region repositioning also occur across different lineages (Boore and Brown 1998; Dowton et al. 2002; Zhang et al. 2016; Struck et al. 2023). Because such structural changes are relatively rare, they are considered to contain important phylogenetic information (Struck et al. 2023; Oceguera‐Figueroa et al. 2016; Weigert et al. 2016). Meanwhile, animal mitogenomes commonly exhibit pronounced strand‐specific nucleotide composition biases, which typically arise from asymmetric replication mechanisms of mtDNA (Reyes et al. 1998; Formaggioni et al. 2021). During replication, the two DNA strands are exposed to single‐stranded states for different durations, leading to differential exposure to hydrolytic and oxidative damage as well as deamination, ultimately resulting in asymmetric nucleotide composition between strands (Tanaka and Ozawa 1994; Iliushchenko et al. 2025). In addition, the limited 22 mt‐tRNAs need to translate the full set of 64 possible codons, which would depend on either tRNA import from the nucleus or mt‐tRNA modifications and a unique wobble rule (Tomita et al. 1999; Schneider 2011; Salinas‐Giegé et al. 2015). Codon usage patterns are mainly determined by context‐dependent mutational effects or translational selection of codon‐anticodon pairing (Jia and Higgs 2008; Shtolz and Mishmar 2023). In addition, as the major non‐coding region of the mitogenome, the control region of annelids has shown many features shared with other higher animals, such as conserved sequence blocks, repetitive sequences, and structures associated with its role in the control of mtDNA replication and transcription (Li et al. 2015; Patra et al. 2016). Despite growing interest in mitochondrial evolution, most existing studies are limited to specific taxa such as vertebrates, birds, and arthropods (Shtolz and Mishmar 2023; Black and Roehrdanz 1998; Mindell et al. 1998; Montaña‐Lozano et al. 2022). Comparable relative studies in annelids, especially enchytraeids, remain scarce, and no mitogenome has yet been reported for *Mesenchytraeus*.

In this study, we performed *de novo* assembly, annotation, and comparative analysis of the mitochondrial genomes of 13 *Mesenchytraeus* species (14 individuals) from the Changbai Mountain range. We characterized their genomic organization, gene order, heterogeneity, and nucleotide composition, with a particular focus on codon usage patterns of PCGs, secondary structures of tRNAs, repetitive elements, and secondary structure of putative CR. We also reconstructed the phylogenetic relationships within them. Furthermore, we discussed lineage‐specific differences in nucleotide bias and codon usage patterns across taxa, the unique rearrangement of putative CR within Clitellata, and the possibility that putative CR may contain origins of replication for both the heavy and light strands. This study not only provides the first mitochondrial genome dataset for *Mesenchytraeus* but also provides valuable information regarding much broader patterns of animal mitochondrial evolution.

## 2 Materials and Methods

### 2.1 Sampling and DNA Extraction

Fourteen specimens belonging to thirteen Mesenchytraeus species were collected in Changbai Mountain (Jilin Province, China), representing the ten morphologically distinct species reported for this area—namely the *M. monodiverticulus* complex (comprising *M. fokulen*, *M. monodiverticulus*, *M. ngulen*, and *M. zhenggulen*), the *M. duodiverticulus* complex (comprising *M. duodiverticulus* and *M. similiduodiverticulus*), *M. spermatoglomeratus* (comprising *M. rijina*, *M. spermatoglomeratus*, and *M. manchu*), *M. anisodiverticulus* , *M. gigachaetus*, *M. similigigachaetus*, *M. parvidiverticulus*, *M. digitalisdiverticulus*, *M. infradiverticulus*, and *Mesenchytraeus* sp. (Chen et al. 2024). All specimens were collected in 2020 except for specimen CJJ6, which was collected in 2014 (Table 1). Specimens were anesthetized by adding 30% ethanol dropwise and preserved in 75% ethanol. Specimens were cut into two parts with a scalpel, the anterior part reserved for its morphological study, and the posterior part processed for DNA extraction. Total genomic DNA was extracted using the TIANamp Micro DNA Kit (Tiangen Biotech, Beijing, China) and kept at −80°C for subsequent use.

**TABLE 1:** Specimens' information and GenBank accession number.

Specimen number	GenBank accession number	Species	Locality	Altitude	Sampling time
CJJ6	PV591000	*M. gigachaetus* (Xie 2012)	128°01′18″ E, 42°22′16.8″ N	665 m	2014/12/10
CJJ144	PV591001	*M. spermatoglomeratus* (Zhang et al. 2018)	127°49′21.19″ E, 42°17′39.82″ N	1049 m	2020/10/30
CJJ147	PV591002	*M. similigigachaetus* (Chen et al. 2024)	127°49′21.19″ E, 42°17′39.82″ N	1049 m	2020/10/30
CJJ190	PV591003	*M. duodiverticulus* (Chen et al. 2024)	127°52′51.24″ E, 42°20′7.85″ N	1089 m	2020/10/30
CJJ191	PV591004	*M. duodiverticulus* (Chen et al. 2024)	127°52′51.24″ E, 42°20′7.85″ N	1089 m	2020/10/30
CJJ194	PV591005	*M. fokulen* (Chen et al. 2024)	127°52′51.24″ E, 42°20′7.85″ N	1089 m	2020/10/30
CJJ201	PV591006	*M. digitalisdiverticulus* (Chen et al. 2024)	127°52′51.24″ E, 42°20′7.85″ N	1089 m	2020/10/30
CJJ207	PV591007	*M. ngulen* (Chen et al. 2024)	127°59′39.55″ E, 42°22′1.86″ N	820 m	2020/10/30
CJJ216	PV591008	*M. monodiverticulus* (Shen et al. 2012)	128°05′16.34″ E, 42°24′31.56″ N	756 m	2020/10/30
CJJ225	PV591009	*M. parvidiverticulus* (Chen et al. 2024)	128°05′16.87″ E, 42°24′31.84″ N	760 m	2020/10/30
CJJ228	PV591010	*M. zhenggulen* (Chen et al. 2024)	128°05′16.87″ E, 42°24′31.84″ N	760 m	2020/10/30
CJJ253	PV591011	*Mesenchytraeus* sp.	128°13′28.08″ E, 42°05′20.56″ N	1406 m	2020/10/30
CJJ258	PV591012	*M. infradiverticulus* (Chen et al. 2024)	128°14′01.25″ E, 42°05′40.77″ N	1398 m	2020/10/30
CJJ265	PV591013	*M. anisodiverticulus* (Shen et al. 2012)	128°11′45.23″ E, 42°08′5.85″ N	1299 m	2020/10/30

### 2.2 DNA Libraries Preparation and Whole Genomes Sequencing

A low‐input DNA library of each specimen was prepared by using VAHTSTM Universal DNA Library Prep Kit for MGI (VAZYME, China). Briefly, (1) genomic DNA was fragmented; (2) its fragments converted to blunt‐end DNA through an end‐repair reaction; (3) a single adenosine was added to 3′ ends; (4) library adaptors ligated; and (5) library products amplified through PCR reactions and subjected to quality control processes (BGI‐Shenzhen, China). Next, the final double‐stranded library products were denatured to generate the single‐stranded library products. Then, the circularization reaction was set up to get single‐stranded circularized DNA products. Any single‐stranded linear DNAs were removed by digestion. The final single‐strand circularized library was amplified with phi29 and rolling circle amplification (RCA) to generate the DNA nano ball (DNB), which should carry about 300 copies of the initial single‐stranded library molecule. The DNBs were loaded into the patterned nanoarray, and PE100 sequencing reads were generated using the DNBSEQTM platform (BGI‐Shenzhen, China).

In total, whole‐genome sequences (WGS) were obtained for 14 individuals, each sequenced at an average depth of 20× with three replicates (L02, L03, L04). Raw data were processed by SOAPnuke‐v2.1.04 (parameters: ‐n 0.02 ‐l 20 ‐q 0.4 ‐i ‐G 2 ‐‐polyX 50 ‐Q 2 ‐‐seqType 0) to remove adaptors and low‐quality sequences to get clean data (Chen et al. 2018). The cleaned data were deposited as FASTQ files in NCBI's Sequence Read Archive (SRA) repository under BioProject accession number PRJNA1393209. Accession numbers for the sequencing reads from each specimen are presented in Table 1. The total size of the clean data varied between 13 and 22 GB, with only L02 being used for mitochondrial genome assembly, which ranged from 4 to 7 GB. For individuals for whom the L02 data could not be successfully assembled, the L03 and L04 data were used for auxiliary assembly.

### 2.3 Mitochondrial Genomes Assembly, Annotation and Analysis

*De novo* assembly of the mitochondrial genomes was performed using NOVOPlasty4.3.1 with clean data as input, and the respective *COX1* sequences were used as seeds for extension (Dierckxsens et al. 2017). MEGA X was used to check the beginning and end of the contig sequence to circularize a complete mitochondrial genome after deleting the overlapping sequence (Kumar et al. 2018). The newly assembled mitogenomes were initially annotated with MitoZ 3.4, selecting mitoassemble for assembler, 6 for data size, Annelida‐segmented‐worms for requiring taxa, and default for other parameters (Meng et al. 2019). The automated MitoZ gene identification and annotation were then refined by importing GenBank sequence annotations from *Enchytraeus irregularis* (GenBank accession number: NC_070077) and *Lumbricus terrestris* (GenBank accession number: NC_001673) into a multiple sequence alignment produced by Clustal Omega, followed by a complete inspection of individual gene coverage and reading‐frame matching in each of the new mitogenomes with the aid of MACSE (Ranwez et al. 2011; Sievers et al. 2011; Sievers and Higgins 2018; Sievers and Higgins 2020). PCGs were assumed to end at the first in‐frame full stop codon, or an abbreviated stop codon (UA‐ or U‐‐) ending immediately before the downstream tRNA. Sequences in FASTA format were generally handled using BioEdit v7.0.0 (Hall 1999), and circular maps of the newly assembled mitochondrial genomes were generated using the OGDRAW online platform (https://chlorobox.mpimp‐golm.mpg.de/OGDraw.html).

To generate the sequencing depth and coverage map for the assembled mitogenomes, we mapped the paired‐end reads of each of the three replicates (L02, L03 and L04) of each individual on the corresponding mitogenome sequence, using BWA v0.7.19‐r1273 and SAMtools v1.21 (Li 2013; Danecek et al. 2021); depth and coverage data were plotted with the custom script provided by Ni et al. (2023). The short‐read alignments (BAM files) were then used as input for FreeBayes v1.3.9 to call variants and detect heteroplasmy in all the individual samples, assuming that each one of them contains an indeterminate number of mitochondrial genomes (Garrison and Marth 2012). We used FreeBayes as a frequency‐based pooled caller (−‐pooled‐continuous), setting ploidy level to 1 (−p 1), applying stringent input base and mapping quality filters (−q 30 ‐m 30), amounting to a probability of incorrect base calling or read mapping < 0.001, and keeping all the other parameters at default values, so that we only detected variants above 5% frequency (−‐min‐alternate‐fraction 0.05) (Garrison and Marth 2012). VCF data produced by FreeBayes were additionally filtered by vcflib for their final validation (Garrison et al. 2022). All the validated records should have sequencing depth at least 150×, probability of incorrect mapping < 0.001, probability of incorrect base call < 0.001, no significant deviation from the working hypothesis of no‐strand bias of the alternate allele at the 0.1% level, and no significant deviation from the working hypothesis of no‐placement bias of the alternate allele at the 0.1% level (DP > 150 and MQM > 30 and MQMR > 30 and QR > 30 and QA > 30 and SAP < 30 and EPP < 30). The results of this analysis were also used to resolve ambiguous positions (N's) of the previously assembled reference mitogenome sequences.

### 2.4 Nucleotide Composition Analysis

The nucleotide composition of full mitochondrial genomes, PCGs, rRNAs, and tRNAs was obtained by MEGA X (Kumar et al. 2018). The percentages of AT and GC content were determined by summing either the proportions of A and T, or G and C, respectively. GC and AT skew were determined for the lagging strand (*L*‐strand here: denoted as light, major or *J*‐strand in other publications) of the mitochondrial genome using the following formulas: GC skew = (G—C)/(G + C), and AT skew = (A—T)/(A + T) (Perna and Kocher 1995). The nucleotide composition for each of the three codon positions at each PCG, as well as their corresponding skew values, was obtained using the CAIcal web server (https://ppuigbo.me/programs/CAIcal/) (Puigbò et al. 2008). Scatter plots of GC/AT content and skew were generated using “ggplot2” package in R (https://ggplot2.tidyverse.org).

### 2.5 Codon Usage

The relative synonymous codon usage (RSCU) of 13 PCGs (excluding stop codons) in the mitogenome was analyzed using MEGA X (Kumar et al. 2018; Roth et al. 2012). A heatmap illustrating the RSCU values in the 14 *Mesenchytraeus* mitogenomes was plotted using the “pheatmap” package in R (https://CRAN.R‐project.org/package=pheatmap). The distributions through species of RSCU values for each amino acid for codons complementary and not complementary to the anticodon of a mitochondrial tRNA (mt‐tRNA) were compared by two‐sided Mann–Whitney U‐tests, and corrected for multiple testing using the Benjamini‐Hochberg procedure, setting the false discovery rate to a maximum of 0.01 (Menyhart et al. 2021).

### 2.6 Regulatory Sequences of the Assembled Mitogenomes

Long genome regions (≥ 200 bp) not assigned to specific genes through automatic annotation by MitoZ were assessed as possible control regions (CRs) of mtDNAs. In case a mitogenome contained a single long unassigned region, we preliminarily annotated it as the putative CR, to be subject to further analyses to confirm this role. Thus, first we searched these regions for the presence of repeat elements using Tandem Repeats Finder (https://tandem.bu.edu/trf/home) and Inverted Repeats Finder (https://tandem.bu.edu/irf/home), applying default search parameters. Then, we used the mfold (Zuker 2003) plugin implemented in UGENE (Okonechnikov et al. 2012) to predict the formation of single‐stranded DNA (ssDNA) secondary structures in them. Finally, we carried out an automatic annotation of the origins of replication of the leading‐strand (*O*_*H*_) in the different mitogenomes using the MITOS 2 Galaxy online server (Bernt et al. 2013; Donath et al. 2019; The Galaxy Community 2024), since by definition, true CRs should always contain this kind of regulatory signal, whose nucleotide sequence has different phylogenetically conserved signatures (Xu and Clayton 1995; Saito et al. 2005; Kuhn et al. 2008; Falkenberg et al. 2024) extended over a relatively long stretch of the putative CR (Sahyoun et al. 2014).

Unlike *O*_*H*_, the origin of replication of the lagging‐strand (*O*_*L*_) is not necessarily located within the putative CR. The primary sequence of *O*_*L*_ is not very well conserved, and it appears that tRNA genes may frequently form secondary structures reminiscent of that of the *O*_*L*_, so that they might function as alternative origins of replication (Seligmann et al. 2006). Therefore, we use the variation of nucleotide bias across the genome to infer the position of *O*_*L*_ (Sahyoun et al. 2014; Seligmann et al. 2006). The basic assumption of this method is that, under the strand displacement model of mtDNA replication, the two strands are exposed to an asymmetric mutation process that leads to a positive GC skew in the leading strand (*H*‐strand), which depends on the time spent by that strand in a single‐stranded state (Reyes et al. 1998). Analyses were performed as follows: (1) GC skew was measured on the *L*‐strand, and all sites in each gene arrangement were repositioned relative to the first site immediately downstream of the putative CR (annotated on the *L*‐strand), and continuing along the entire genome in the direction away from the *H‐*strand replication fork. (2) Linear regressions were conducted between the GC skew at third codon positions (GC3) of each PCG and their corresponding relative time spent single‐stranded (*D*_*ssH*_), their outcomes could be compared to find out the most likely candidates to harbor the *O*_*L*_. We focused our analysis on base frequencies at third codon positions because the relative influence of mutation pressure on them is expected to be stronger than at first and second codon positions, which are much more affected by selection on non‐synonymous substitutions. (3) Standard linear regression analysis was performed systematically for all candidate *O*_*L*_ locations, keeping the *O*_*H*_ constantly at the putative CR. The relative goodness‐of‐fit of the different regression lines was measured by comparing their *R* ^2^ (proportion of variance in GC skew that can be explained by *D*_*ssH*_). (4) The significance of the *R* ^2^ obtained for each candidate *O*_*L*_ location was assessed by a permutation test carried out in R (R Core Team 2021), based on the analysis of regression of 1000 simulated datasets produced by randomly reassigning GC skew values to *D*_*ssH*_ values of each original dataset. Regression graphs were produced by Microsoft Excel.

### 2.7 Phylogenetic Analysis

Information regarding the mitogenomes of Clitellata was gathered from the NCBI organelle dataset (https://www.ncbi.nlm.nih.gov/datasets/organelle/?taxon=42113; last accessed July 2, 2025). Phylogenetic relationships among the thirteen newly sequenced species of *Mesenchytraeus* from Changbai Mountain were reconstructed using the concatenated nucleotide sequences of their corresponding PCGs. The data set also included a *M. antaeus* sequence, produced by concatenating GenBank records (KU728854, KU728861, KU639965, KU728868, KU728917, KU728847, KU728840, KU728910, KU728896, KU728903, KU728875, KU728889, KU728882). Additionally, two species were chosen as outgroups: *Enchytraeus irregularis* (mitochondrial genome and annotated information, GenBank accession number: OP381032), and *Enchytraeus crypticus* (unannotated mitochondrial genome, GenBank accession number: CAJHZI010000911). DNA sequences were concatenated by PhyloSuite (Zhang, Gao, et al. 2020). The phylogenetic analysis was conducted using MrBayes v3.2.6 (Ronquist et al. 2012). Bayesian Inference (BI) analysis employed a gamma model of rate variation across sites and sampled across the GTR model space. Analysis was run for 300,000 generations with a sampling frequency of every 500 generations. The first 5% of samples were discarded when the average standard deviation of split frequencies reached 0.01, and the remaining samples were used to build a consensus tree and calculate the posterior probability (PP).

## 3 Results

### 3.1 Mitochondrial Genome Structure and Organization

**FIGURE 1:**
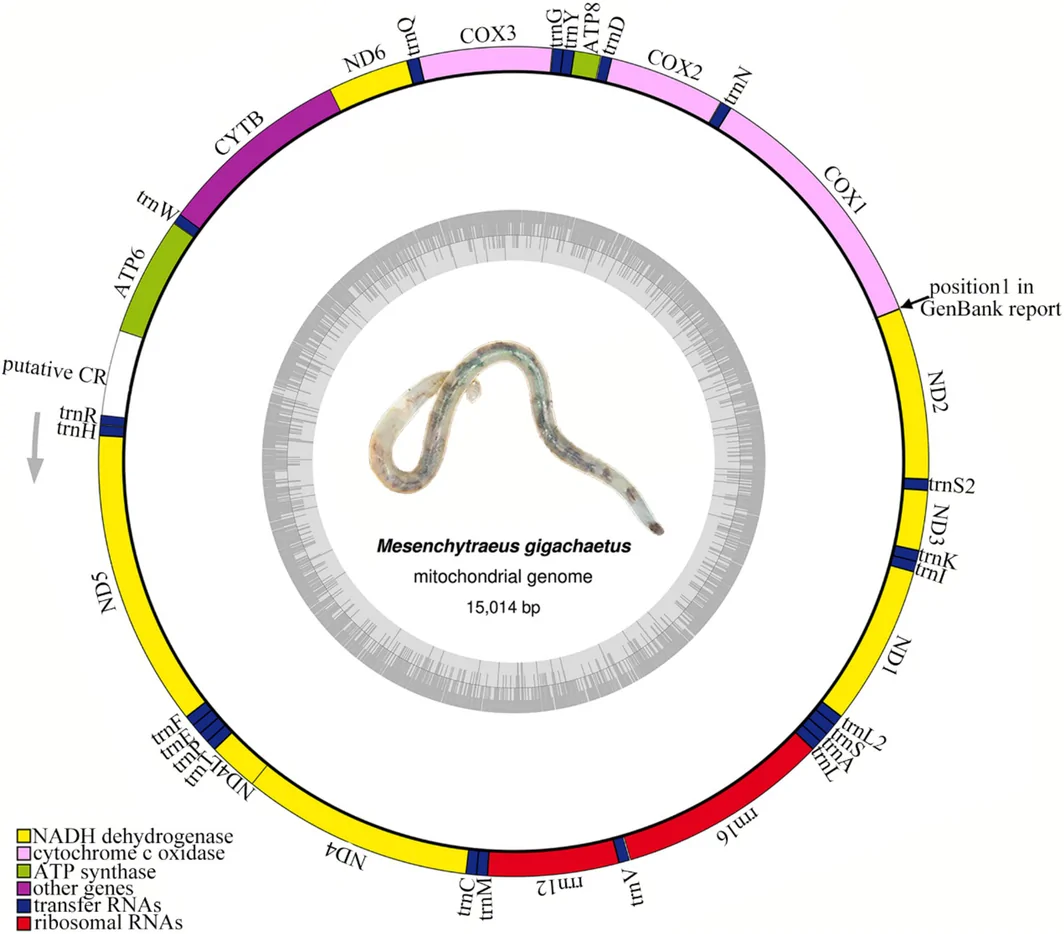
Mitochondrial genome map of *M. gigachaetus*, showing the annotations of the 37 genes and the putative CR on the + strand. Histograms in the inner circle show GC content using the default sliding‐window settings implemented in the OGDRAW online platform. Arrow indicates the direction of transcription from the strand.

The sequencing depth and coverage map of these *de novo* assembled genomes is quite high and homogeneous among the three WGS replicates of each specimen, all of them showing average depths above 300×, and up to 800× in some cases (Figure S1, along with other supplementary data, available at http://datadryad.org/share/LINK_NOT_FOR_PUBLICATION/JBVWh5m_rbhHEiaxk3uj5‐3isG5et85LIiVw44LSrVE). The mitogenomes of all 13 species (14 individuals) were successfully assembled as complete circular molecules (Figure 1), ranging from 14,842 to 15,077 bp in length. All sequences have been deposited in GenBank under accession numbers PV591000–PV591013.

**FIGURE 2:**
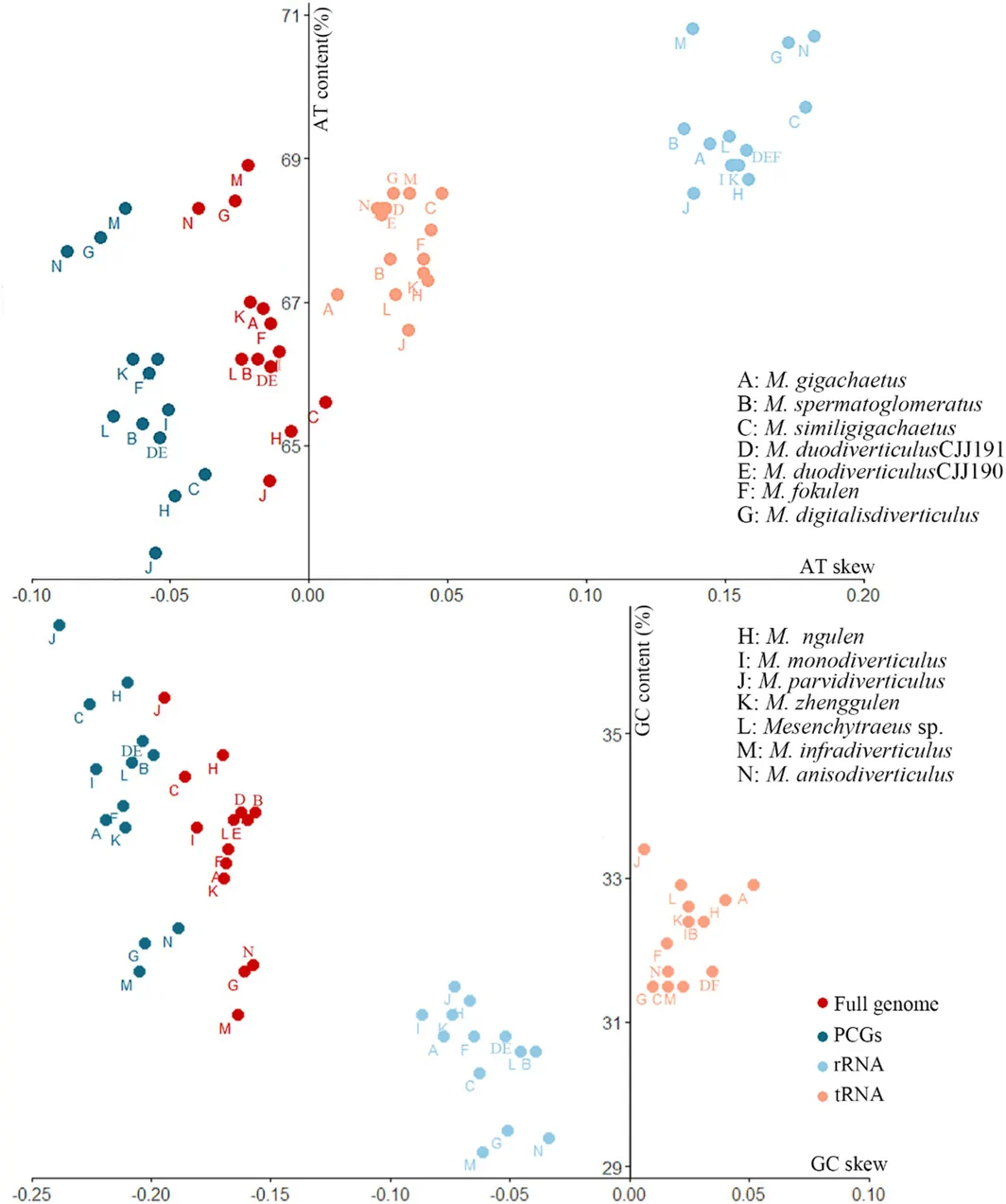
Nucleotide composition (G + C and A + T content, %) and strand asymmetry (GC skew and AT skew) of 14 *Mesenchytraeus* individuals. Points represent the mitogenomes of the different specimens, labeled with letters from A to N, and colored differently depending on the nature of the analyzed sequences (PCGs, rRNA genes, tRNA genes, and whole mitogenomes).

The newly assembled mitogenomes are very similar to each other, all containing the standard set of 37 metazoan mitochondrial genes (13 PCGs, 22 tRNAs, and 2 rRNAs). All genes were annotated on the plus (+) strand, and the gene order was consistent with the dominant annelid pattern (Boore 1999; Zhang et al. 2016; Struck et al. 2023; Shtolz and Mishmar 2023; Zhang et al. 2015). The mitogenomes are highly compact, with intergenic spacers ranging from 0 to 6 bp. All mitogenomes include a long non‐coding region (putative control region, CR) of 232–545 bp located between *ATP6* and *trnR* (Figure 1; Table S1), a position that is unusual within Clitellata, where the CR is typically located between *trnR* and *trnH*. The mitogenomes of *Mesenchytraeus* exhibit adenine and thymine bias, with AT content ranging from 64.5% to 68.9%, AT skew from −0.039 to 0.006, and GC skew from −0.194 to −0.156 (Figure 2).

Heteroplasmy analyses reveal that three species (*M. spermatoglomeratus*, *M. digitalisdiverticulus*, *M. ngulen*) have no heteroplasmy at a minimum detection frequency threshold of 5%. In contrast, *M. anisodiverticulus* harbors a large number of high‐frequency heteroplasmic positions affecting three protein‐coding genes (*COX2*, *ND4*, and *ND1*), 60% of them producing amino acid replacements in the encoded polypeptides. The remaining 10 individuals show 1–6 heteroplasmic variants, mainly concentrated in the putative CR region, usually at frequencies below 20% and not detected in all three sequencing replicates (Table 2; File S1).

**TABLE 2:** List and details of the mtDNA variants detected in the specimens of *Mesenchytraeus*.

Specimen	Species	Position	Depth (*n*)	Mutation	Frequency	Effect	Genomic region
CJJ6	*M. gigachaetus*	5892	358× (2)	A → T	0.043	—	*lur*
		5893	178× (1)	A → T	0.075	—	*lur*
CJJ147	*M. similigigachaetus*	7764	297× (1)	G → C	0.057	S → T	ND5
		11,042	239× (1)	A → T	0.105	rRNA	l‐rRNA
CJJ190	*M. duodiverticulus*	5971	278× (1)	Indel	0.158	—	*lur*
CJJ191	*M. duodiverticulus*	5998	354× (1)	Indel	0.186	—	*lur*
CJJ194	*M. fokulen*	6069	436× (2)	Indel	0.072	—	*lur*
CJJ216	*M. monodiverticulus*	5950	2386× (3)	ATA → CTG	0.210	—	*lur*
CJJ225	*M. parvidiverticulus*	6086	989× (3)	G → A	0.066	—	*lur*
		6396	1359× (3)	A → T	0.080	tRNA	tRNA‐His
CJJ228	*M. zhenggulen*	5922	642× (3)	A → T	0.383	—	*lur*
		5930	892× (3)	A → T	0.235	—	*lur*
		5936	825× (3)	Indel	0.063	—	*lur*
		5981	2603× (2)	C → A	0.433	—	*lur*
		5991	153× (1)	G → A	0.131	—	*lur*
		6149	660× (2)	Indel	0.110	—	*lur*
CJJ253	*Mesenchytraeus* sp.	5847	201× (1)	A → T	0.065	—	*lur*
		5867	168× (1)	C → T	0.083	—	*lur*
		5949	451× (2)	Indel	0.161	—	*lur*
CJJ258	*M. infradiverticulus*	5884	546× (3)	Indel	0.438	—	*lur*
CJJ265	*M. anisodiverticulus*	1745	3123× (3)	C → T	0.530	S → L	COX2
		1753	3148× (3)	A → T	0.530	N → Y	COX2
		1774	3179× (3)	CAAG → AAAA	0.558	Q → K E → K	COX2
		1783	3332× (3)	G → A	0.544	E → K	COX2
		9586	1544× (2)	AG → GA	0.373	Syn A → T	ND4
		9592	1798× (2)	C → T	0.370	Syn	ND4
		9601	2380× (3)	T → A	0.371	Syn	ND4
		9607	2344× (3)	C → T	0.366	Syn	ND4
		9625	1545× (2)	C → A	0.350	F → L	ND4
		9643	2143× (3)	G → A	0.319	Syn	ND4
		9653	2027× (3)	A → T	0.312	T → S	ND4
		9667	1876× (3)	T → C	0.279	Syn	ND4
		9676	1636× (3)	AATAATT→TATAGTC	0.242	Syn I → V	ND4
		9689	1836× (3)	T → C	0.273	F → L	ND4
		9697	1123× (2)	AGCT → GGCA	0.269	Syn Syn	ND4
		9853	1722× (2)	GTGAGC → ATGAAT	0.392	Syn A → I	ND4
		12,859	380× (3)	TCAAT → TAAC	0.190	Frameshift	ND1
		12,869	772× (3)	ATCAT → CTCAC	0.207	M → I W → R	ND1
		12,882	777× (3)	TTG → AAT	0.213	N → L	ND1

*Note:* Mutations are displayed as changes on the + strand. Only validated data from each replicate are taken into account (see Methods). For a complete record of the FreeBayes output, see File S1. The number of replicates (1–3) supporting each variant is indicated in parentheses after the depth data (*n*).

### 3.2 Protein‐Coding Genes (PCGs)

The total length of PCGs ranges from 11,133 to 11,148 bp, accounting for 73.88%–75.07% of the whole genome. The AT content of PCGs is 63.5%–68.3%, and both AT skew and GC skew are negative (Figure 2). The comparison of the three coding sites reveals that the negative AT skew of PCGs actually is driven almost exclusively by the second coding position: all genes and species are characterized by strongly negative AT skews at the second coding position (AT2 skew), but positive values at the third one (AT3 skew), and slightly negative or positive AT1 skew (File S2). Considering genome averages, these differences in nucleotide bias are accompanied by a significantly higher frequency of T at the second site (43%–44%) than at the other two kinds of sites (26%–29% and 30%–37%, at the first and third coding sites, respectively).

Without exception, the translation initiation codons are all conventional AUG (Table S1). Four types of possible stop codons are identified: two complete (UAA and UAG) and two incomplete (U‐‐ and UA‐). Of the two complete codons, the inter‐species comparison shows that UAA is by far the most frequently used (56 out of 58 times), whereas for the two incomplete codons, the U‐‐ is the most frequent (109 out of 124 times) (Table 3). The distribution of these different stop signals among genes is clearly not random: UAA is used preferentially for *COX3*, *ND6*, *ATP6* and *ND4L*, while U‐‐ is the dominant stop for all the others, except for *ND1*, where UA‐ is the most frequent one (Table 3).

**TABLE 3:** Stop codons of mitochondrial protein‐coding genes in the different specimens of *Mesenchytraeus* included in this study.

Genes	CJJ6	CJJ144	CJJ147	CJJ190	CJJ191	CJJ194	CJJ201	CJJ207	CJJ216	CJJ225	CJJ228	CJJ253	CJJ258	CJJ265
	*M. gigachaetus*	*M. spermatoglomeratus*	*M. similigigachaetus*	*M. duodiverticulus*	*M. duodiverticulus*	*M. fokulen*	*M. digitalisdiverticulus*	*M. ngulen*	*M. monodiverticulus*	*M. parvdiverticulus*	*M. zhenggulen*	*Mesenchytraeus* sp.	*M. infradiverticulus*	M. anisodiverticulus
COX1	U‐	U‐	U‐	U‐	U‐	U‐	U‐	U‐	U‐	U‐	U‐	U‐	U‐	U‐
COX2	U‐	U‐	U‐	U‐	U‐	U‐	U‐	U‐	U‐	U‐	U‐	U‐	U‐	U‐
ATP8	U‐	U‐	U‐	U‐	U‐	U‐	U‐	U‐	U‐	U‐	U‐	U‐	U‐	U‐
COX3	U‐	UA	UAA	UAA	UAA	UAA	UAA	UAA	UAA	UAA	UAA	UAA	UAA	UAA
ND6	UAA	UAA	UAA	UAA	UAA	UAA	UAA	UAA	UAA	UAA	UAA	UAA	UAA	UAA
CYTB	U‐	U‐	U‐	U‐	U‐	U‐	U‐	U‐	U‐	U‐	U‐	U‐	U‐	U‐
ATP6	UAA	UAA	UAA	UAA	UAA	UAG	UAG	UAA	UAA	UAA	UAA	UAA	UAA	UAA
ND5	U‐	U‐	U‐	U‐	U‐	U‐	U‐	U‐	U‐	U‐	U‐	U‐	U‐	U‐
ND4L	UAA	UAA	UAA	UAA	UAA	UAA	UAA	UAA	UAA	UAA	UAA	UAA	UAA	UAA
ND4	U‐	U‐	U‐	U‐	U‐	U‐	U‐	U‐	U‐	U‐	U‐	U‐	U‐	UA
ND1	UA	U‐	UAA	UA	UA	UA	UA	UA	UA	U‐	UA	UA	UAA	UAA
ND3	U‐	U‐	U‐	UA	UA	UAA	U‐	U‐	UA	U‐	U‐	UA	U‐	U‐
ND2	U‐	U‐	U‐	U‐	U‐	U‐	U‐	U‐	U‐	U‐	U‐	U‐	U‐	U‐
UAA	3	3	5	4	4	4	3	4	4	4	4	4	5	5
UAG	0	0	0	0	0	1	1	0	0	0	0	0	0	0
UA	1	1	0	2	2	1	1	1	2	0	1	2	0	1
U‐	9	9	8	7	7	7	8	8	7	9	8	7	8	7

**FIGURE 3:**
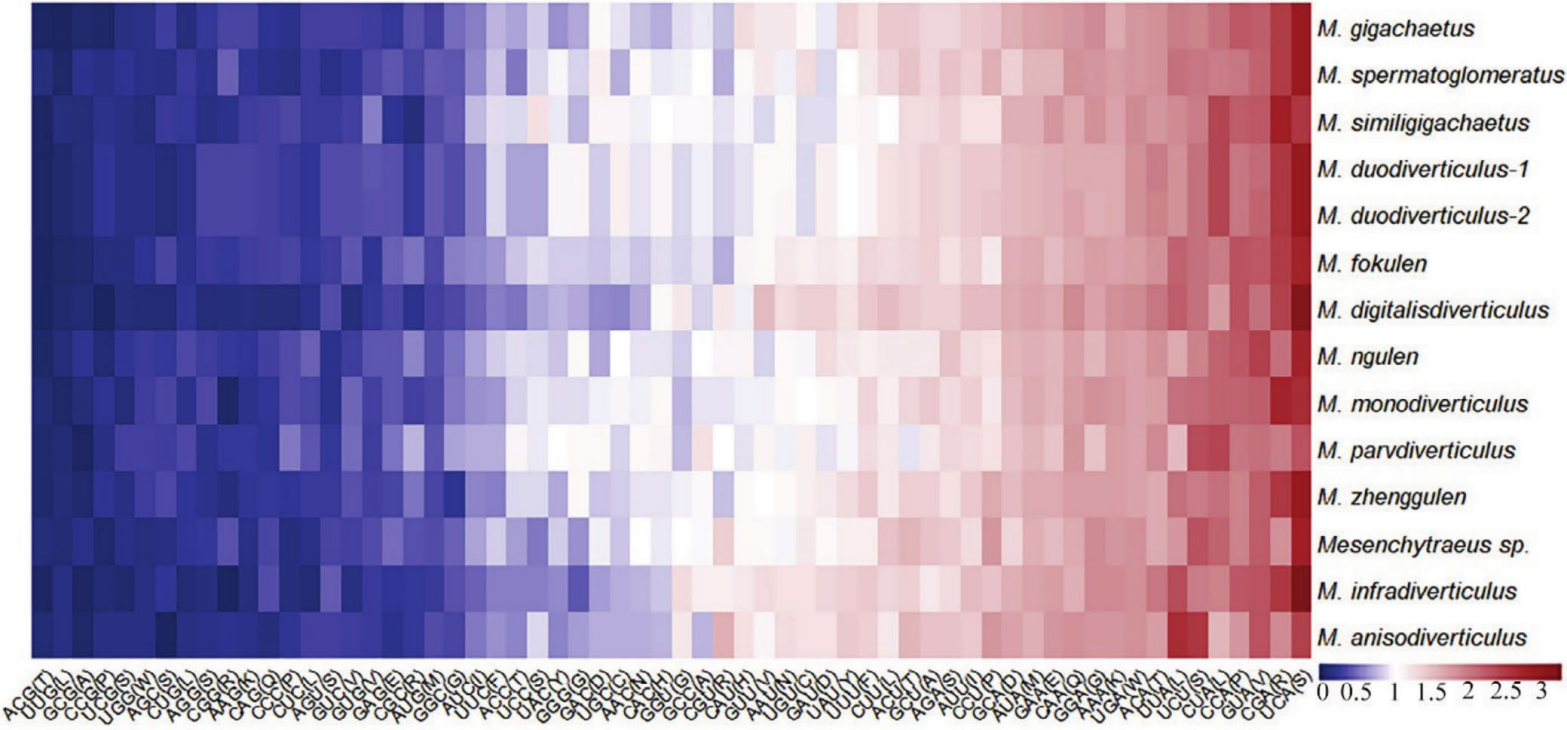
Heat map of RSCU values estimated for each codon across the mitochondrial PCGs of the 14 *Mesenchytraeus* individuals.

Codon usage in *Mesenchytraeus* follows a clear pattern: all codon families with a wobble U are significantly biased toward the corresponding mtDNA codon, whereas all families with a wobble C or G are biased toward non‐mtDNA codons. For instance, Leu, Gln, Lys, Glu, and Trp (2‐codon family A^mt^ + G, wobble U) favor the mtDNA codons UUA, CAA, AAA, GAA, and UGA, respectively. Similarly, in the 4‐codon family A^mt^ + G + U + C (wobble U), all amino acids prefer the mtDNA codons. In contrast, Met (2‐codon family A + G^mt^, wobble C) preferentially uses the non‐mtDNA codon AUA, and in the 2‐codon family U + C^mt^ (wobble G), including Phe, Ile, Tyr, His, Asn, Asp, and Cys, all show significant biases toward non‐mtDNA codons (Figure 3; detailed statistics are provided in File S3).

### 3.3 Transfer RNAs (tRNAs) and Ribosomal RNAs (rRNAs)

**FIGURE 4:**
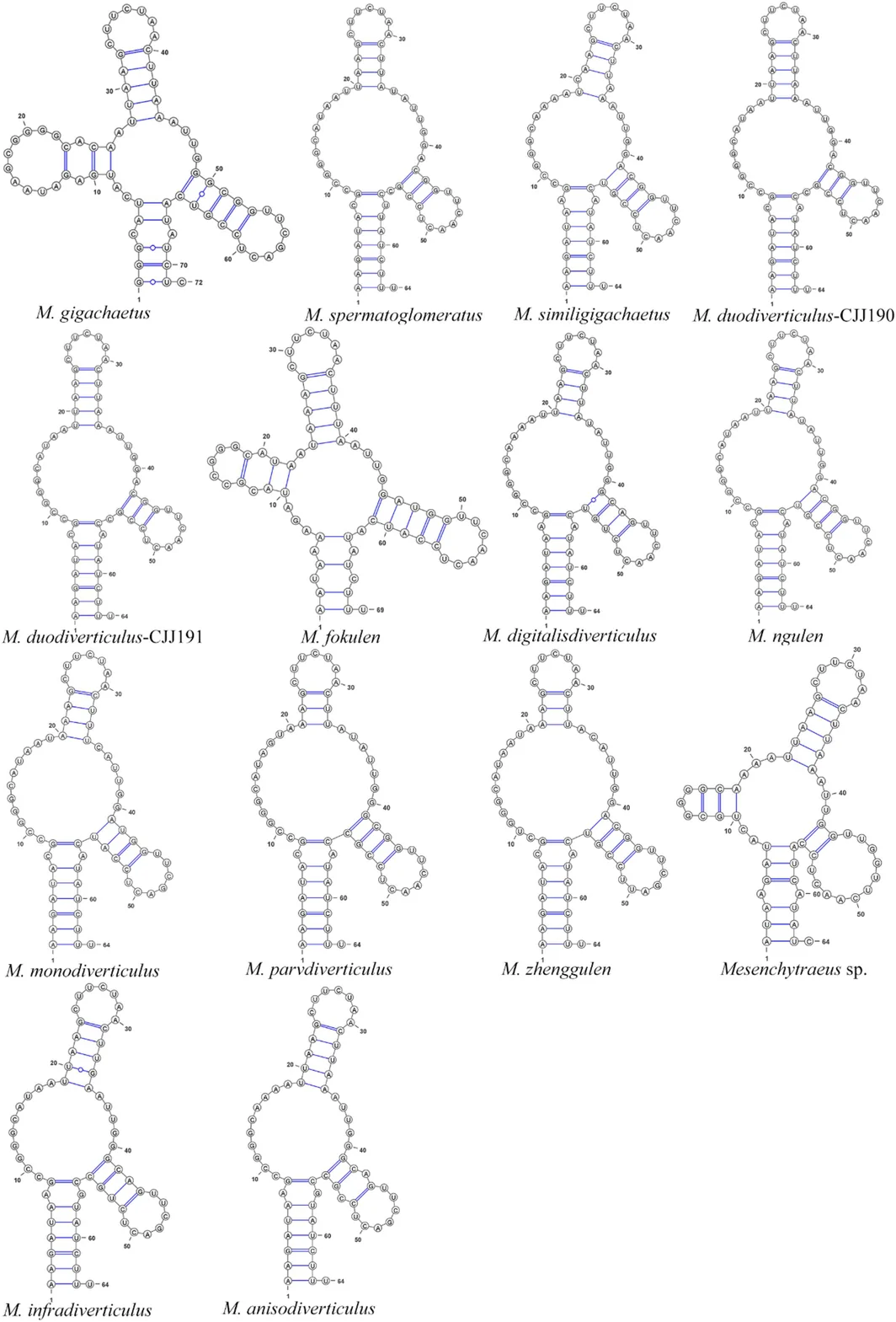
Secondary structure of the *trnS2* of the 14 *Mesenchytraeus* individuals.

The 22 tRNAs of *Mesenchytraeus* species are typical and include all 20 types of amino acids. The total length of the 22 tRNAs ranges from 1369 to 1399 bp, with AT content of 66.6%–68.5%, and both AT and GC skew being positive (Figure 2). Except for *trnS2* (anticodon UCU), all other tRNA genes in *Mesenchytraeus* exhibit a typical cloverleaf structure. The secondary structure of *trnS2* lacks the D‐stem and the dihydrouridine (DHU) arm; a normal *trnS2* secondary structure is only observed in *M. gigachaetus*, *M. fokulen*, and *Mesenchytraeus* sp. (Figure 4).

The AT content of rRNAs in *Mesenchytraeus* (68.5%–70.8%) is much higher than that of PCGs (63.5%–68.3%), with that of tRNAs falling in between (Figure 2). The rRNAs exhibit a high positive AT skew (0.135–0.182) and a low negative GC skew (−0.087 to −0.034) (Figure 2). The 16S rRNA is located between *trnL1* and *trnV*, with a length ranging from 1245 to 1258 bp, and contributes most strongly to the positive AT skew observed in the rRNAs.

### 3.4 Putative Control Region

The mitogenomes of all examined *Mesenchytraeus* species contain a putative control region located between *ATP6* and *trnR*, ranging from 232 to 545 bp in length. These regions are relatively AT‐rich (AT content 63%–73.7%), but not substantially enriched compared to the entire genome. Except for *M. anisodiverticulus* , all species exhibit a positive AT skew, whereas GC skew is negative in all cases. Regarding repetitive elements, tandem repeats (TRs), primarily consisting of (TA)n motifs, are found in six species: *M. gigachaetus*, *M. spermatoglomeratus*, *M. duodiverticulus*, *M. ngulen*, *M. monodiverticulus*, *M. zhenggulen*. Inverted repeats (IR) are detected in four species (*M. gigachaetus*, *M. similigigachaetus*, *M. fokulen*, and *Mesenchytraeus* sp.), corresponding to 25–48 bp long, relatively AT‐poor motifs (Table 4, Tables S2a, S2b). The minimum energy *H*‐strand secondary structures predicted by mfold show that all species contain 1–2 relatively large complex palindromic repeats (CPRs) and 4–9 hairpins (HPs), with the longest and most complex structure located near the 5′ end of the *H*‐strand (Figure S2). In addition, the putative CR of *M. anisodiverticulus* not only lacks the typical complex palindromic repeats, but is also substantially shorter than those of the other species. Combined with the relatively low sequencing depth of this sample, these results suggest that the region may not have been completely assembled.

**TABLE 4:** Feature summary of the putative control regions.

Species	Size (bp)	*O*_*H*_	%(*A* + *T*)	*AT* skew	*GC* skew	Repeat motifs	Secondary structure
*M. gigachaetus*	497	Yes	70.4	0.080	−0.020	(TA)_n_ 22 bp AT‐rich TR 25 bp IR	1 CPR 7 HP
*M. spermatoglomeratus*	478	Yes	69.3	0.111	−0.143	(TA)_n_	2 CPR 6 HP
*M. similigigachaetus*	488	Yes	63.7	0.105	−0.140	25 bp IR	2 CPR 5 HP
*M. duodiverticulus*	442– 469	Yes	68.3 69.9	0.056– 0.066	−0.073– −0.050	(TA)_n_	2 CPR 8 HP
*M. fokulen*	466	Yes	66.8	0.093	−0.033	31 bp IR	1 CPR 7 HP
*M. digitalisdiverticulus*	443	Yes	69.1	0.045	−0.068		2 CPR 5 HP
*M. ngulen*	461	Yes	67.5	0.093	−0.132	(TA)_n_ 21 bp AT‐rich TR	1 CPR 7 HP
*M. monodiverticulus*	525	Yes	71.3	0.077	−0.080	(TA)_n_	2 CPR 6 HP
*M. parvidiverticulus*	457	Yes	63.0	0.048	−0.103		2 CPR 6 HP
*M. zhenggulen*	545	Yes	74.1	0.015	−0.104	(TA)_n_ 37 bp AT‐rich TR 18 bp AT‐rich TR	1 CPR 9 HP
*Mesenchytraeus* sp.	368	Yes	67.6	0.133	−0.077	48 bp IR	8 HP
*M. infradiverticulus*	461	Yes	72.5	0.131	−0.101		1 CPR 4 HP
*M. anisodiverticulus*	232	No	73.7	−0.088	−0.574		7 HP

Abbreviations: CPR, complex palindromic repeat; HP, hairpin; IR, inverted repeat; TR, tandem repeat.

### 3.5 Phylogenetic Analyses

**FIGURE 5:**
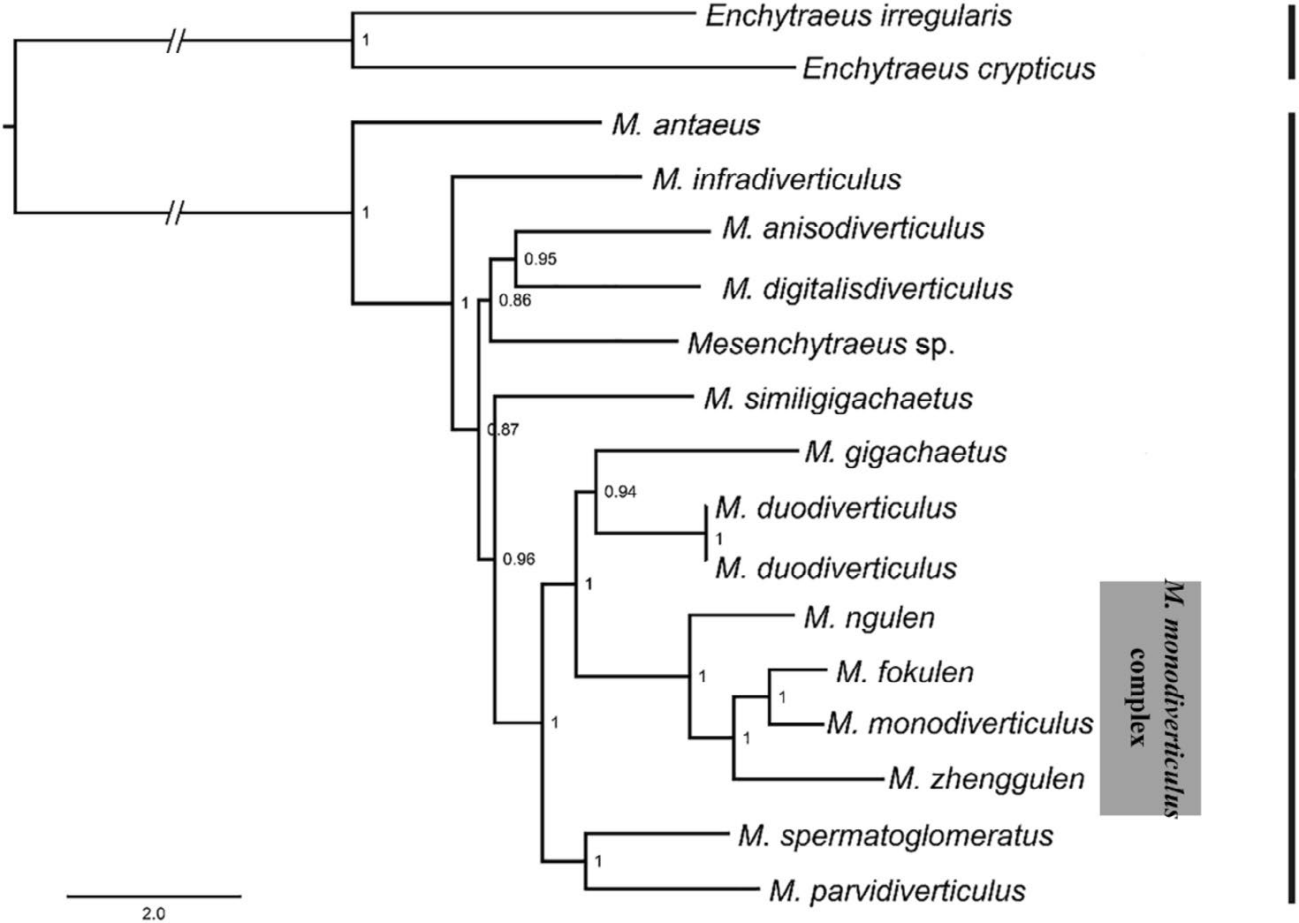
Phylogenetic tree of *Mesenchytraeus* obtained by Bayesian inference (BI), based on the concatenated DNA sequences of the 13 PCGs. Numbers indicate the Bayesian posterior probabilities (BPP) of the corresponding nodes.

In the Bayes phylogenetic tree based on the supermatrix of the 13 PCGs, the monophyly of *Mesenchytraeus* is well supported, given the high posterior probability value (Figure 5) of the branch connecting its species to the *Enchytraeus* outgroups. *M. antaeus* , collected from America, shows a relatively distant phylogenetic relationship to the Chinese lineage, which clearly appears also as a monophyletic group. According to this tree, *M. infradiverticulus* may have been the first to split from the common ancestor, although the corresponding Bayesian posterior probability (BPP) is not excessively high (0.87). Morphologically, this species also exhibits the most significant differences, including entad spermathecal diverticula, the absence of enlarged chaetae, and four pairs of pre‐clitellar nephridia (Chen et al. 2024). Nevertheless, a more rigorous interpretation of the results depicted in that tree (using BPP = 0.95 as a conventional significance threshold) would be that the order of the first splitting events, involving the said *M. infradiverticulus*, *Mesenchytraeus* sp., the common ancestor of the sister species *M. anisodiverticulus* and *M. digitalisdiverticulus*, and the common ancestor of the nine remaining species, is uncertain. This basal polytomy in the Chinese lineage may indicate that the time interval between successive species splits was probably short. Following the same convention and rationale, we can state that there is a significant subclade within that lineage (BPP = 0.96), including *M. similigigachaetus*, *M. gigachaetus*, *M. duodiverticulus*, *M. ngulen*, *M. fokulen*, *M. monodiverticulus*, *M. zhenggulen*, *M. spermatoglomeratus*, and *M. parvidiverticulus*. As far as mitochondrial markers can tell of the evolution of separate species in this subclade, the order of splitting events is rather well defined, often with maximum support (BPP = 1) of the corresponding inner branches of the tree, which indicates relatively longer time intervals between successive speciation events (Figure 5). Even the order of separation of the four sibling species included in the *M. monodiverticulus* complex, which constitute a monophyletic group, can be established with high confidence, thus indicating that a substantial mitochondrial evolution may occur among closely related species despite their relatively limited morphological differentiation.

## 4 Discussion

The + strand of *Mesenchytraeus* mitogenomes is the light strand, and its specific nucleotide biases, characteristic of annelids, are strongly suggestive of evolutionary changes in the rates of deamination of adenine and cytosine. Strand‐specific nucleotide composition bias in mitogenomes can be interpreted as the cumulative consequence of an asymmetric mechanism of mtDNA replication, which violates the base equilibrium expected under intra‐strand parity rule Type 2 (PR2) (Reyes et al. 1998; Tanaka and Ozawa 1994; Iliushchenko et al. 2025; Sueoka 1995). Because they show different buoyant densities in a CsCl gradient, the G‐rich and G‐poor strands have respectively come to be known as heavy (*H*) and light (*L*) strands, and can be quantified using GC skews, so that the G‐poor (*L*) strand shows negative GC skew (Perna and Kocher 1995; Anderson et al. 1981). In *Mesenchytraeus* mitogenomes, the + strand consistently exhibited moderately negative GC skews (−0.194 to −0.156), indicating that this strand corresponds to the *L*‐strand. At the same time, AT skews remain close to zero (−0.040 to +0.006), with only slight deviations observed in a few species. This pattern is consistent with previous observations from other annelid mitogenomes, in which A and T frequencies are near equilibrium (Struck et al. 2023); but differs from the characteristic excess of A over T (i.e., positive AT skew) observed in Chordata (Formaggioni et al. 2021). Assuming that deamination is the main process involved in the observed strand‐specific bias, the two patterns can be explained by differences in the relative rates of adenine and cytosine deamination between the two phyla, for example, simply by an increase in the rate of adenine deamination in Annelida, which in Chordata is only 2%–3% of the rate of cytosine deamination (Hassanin et al. 2005).

The analysis of codon usage in PCGs of *Mesenchytraeus* unveils a clear pattern: biases toward mtDNA codons in codon families with wobble U, but toward non‐mtDNA codons in codon families with wobble G. This pattern is not only observed in the *Mesenchytraeus* species examined in this study, but also highly consistent with other annelids (see Figure S1h in (Shtolz and Mishmar 2023)), suggesting that it may represent a general feature of annelids. Different metazoan phyla show marked differences in codon usage preferences; there are three different general patterns of codon usage in animal mitochondrial PCGs: biased mostly toward mtDNA codons (as in Chordata), mostly toward non‐mtDNA codons (as in Nematoda and Platyhelminthes), or both toward mtDNA codons for codon families with a wobble U and toward non‐mtDNA codons for codon families with a wobble G (as in Annelida) (Shtolz and Mishmar 2023). Notably, Met represents the only 2‐codon family with wobble C and consistently shows a strong preference for the non‐mtDNA codon AUA in all animals. However, the evolutionary driving forces underlying these patterns remain unclear and require further study.

*M. anisodiverticulus* exhibited a markedly different pattern of high‐frequency heteroplasmy from the other species, with some heteroplasmic positions occurring in protein‐coding genes and resulting in nonsynonymous substitutions and even frameshift mutations. This pattern may reflect genuine mitochondrial heteroplasmy, as multiple mtDNA sequence variants can present in a single individual (Parakatselaki and Ladoukakis 2021). Previous studies have shown that nuclear mitochondrial DNA segments (NUMTs) may be misidentified as mitochondrial variants in heteroplasmy analyses (Santibanez‐Koref et al. 2019). In addition, technical factors associated with sequencing and downstream data analysis may affect the detection of heteroplasmic variants (He et al. 2010). In the present study, these variants occurred at relatively high frequencies and involved multiple protein‐coding genes, making it less likely that they resulted solely from low‐frequency random sequencing errors. Nevertheless, the current data cannot distinguish genuine mitochondrial heteroplasmy from NUMTs or other technical factors with certainty. Further validation using independent DNA extractions, targeted resequencing, or long‐read sequencing would help determine the authenticity of these variants (Slapnik et al. 2024).

Previous studies have shown that the position of CR appeared to be conserved in Clitellata so far, lying in between *trnR* and *trnH* (Zhang et al. 2016; Oceguera‐Figueroa et al. 2016; Boore and Brown 1995; Zhao et al. 2022). In contrast, the CR in *Mesenchytraeus* species is consistently located between *ATP6* and *trnR*, suggesting that gene rearrangement of this region has occurred in this genus. The most frequently suggested mechanism of gene rearrangement involves tandem duplication of a certain part of the mitochondrial genome followed by random loss (via pseudogenization and degeneration) of one copy of each of the duplicated genes (Boore and Brown 1998; Struck et al. 2023; Montaña‐Lozano et al. 2022). It is worth mentioning in this respect that hotspots for rearrangement are frequently found near the origin of *L*‐strand replication in both vertebrates and invertebrates (Mindell et al. 1998; San Mauro et al. 2006; Duarte et al. 2008; Fonseca and Harris 2008; Zhang et al. 2021).

The putative CR has the attributes of a genuine control region and most likely contains both origin of replication of *H*‐strand (*O*_*H*_) and *L*‐strand (*O*_*L*_). Except for *M. anisodiverticulus* , the automatic annotation by MITOS 2 of replication origins on the mitogenomes places *O*_*H*_ in the 5′‐end of the *L‐*strand within this region, corresponding to short AT‐rich motifs of 28–133 bp. In addition, no conserved sequence blocks other than the short motifs associated with the *O*_*H*_ were detected within this region, a finding that is not at all exceptional for Clitellata (Zhang et al. 2016). Meanwhile, as predicted by mfold, the longest and most complex structure is located close to the 5′‐end of the *H*‐strand, immediately upstream of the location where *O*_*L*_ would be expected if it were internal to the control region, as is the case in insects (Saito et al. 2005; Arunkumar and Nagaraju 2006). Furthermore, regression analyses between GC3 skew and *D*_*ssH*_ revealed that the best fit was obtained when *O*_*L*_ was assumed to be located within the cluster formed by the putative CR and the two adjacent tRNA genes (*lur*‐RH; *R* ^2^ = 0.593, *p* < 0.001), with GC3 skew showing the expected negative correlation with *D*_*ssH*_ (File S4). This inferred position places *O*_*L*_ at the opposite end of the putative CR relative to *O*_*H*_, providing quantitative evidence that the putative CR may also harbor the origin of replication of the *L*‐strand. Although the exact position of *O*_*L*_ cannot be pinpointed, the available evidence suggests that the putative CR of *Mesenchytraeus* may contain dual replication origins arranged like that of insects, a finding reported for the first time in Annelida.

Phylogenetic analysis provided new mitochondrial genomic evidence for understanding the evolutionary relationships within *Mesenchytraeus*. The node involving *M. infradiverticulus*, *Mesenchytraeus* sp., *M. anisodiverticulus* , and *M. digitalisdiverticulus* received relatively low support. This pattern may reflect a rapid radiation of these early‐diverging lineages, resulting in short internal branches and limited phylogenetic signal for resolving their relationship (Shavit et al. 2007; Whitfield and Lockhart 2007). In contrast, the four species within *M. monodiverticulus* complex formed a well‐supported monophyletic group with a relatively clear internal topology, suggesting that mitochondrial genomes can provide additional molecular resolution for phylogenetic relationships among morphologically similar lineages. This result also complements the species boundaries previously recognized based on an integrative taxonomic approach (Chen et al. 2024). Overall, our phylogenetic results support several well‐resolved relationships within the Chinese *Mesenchytraeus* lineage, while some basal relationships remain uncertain. Broader taxon sampling and additional independent molecular markers will be valuable for further resolving the early evolutionary history of the genus.

## Author Contributions

**Juanjuan Chen:** conceptualization (equal), formal analysis (equal), software (equal), visualization (equal), writing – original draft (equal), writing – review and editing (equal). **Horacio Naveira:** conceptualization (equal), formal analysis (equal), methodology (equal), software (equal), visualization (equal), writing – original draft (equal), writing – review and editing (equal). **Junqian Zhang:** resources (equal), writing – review and editing (equal). **Naicheng Wu:** writing – review and editing (equal). **Zhicai Xie:** funding acquisition (equal), project administration (equal), resources (equal), supervision (equal), writing – review and editing (equal).

## Funding

This work was supported by the Special Foundation for National Science and Technology Basic Research Program of China (2022FY100400), the National Natural Science Foundation of China (grant 32271664 and 32301370).

## Conflicts of Interest

The authors declare no conflicts of interest.

## Supporting information


**Figure S1:** The sequencing depth and coverage for all individuals included in this study.

**Figure S2:** H‐strand secondary structures of the long unassigned regions putatively corresponding to the control regions of the different species of Mesenchytraeus. Shown plots correspond to the minimum energy structures predicted by mfold (Zuker 2003).

**Table S1:** Organization and structure of the mitochondrial genomes of the different specimens of Mesenchytraeus studied included in this study.

**Table S2a:** Tandem Repeat Finder output for the DL sequence of Mesenchytraeus species.

**Table S2b:** Inverted Repeat Finder output for the DL sequence of Mesenchytraeus species.

## Data Availability

All the required data have been made publicly available. The complete mitochondrial genomes of *Mesenchytraeus* were deposited in the GenBank of NCBI (https://www.ncbi.nlm.nih.gov) and obtained the accession number (GenBank accession no. PV591000–PV591013). All Supporting Information materials have been uploaded into Dryad under the following: http://datadryad.org/share/LINK_NOT_FOR_PUBLICATION/JBVWh5m_rbhHEiaxk3uj5‐3isG5et85LIiVw44LSrVE.
