# Supplementary material for: De Novo Assembly and Comparative Analysis of the Complete Mitochondrial Genome of Mesenchytraeus (Annelida, Enchytraeidae)
Source: Ecol Evol. 2026 Sep 4;16(9):e74293. doi: 10.1002/ece3.74293 (PMC13543304; doi:10.1002/ece3.74293)
Supplement: Supplementary file 1 [file ECE3-16-e74293-s001.pdf]

Table S2a. Tandem Repeat Finder output for the DL sequence of *Mesenchytraeus* species

```
Please cite:
G. Benson,
"Tandem repeats finder: a program to analyze DNA sequences"
Nucleic Acid Research(1999)
Vol. 27, No. 2, pp. 573-580.

Sequence: gigachaetus-DL
Parameters: 2 7 7 80 10 50 500
Length:  497

Tables:   1

This is table  1  of  1  ( 2 repeats found )

Click on indices to view alignment
```

Table Explanation

Indices	Period Size	Copy Number	Consensus Size	Percent Matches	Percent Indels	Score	A	C	G	T	Entropy (0-2)
10--104	2	48.0	2	74	10	79	46	3	2	48	1.30
13--62	22	2.3	22	100	0	100	46	4	4	46	1.40

```
Indices: 10--104  Score: 79
Period size: 2  Copynumber: 48.0  Consensus size: 2

   1 TTTTTTTAG

                   *        *       *                *        *
  10 TA TA TA TA TGC TA TA TT TA TA AA TA -A TA TA TGC TA TA TT TA TA
   1 TA TA TA TA T-A TA TA TA TA TA TA TA TA TA TA T-A TA TA TA TA TA

     *                                       *             *
  53 AA TA -A TA TA TA TA TA TA TA TA TA T- TT TA TA TA TA CA TA TA
   1 TA TA TA TA TA TA TA TA TA TA TA TA TA TA TA TA TA TA TA TA TA

  93 TA TA TA TA TA TA
   1 TA TA TA TA TA TA

 105 CATACAGGCC
```

```
Indices: 13--62  Score: 100
Period size: 22  Copynumber: 2.3  Consensus size: 22

   3 TTTTTAGTAT

  13 ATATATGCTATATTTATAAATA
   1 ATATATGCTATATTTATAAATA

  35 ATATATGCTATATTTATAAATA
   1 ATATATGCTATATTTATAAATA

  57 ATATAT
   1 ATATAT

  63 ATATATATAT
```

```
Please cite:
G. Benson,
"Tandem repeats finder: a program to analyze DNA sequences"
Nucleic Acid Research(1999)
Vol. 27, No. 2, pp. 573-580.

Sequence: spermatoglomeratus-DL
Parameters: 2 7 7 80 10 50 500
Length:  478

Tables:   1

This is table  1  of  1  ( 1 repeats found )

Click on indices to view alignment
```

Table Explanation

Indices	Period Size	Copy Number	Consensus Size	Percent Matches	Percent Indels	Score	A	C	G	T	Entropy (0-2)
55--80	2	13.0	2	100	0	52	50	0	0	50	1.00

```
Indices: 55--80  Score: 52
Period size: 2  Copynumber: 13.0  Consensus size: 2

  45 AAATAAATTA

  55 AT AT AT AT AT AT AT AT AT AT AT AT AT
   1 AT AT AT AT AT AT AT AT AT AT AT AT AT

  81 CACAGCTATA
```

```
Sequence: duodiverticulus_CJJ191-DL
Parameters: 2 7 7 80 10 50 500
Length:  469

Tables:   1

This is table  1  of  1  ( 1 repeats found )

Click on indices to view alignment
```

Table Explanation

Indices	Period Size	Copy Number	Consensus Size	Percent Matches	Percent Indels	Score	A	C	G	T	Entropy (0-2)
29--71	2	21.0	2	90	4	68	46	0	4	48	1.22

```
   Indices: 29--71  Score: 68
   Period size: 2  Copynumber: 21.0  Consensus size: 2

        19 ATAATTACTA

                                      *
        29 AT AT AT AT AT AT AT AT AT AT GT AT AT GAT AT AT AT AT AT AT AT
         1 AT AT AT AT AT AT AT AT AT AT AT AT AT -AT AT AT AT AT AT AT AT

        72 GGATGGCCTA
```

```
Sequence: ngulen-DL
Parameters: 2 7 7 80 10 50 500
Length:  461

Tables:   1

This is table  1  of  1  ( 2 repeats found )

Click on indices to view alignment
```

Table Explanation

Indices	Period Size	Copy Number	Consensus Size	Percent Matches	Percent Indels	Score	A	C	G	T	Entropy (0-2)
23--64	2	22.0	2	85	9	61	47	0	2	50	1.14
23--64	21	2.0	21	90	0	66	47	0	2	50	1.14

```
 Indices: 23--64  Score: 61
 Period size: 2  Copynumber: 22.0  Consensus size: 2

      13 TTAAATATTT

                                                            *
      23 TA TA -A TA TA TA TA TA TA TA TA TA TA T- TA TA TA TA TG TA TA
       1 TA TA TA TA TA TA TA TA TA TA TA TA TA TA TA TA TA TA TA TA TA


      63 TA
       1 TA

      65 CGTAAGACAT
```

```
 Indices: 23--64  Score: 66
 Period size: 21  Copynumber: 2.0  Consensus size: 21

      13 TTAAATATTT


      23 TATAATATATATATATATATA
       1 TATAATATATATATATATATA

             *         *
      44 TATATTATATATATGTATATA
       1 TATAATATATATATATATATA

      65 CGTAAGACAT
```

```
Sequence: monodiverticulus-DL
Parameters: 2 7 7 80 10 50 500
Length:  525

Tables:   1

This is table  1  of  1  ( 1 repeats found )

Click on indices to view alignment
```

Table Explanation

Indices	Period Size	Copy Number	Consensus Size	Percent Matches	Percent Indels	Score	A	C	G	T	Entropy (0-2)
50--129	2	40.0	2	87	0	106	42	0	5	50	1.24

```
Indices: 50--129  Score: 106
Period size: 2  Copynumber: 40.0  Consensus size: 2

     40 ACAGAGAATG

               *                    *                       *
     50 TA TA TG TA TA TA TA TA TA TG TA TA TA TA TA TA TA TG TA TA TA
      1 TA TA TA TA TA TA TA TA TA TA TA TA TA TA TA TA TA TA TA TA TA

            *                 *  *
     92 TA TG TA TA TA TA TA TR TR TA TA TA TA TA TA TA TA TA TA
      1 TA TA TA TA TA TA TA TA TA TA TA TA TA TA TA TA TA TA TA

    130 CGGGCCCATG
```

Sequence: zhenggulen-DL
Parameters: 2 7 7 80 10 50 500
Length: 545

Tables: 1

This is table 1 of 1 (3 repeats found)

Click on indices to view alignment

Table Explanation

Indices	Period Size	Copy Number	Consensus Size	Percent Matches	Percent Indels	Score	A	C	G	T	Entropy (0-2)
18--148	2	66.5	2	76	11	110	42	3	3	51	1.33
18--148	37	4.1	32	77	15	121	42	3	3	51	1.33
89--168	18	4.5	18	85	4	65	38	3	7	50	1.49

```
Indices: 18--148  Score: 110
Period size: 2  Copynumber: 66.5  Consensus size: 2

      8 TATGTAAATT

                  *                         *     *
     18 TA TA TA TT TA TA TA TA TA TA TA -A CA -A AA TA TA T- TCA TA TA
      1 TA TA TA TA TA TA TA TA TA TA TA TA TA TA TA TA TA TA T-A TA TA

                     *            *        *           *
     58 T- TA TA TA TG TA TA CT- TG TA TA TT TA TA TA TT TA TA TA GTA TA
      1 TA TA TA TA TA TA TA -TA TA TA TA TA TA TA TA TA TA TA TA -TA TA

            *                          *                          *
    100 TA TG TA TA TA TA TA TA TA TA TT TA TA TA TA TA TA TA TA TC TA
      1 TA TA TA TA TA TA TA TA TA TA TA TA TA TA TA TA TA TA TA TA TA


    142 TA TA TA T
      1 TA TA TA T

    149 GTATGCATCT
```

```
Indices: 18--148  Score: 121
Period size: 37  Copynumber: 4.1  Consensus size: 32

      8 TATGTAAATT

                                  *
     18 TATATATTTATATATATATA--TA-ACA-A-A
      1 TATATATTTATATATATATATGTATATATATA

                *                           *
     45 -ATATATTCATATATTATATATGTATACTTGTATATT
      1 TATATATTTATATA-TATATATGTATA----TATATA


     81 TATATATTTATATAGTATATATGTATATATATA
      1 TATATATTTATATA-TATATATGTATATATATA

                                 *   *
    114 TATATATTTATATATATATATATATCTATATA
      1 TATATATTTATATATATATATGTATATATATA


    146 TAT
      1 TAT

    149 GTATGCATCT
```

```
Indices: 89--168  Score: 65
Period size: 18  Copynumber: 4.5  Consensus size: 18

     79 TTTATATATT

                    *  *
     89 TATATA-GTATATATGTA
      1 TATATATGTATACATCTA

               *    *  *
    106 TATATATATATATATTTA
      1 TATATATGTATACATCTA

               *    *
    124 TATATATATATATATCTA
      1 TATATATGTATACATCTA

                   *
    142 TATATATGTATGCATCTA
      1 TATATATGTATACATCTA


    160 GT-TATATGT
      1 -TATATATGT

    169 GCCTATGGGG
```
