# Supplementary material for: De Novo Assembly and Comparative Analysis of the Complete Mitochondrial Genome of Mesenchytraeus (Annelida, Enchytraeidae)
Source: Ecol Evol. 2026 Sep 4;16(9):e74293. doi: 10.1002/ece3.74293 (PMC13543304; doi:10.1002/ece3.74293)

Supplementary Figure S1. The sequencing depth and coverage for all individuals included in this study

M. gigachaetus L02

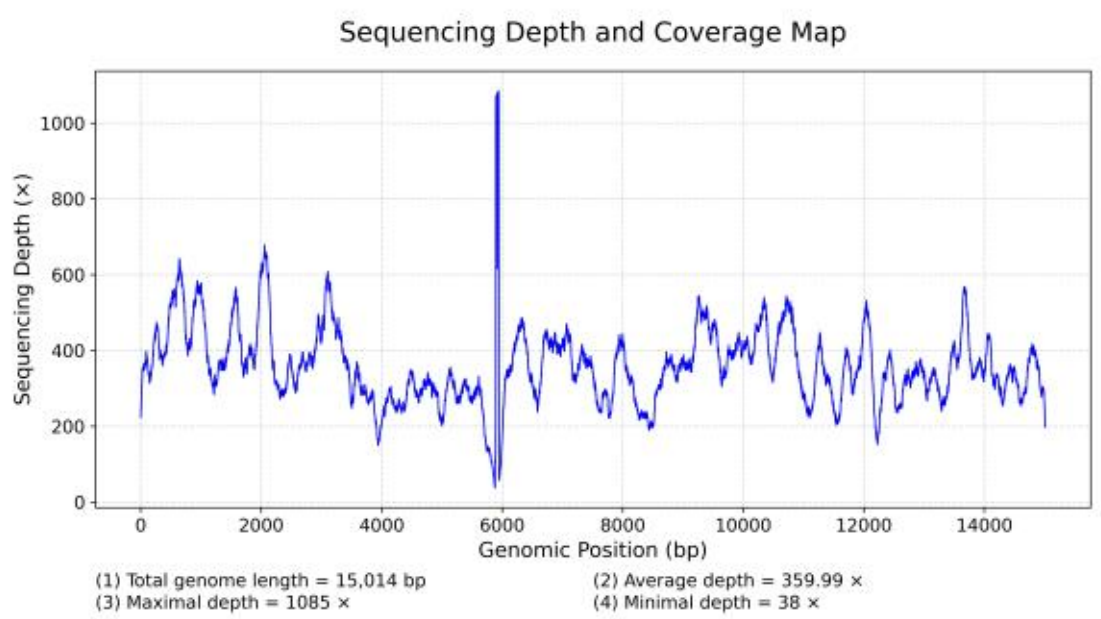

M. gigachaetus L03

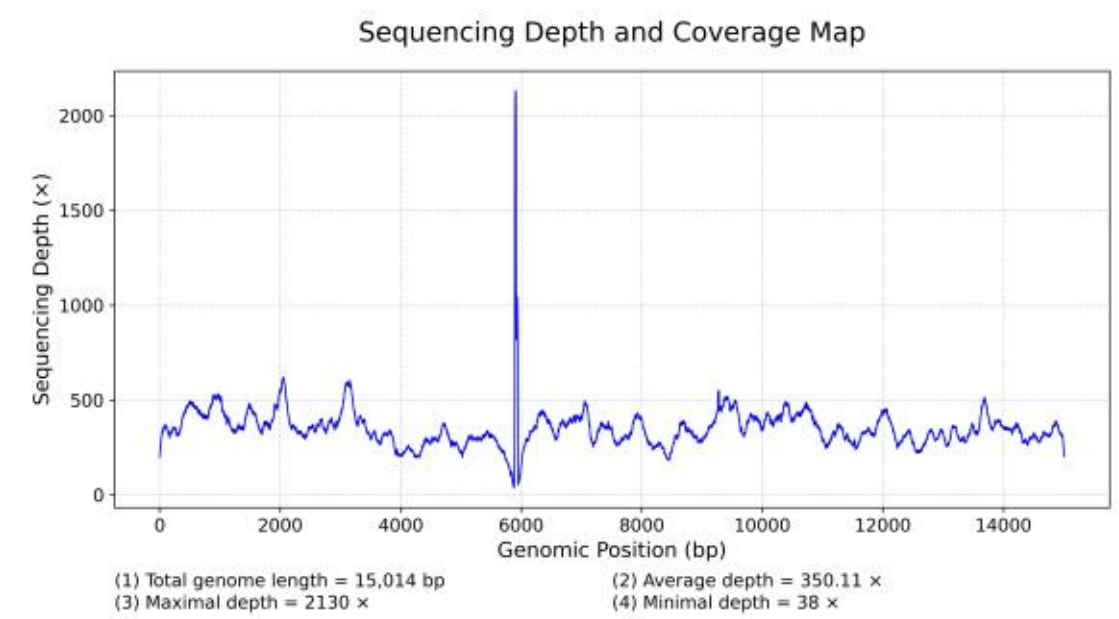

M. gigachaetus L04

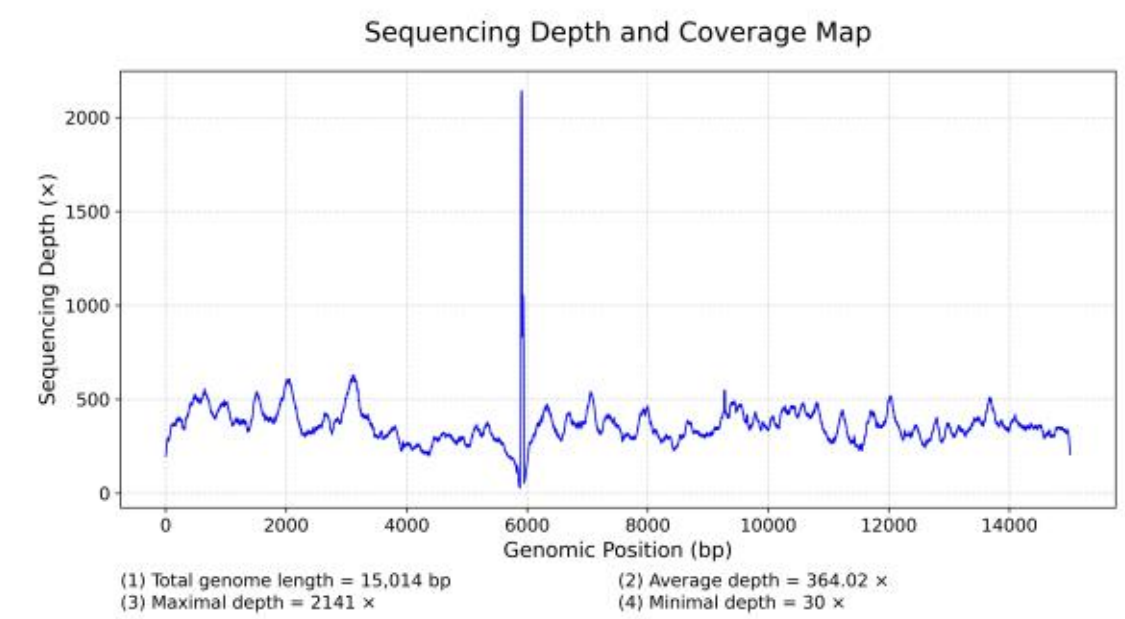

M. spermatoglomeratus L02

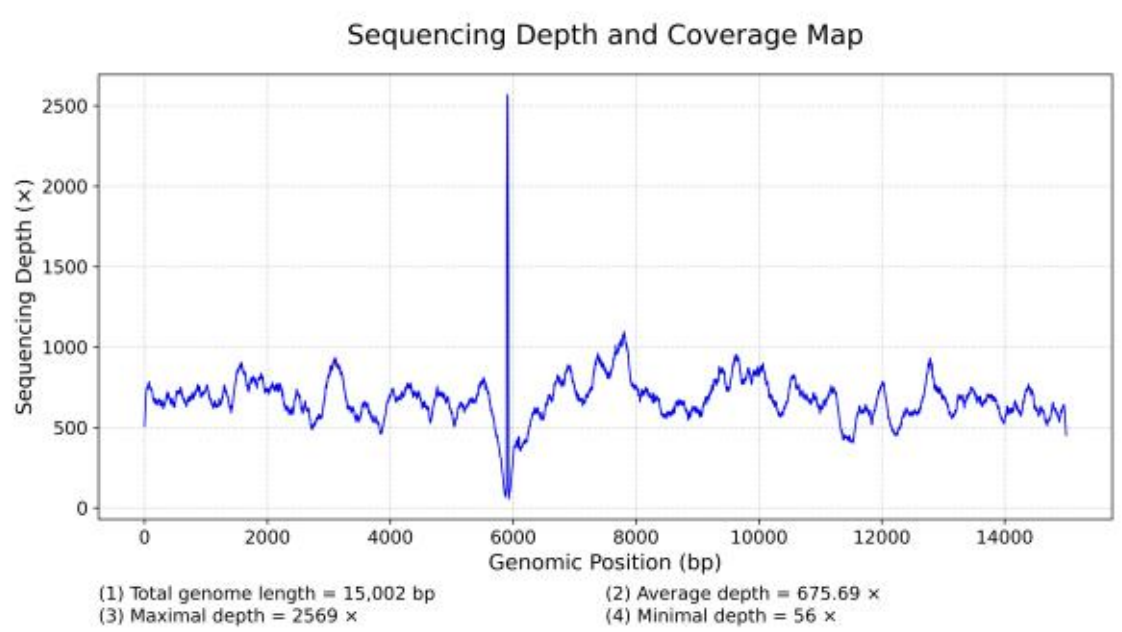

M. spermatoglomeratus L03

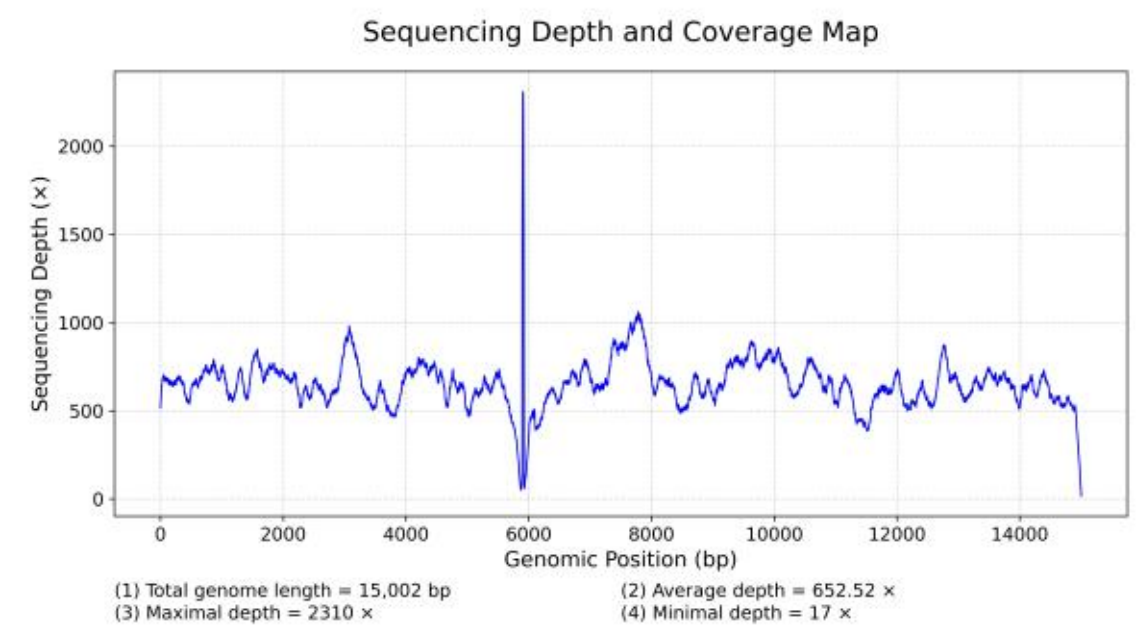

M. spermatoglomeratus L04

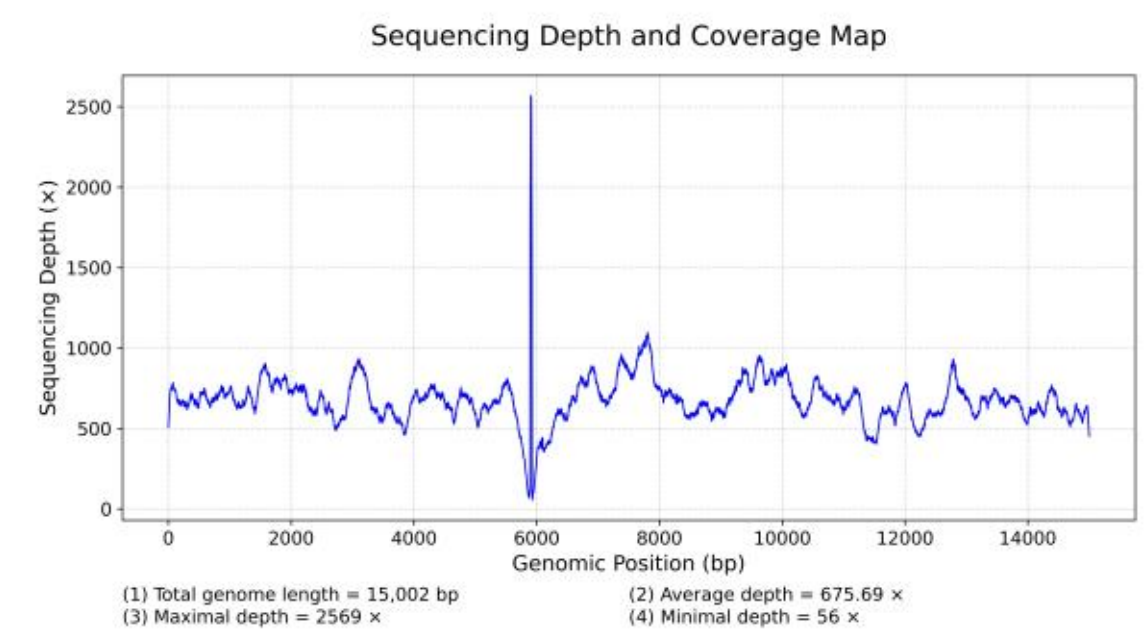

M. similigigachaetus L02

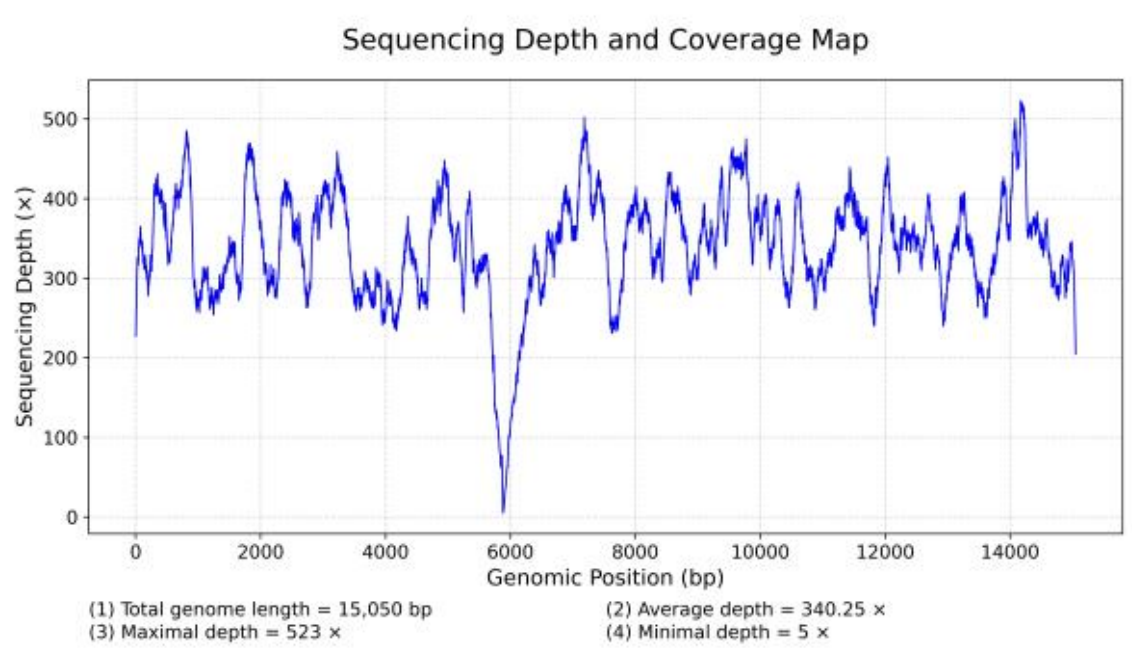

M. similigigachaetus L03

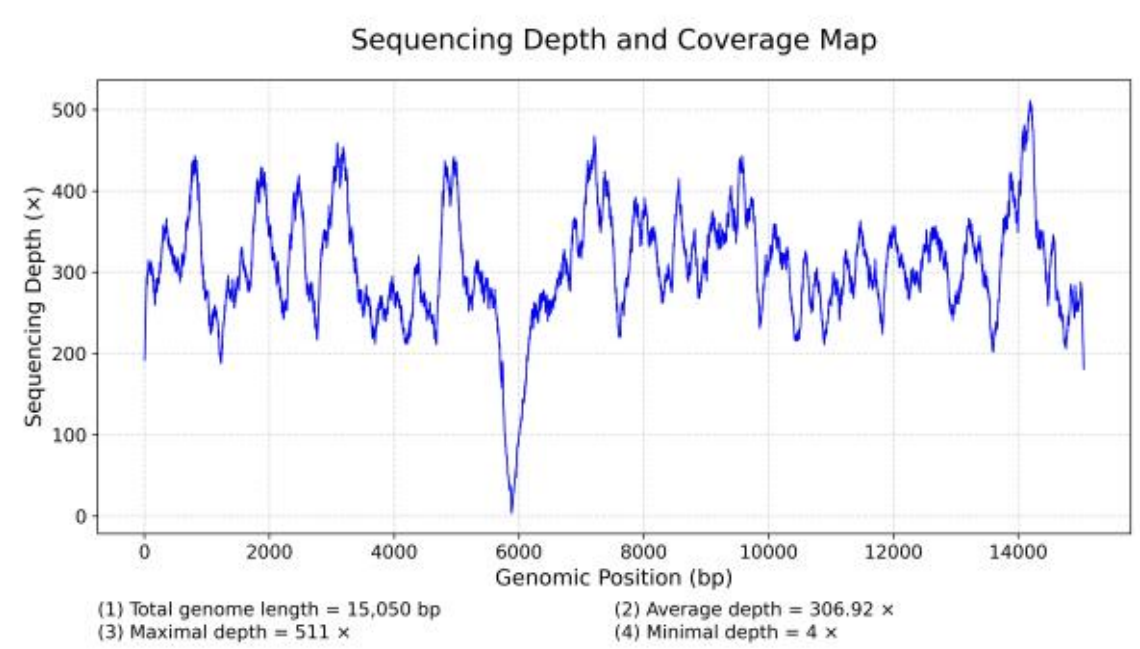

M. similigigachaetus L04

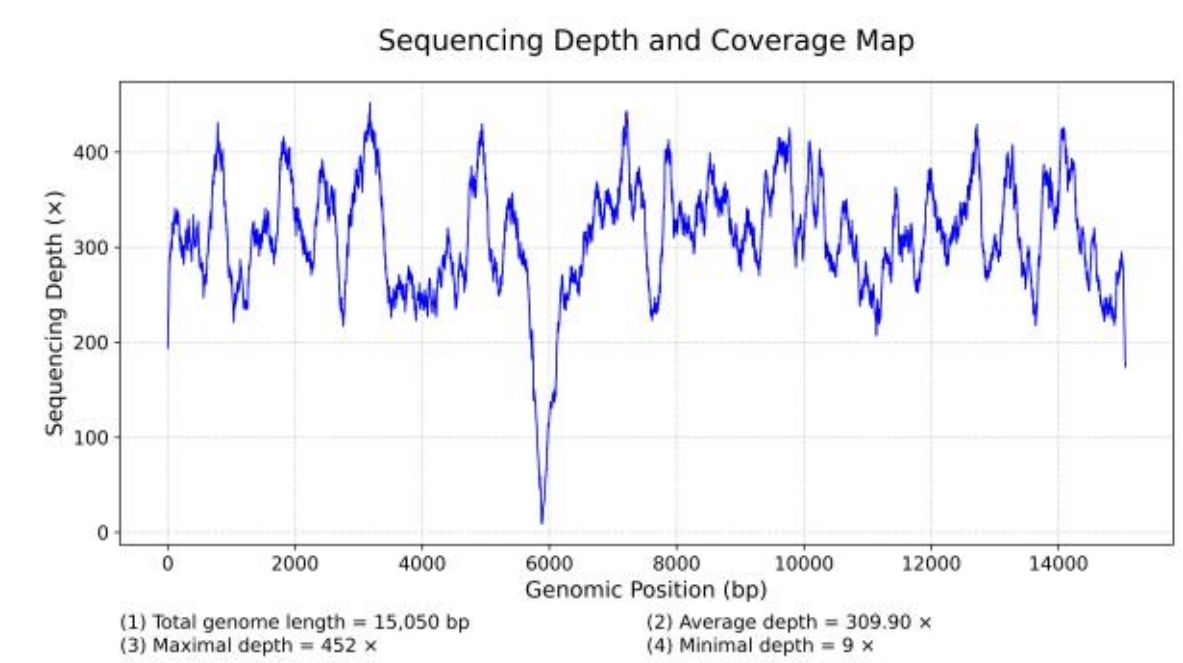

M. duodiverticulus CJJ190 L02

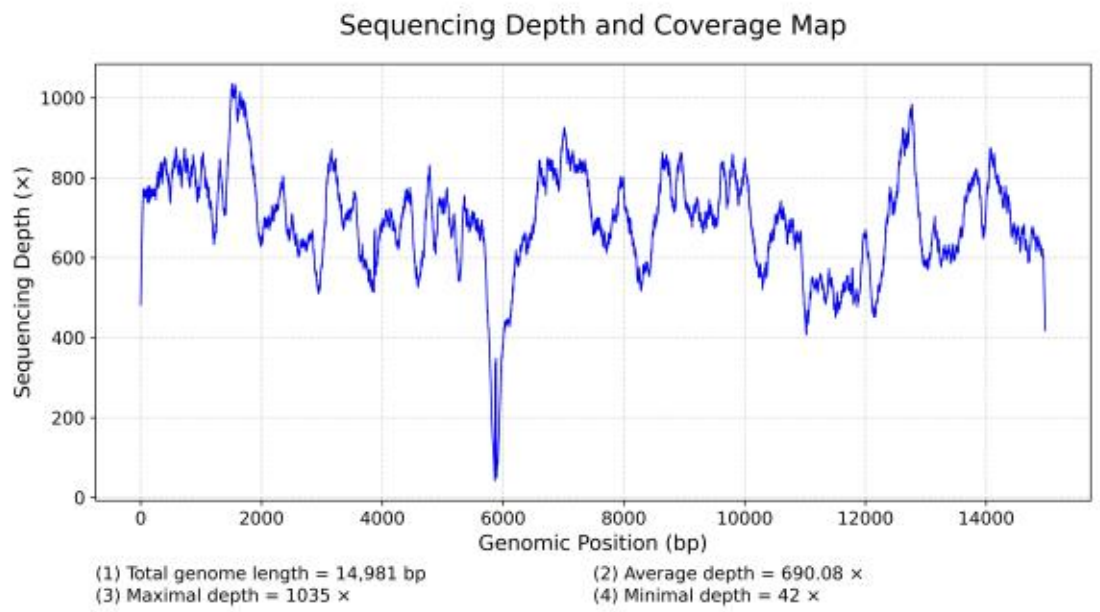

M. duodiverticulus CJJ190 L03

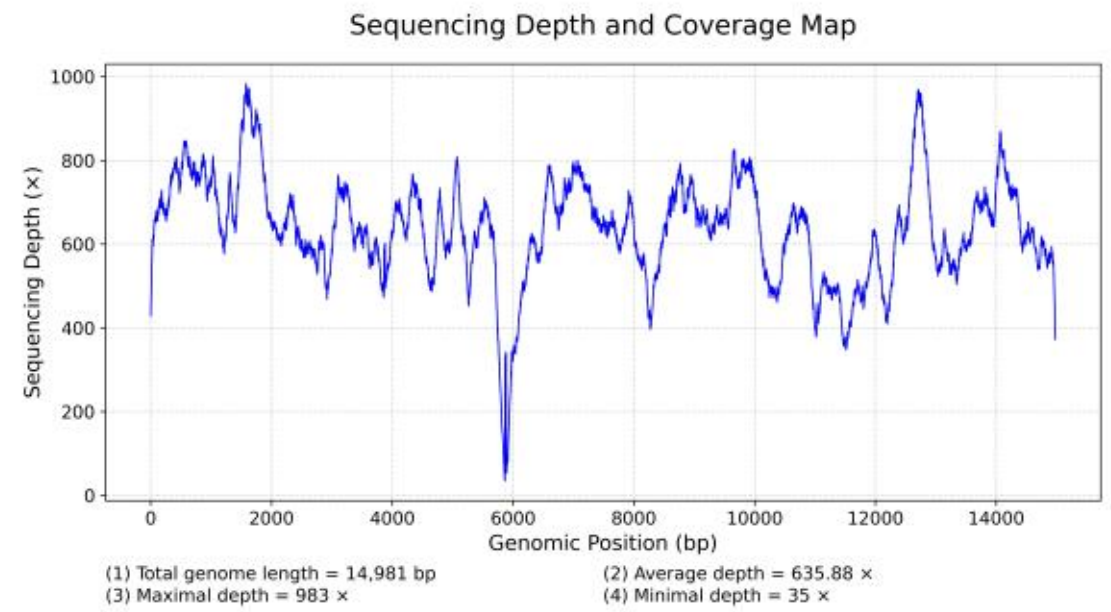

M. duodiverticulus CJJ190 L04

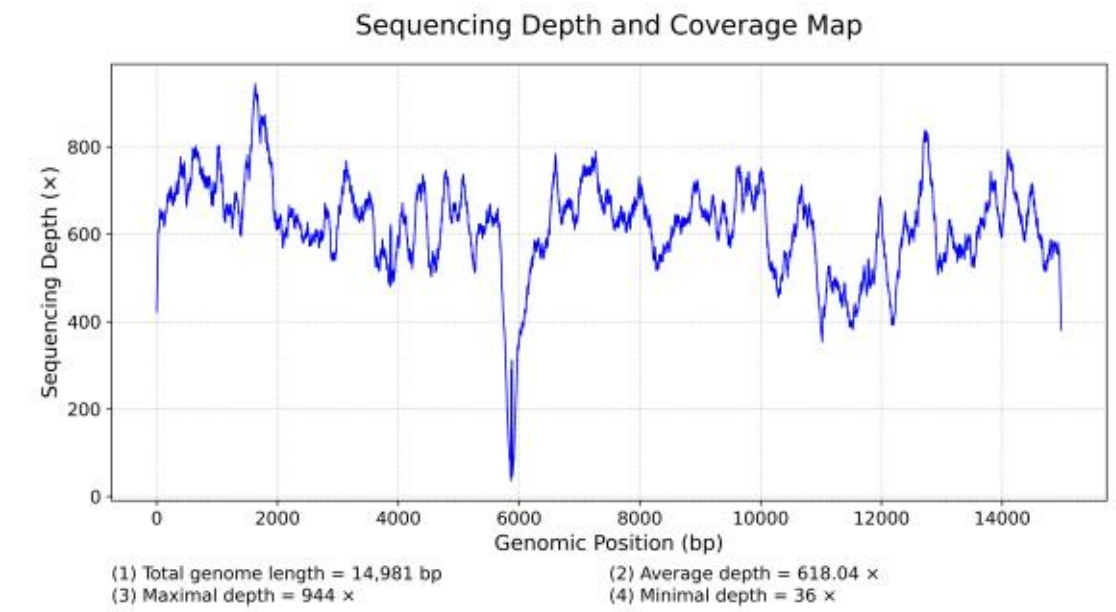

M. duodiverticulus CJJ191 L02

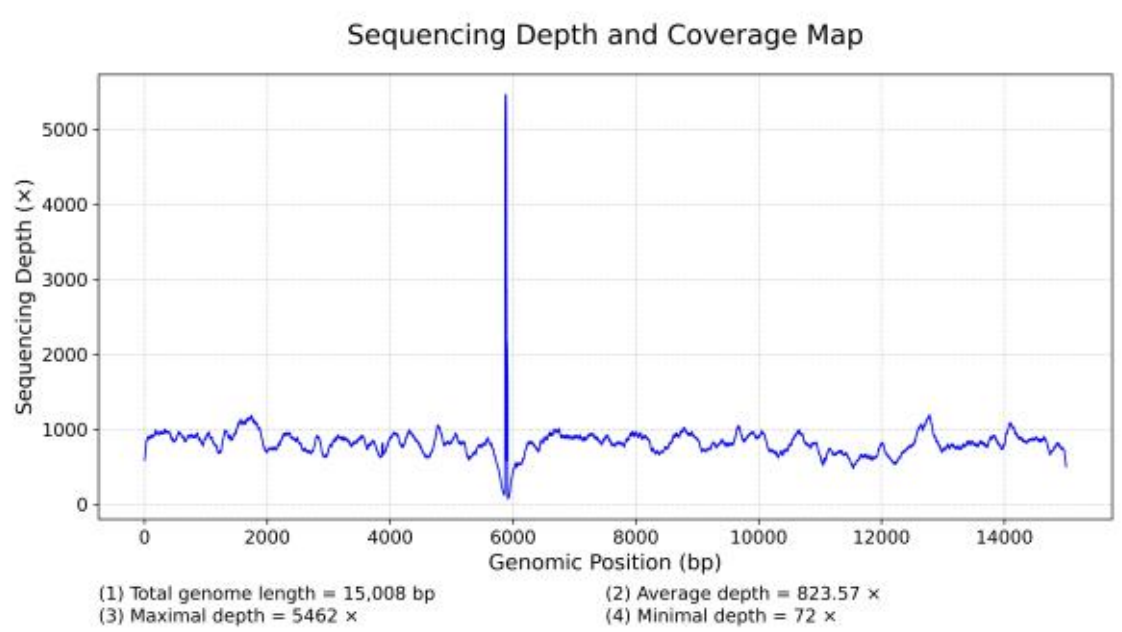

M. duodiverticulus CJJ191 L03

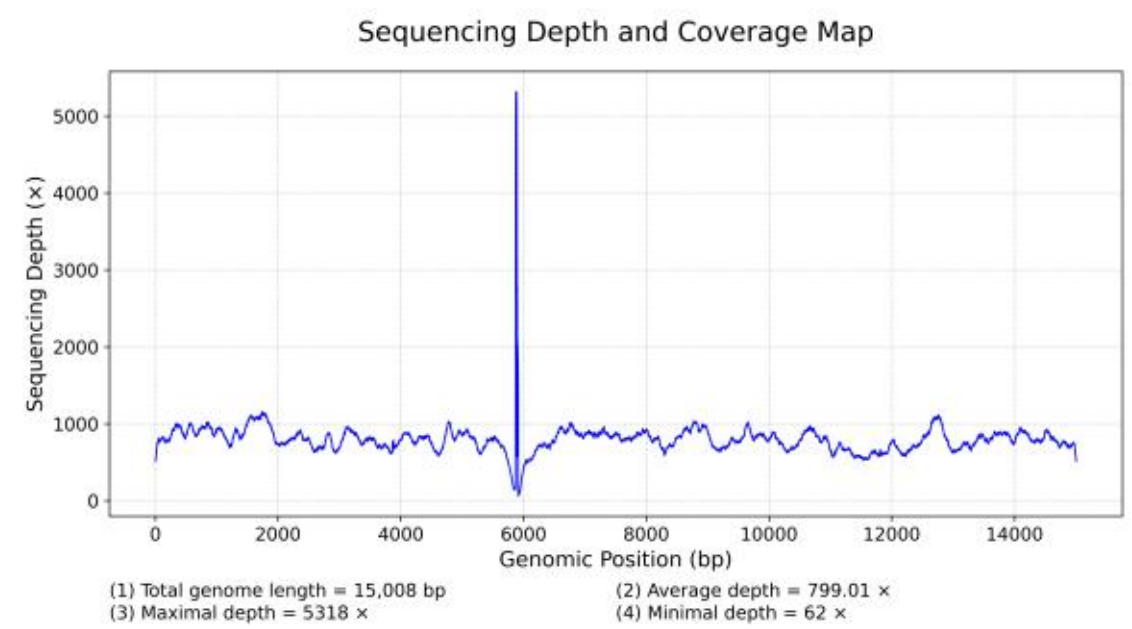

M. duodiverticulus CJJ191 L04

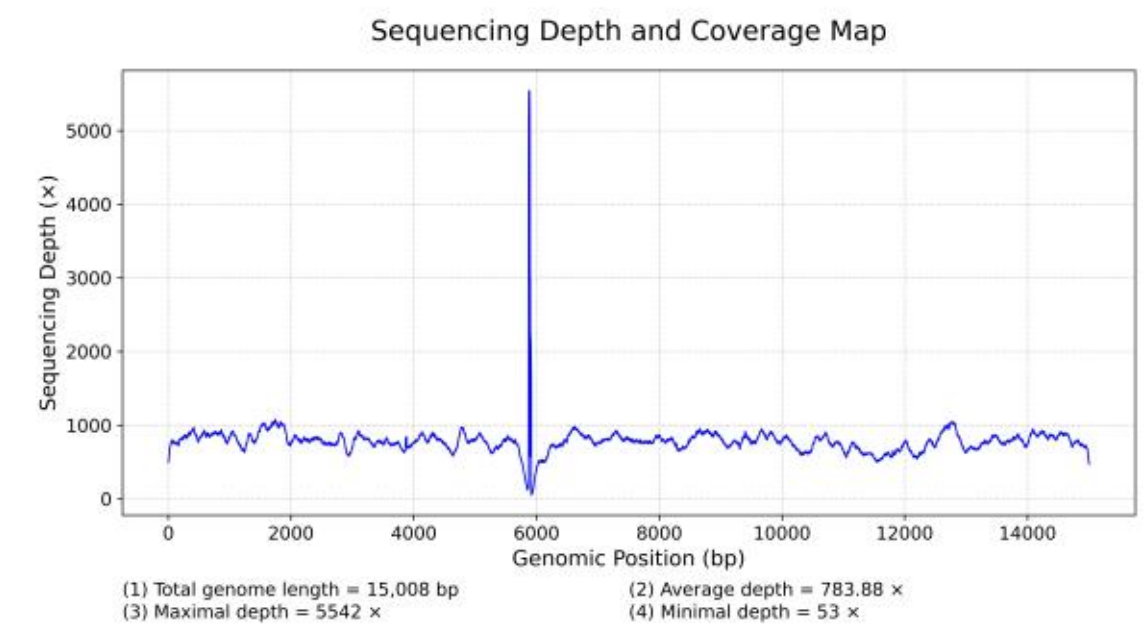

M. fokulen L02

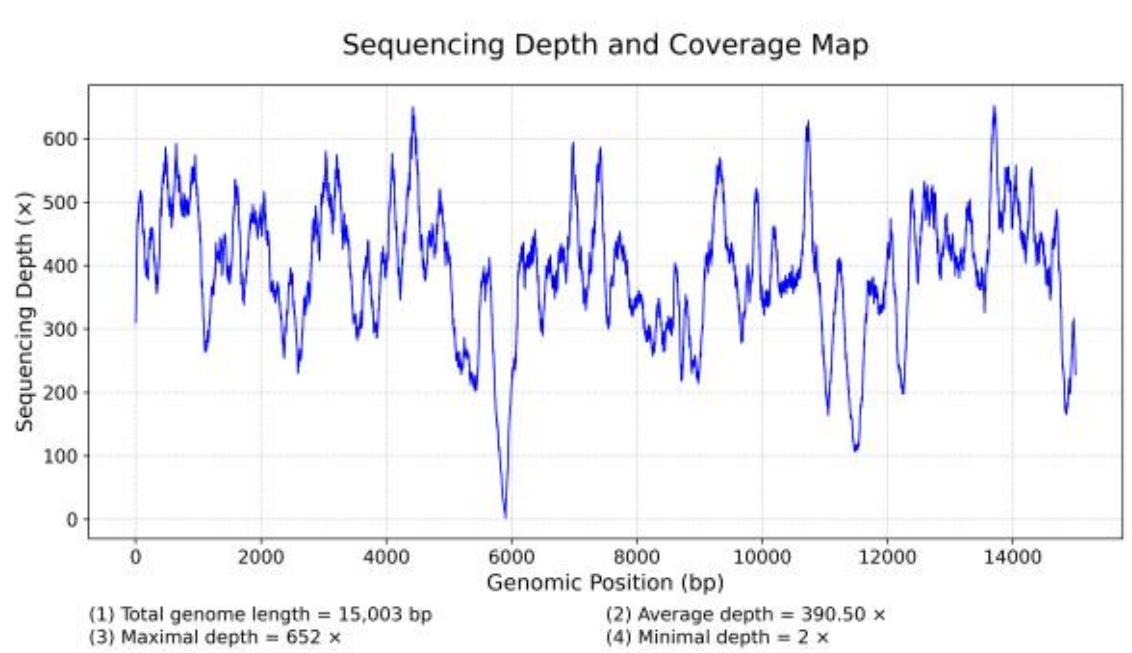

M. fokulen L03

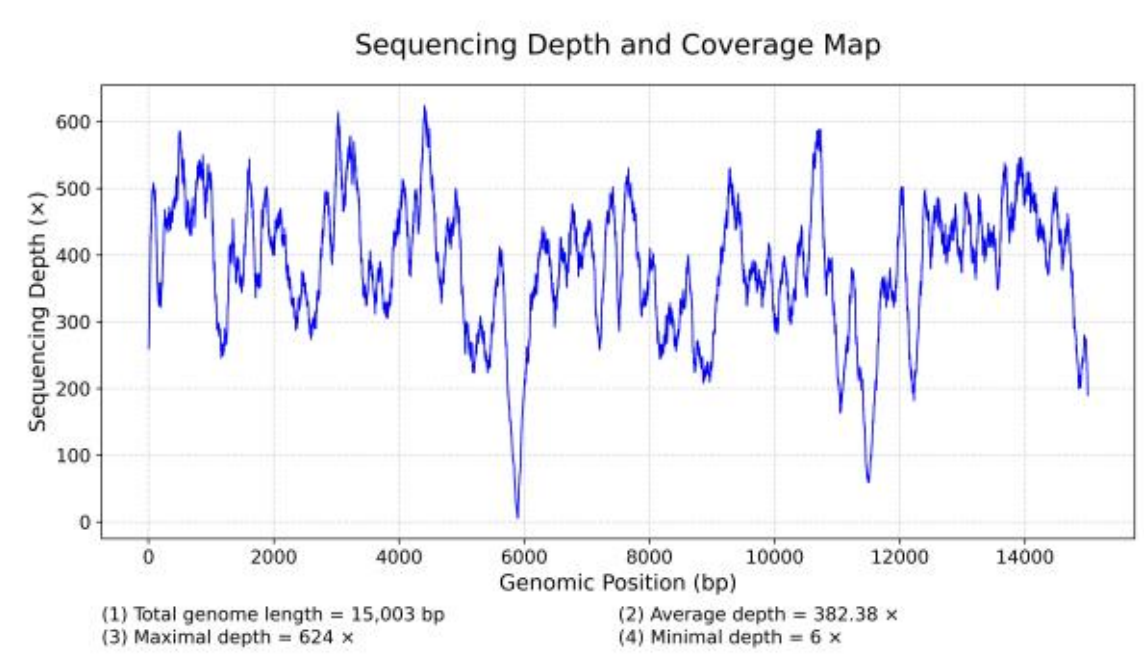

M. fokulen L04

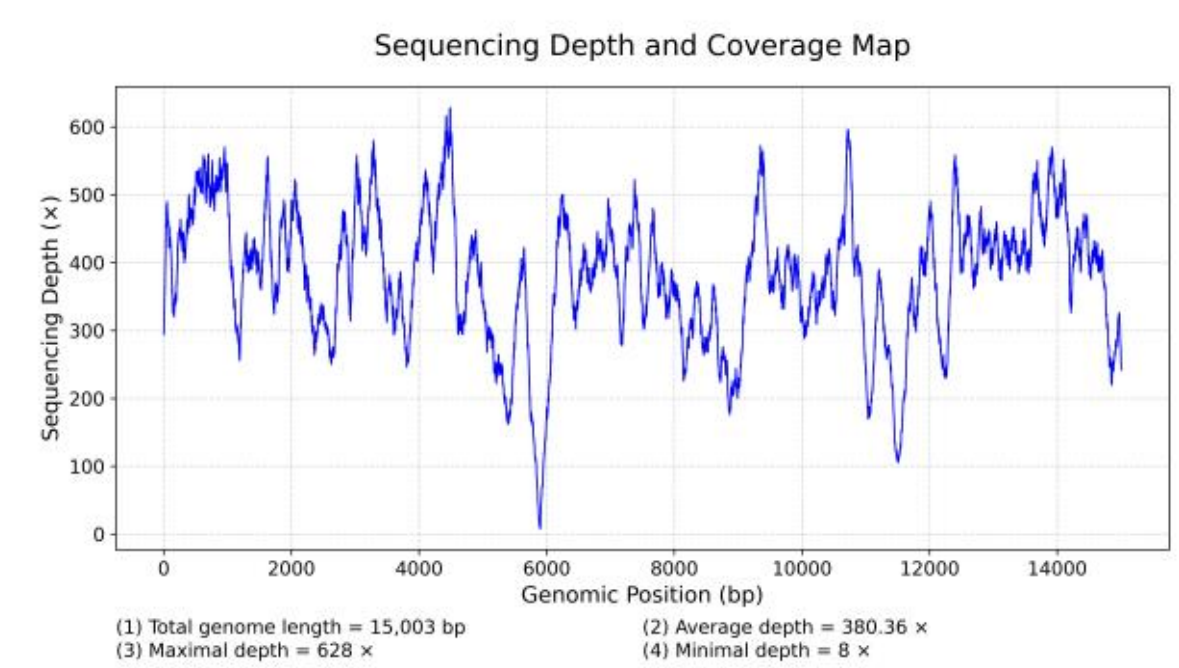

M. digitalisdiverticulus L02

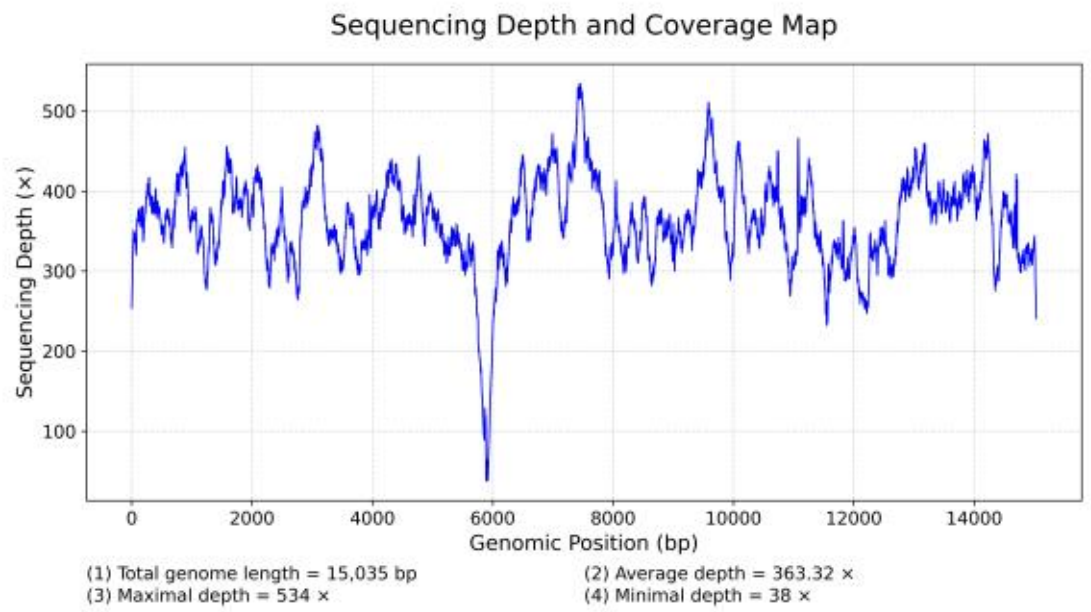

M. digitalisdiverticulus L03

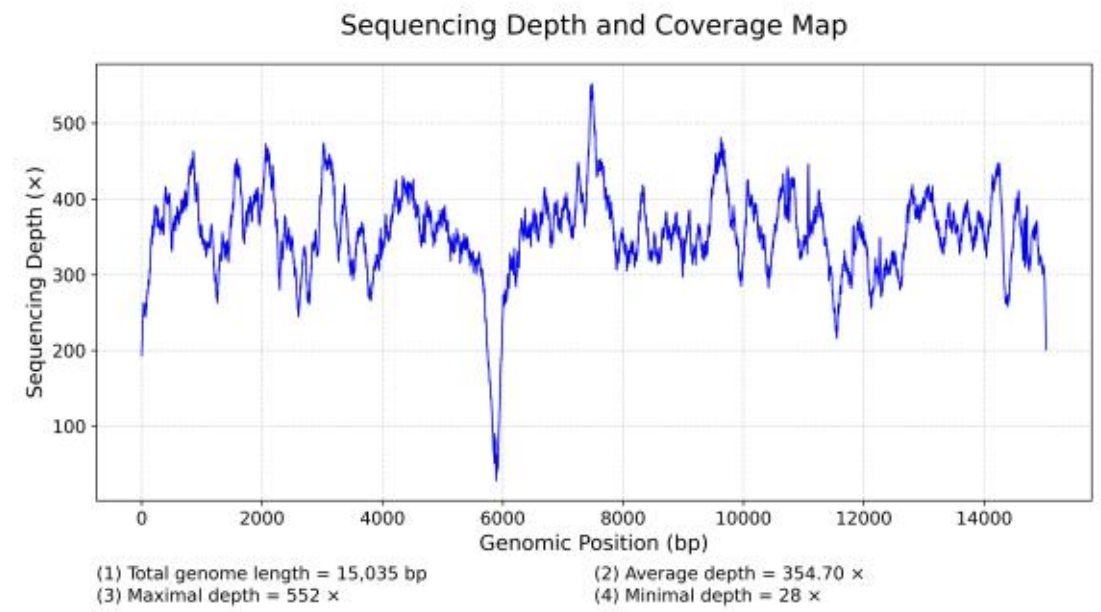

M. digitalisdiverticulus L04

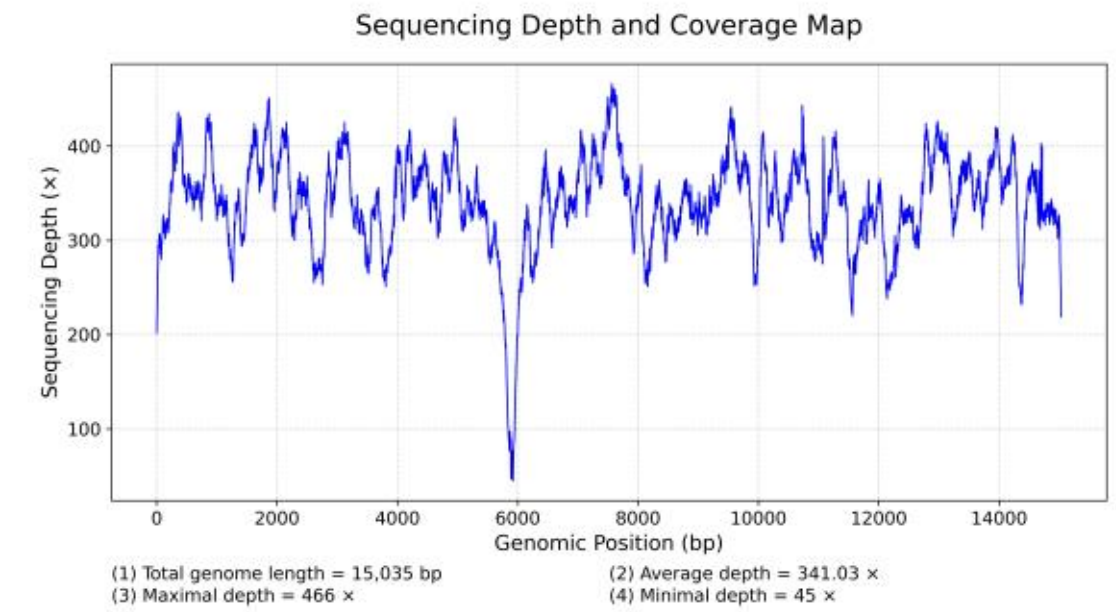

M. ngulen L02

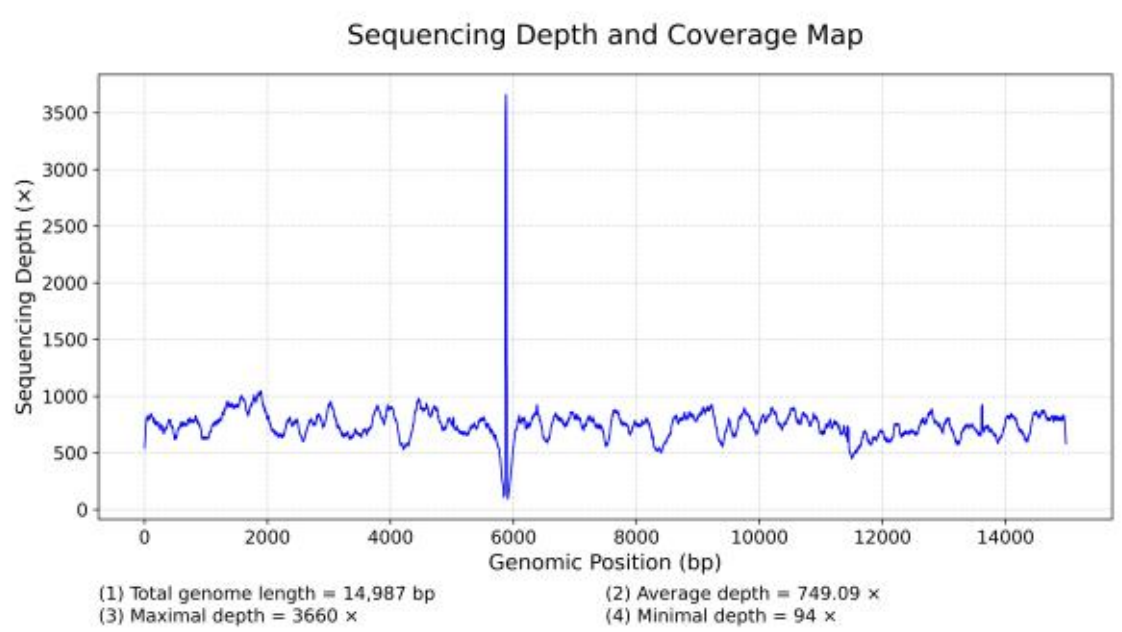

M. ngulen L03

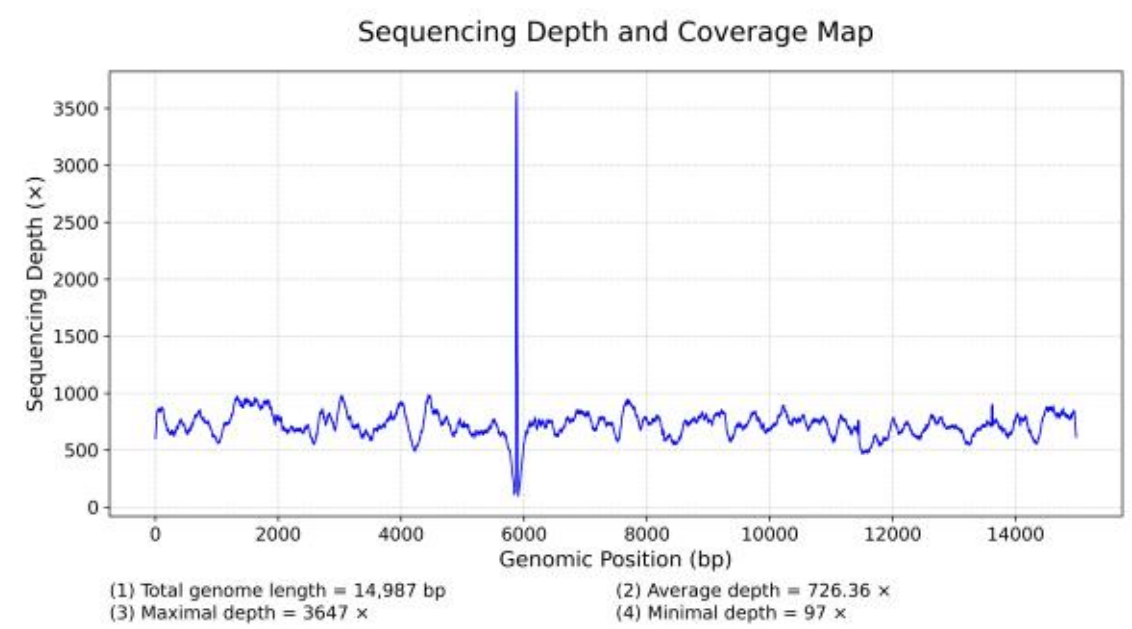

M. ngulen L04

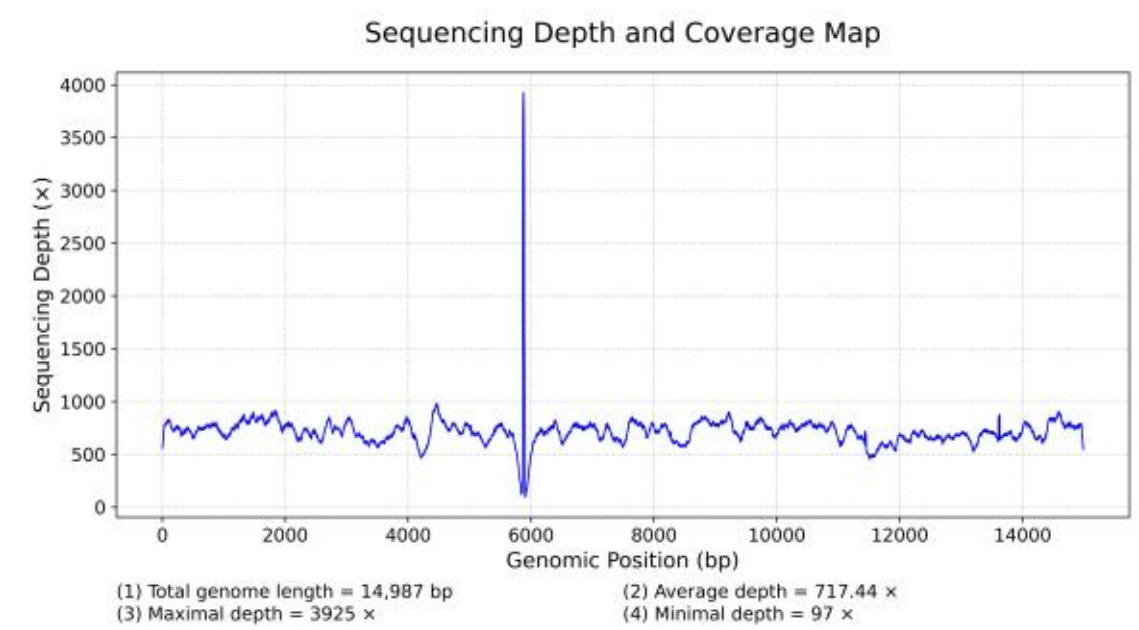

M. monodiverticulus L02

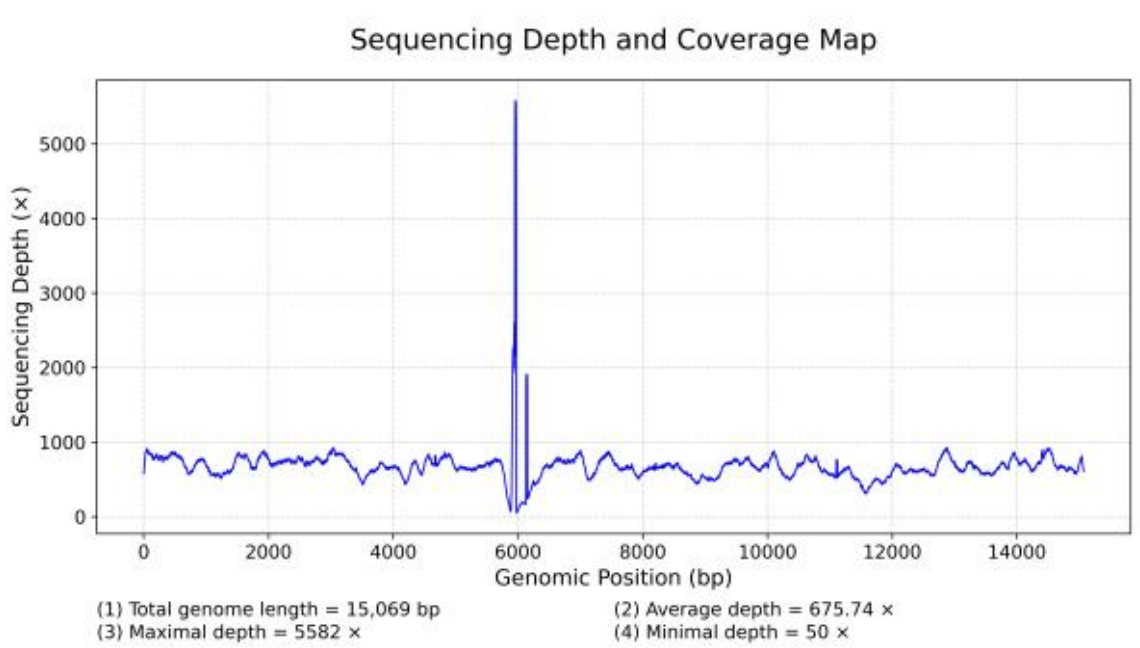

M. monodiverticulus L03

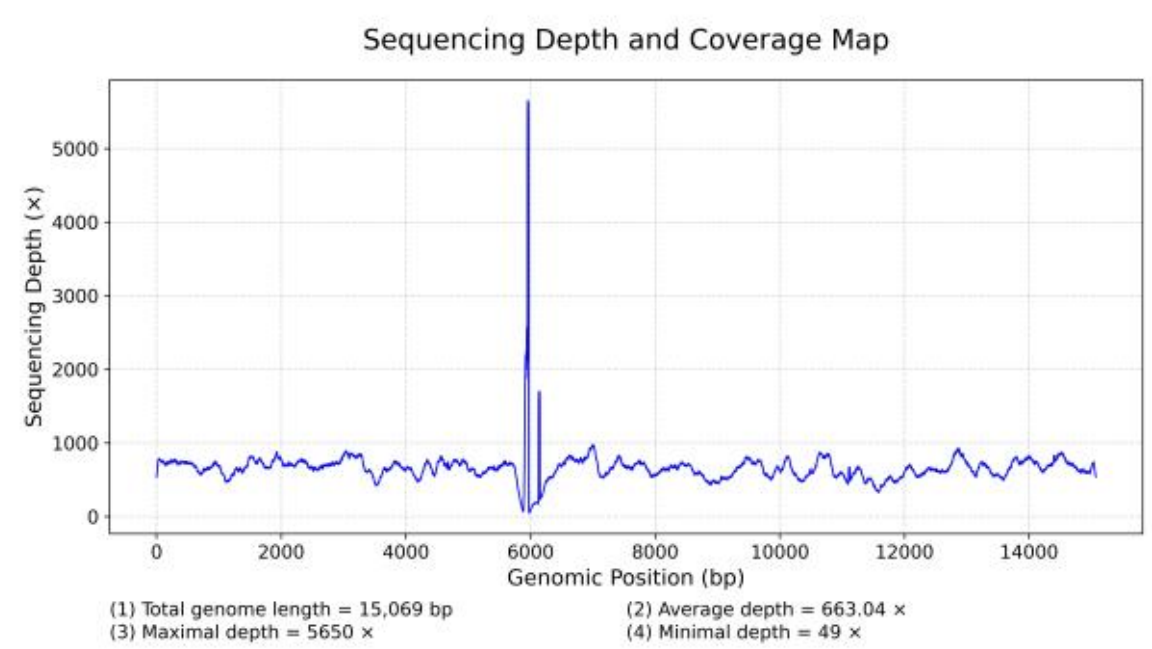

M. monodiverticulus L04

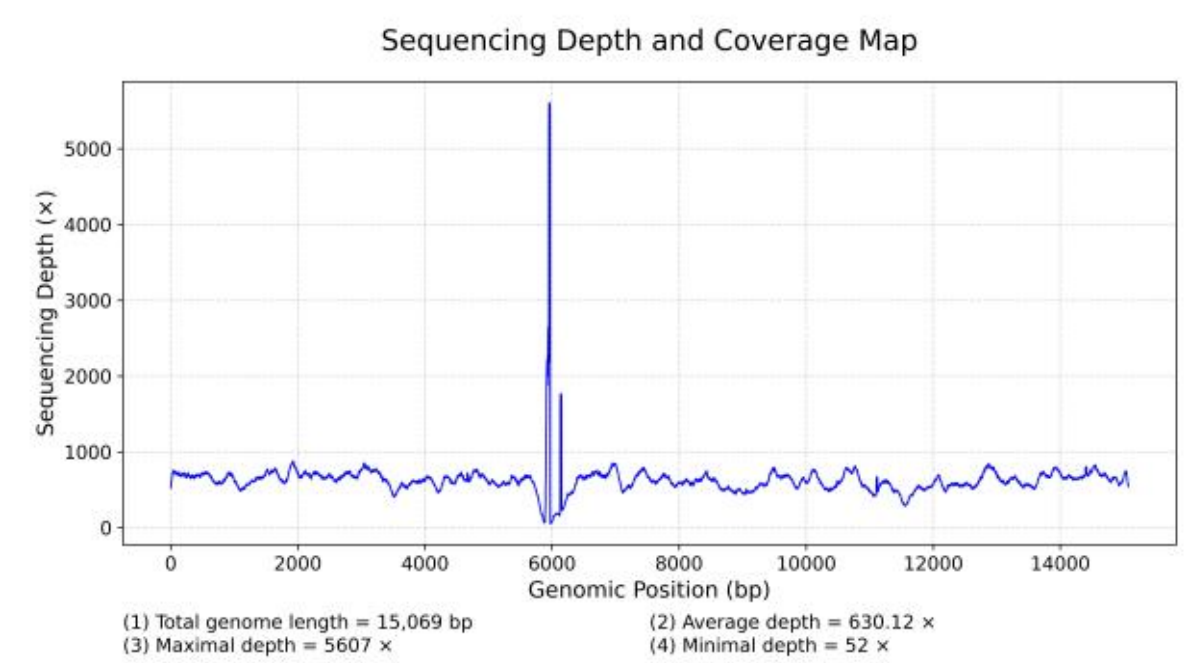

M. parvdiverticulus L02

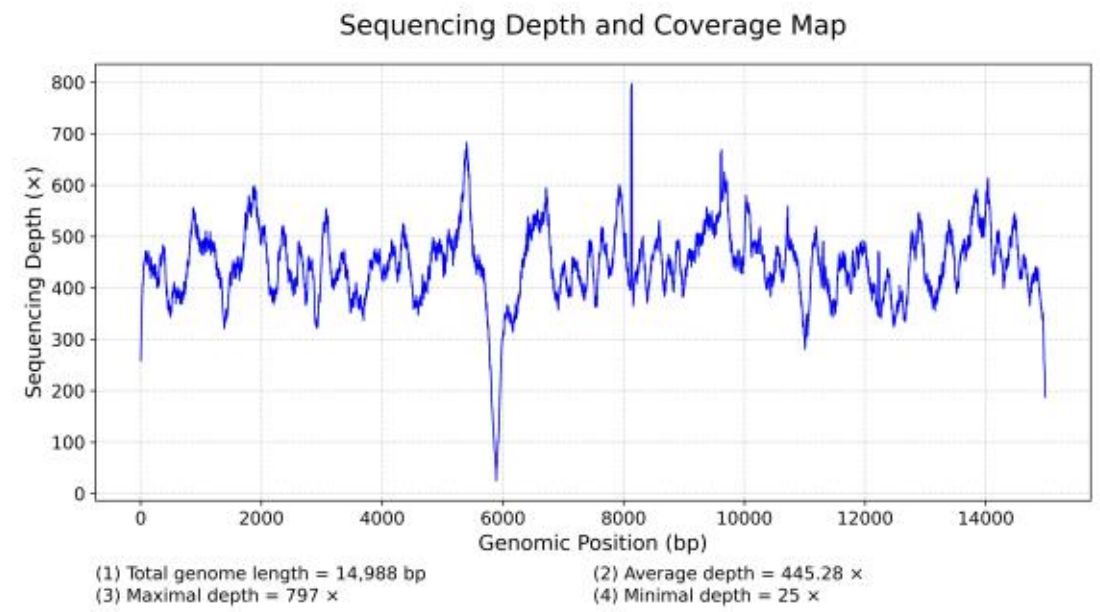

M. parvdiverticulus L03

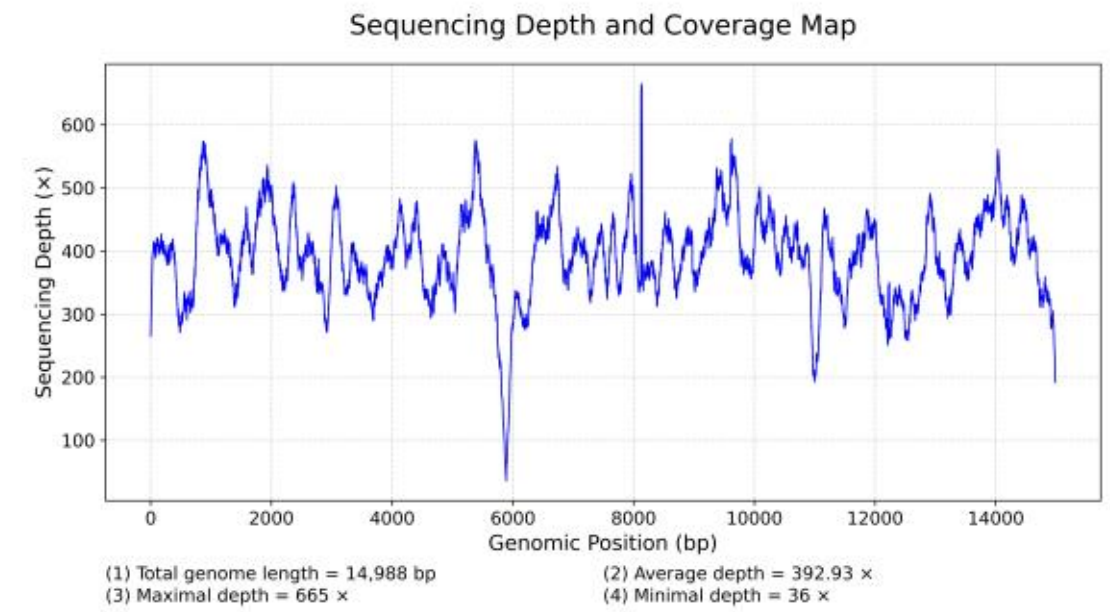

M. parvdiverticulus L04

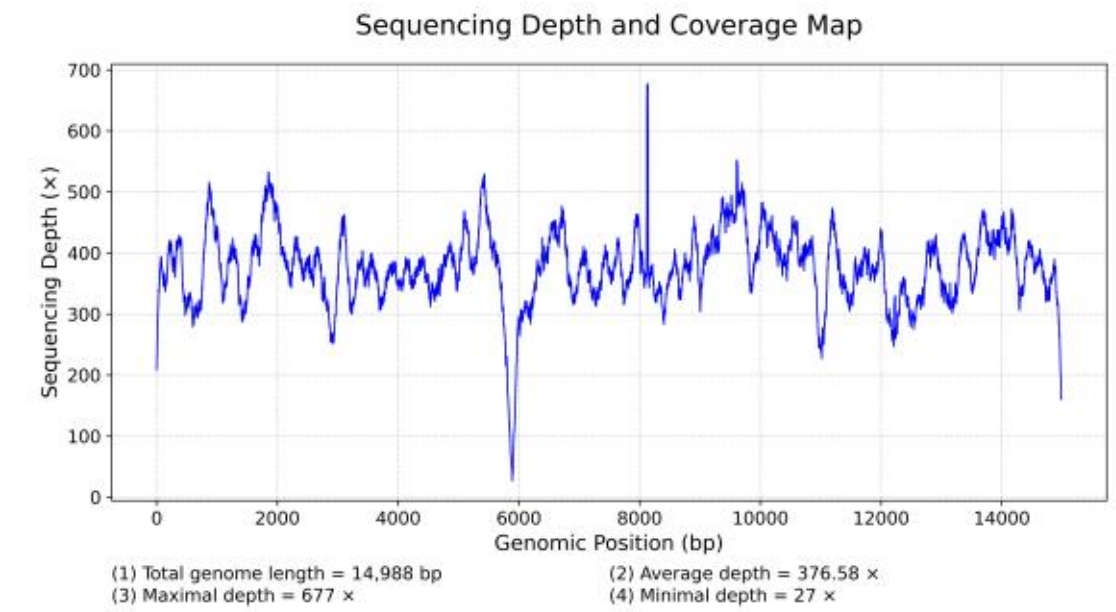

M. zhenggulen L02

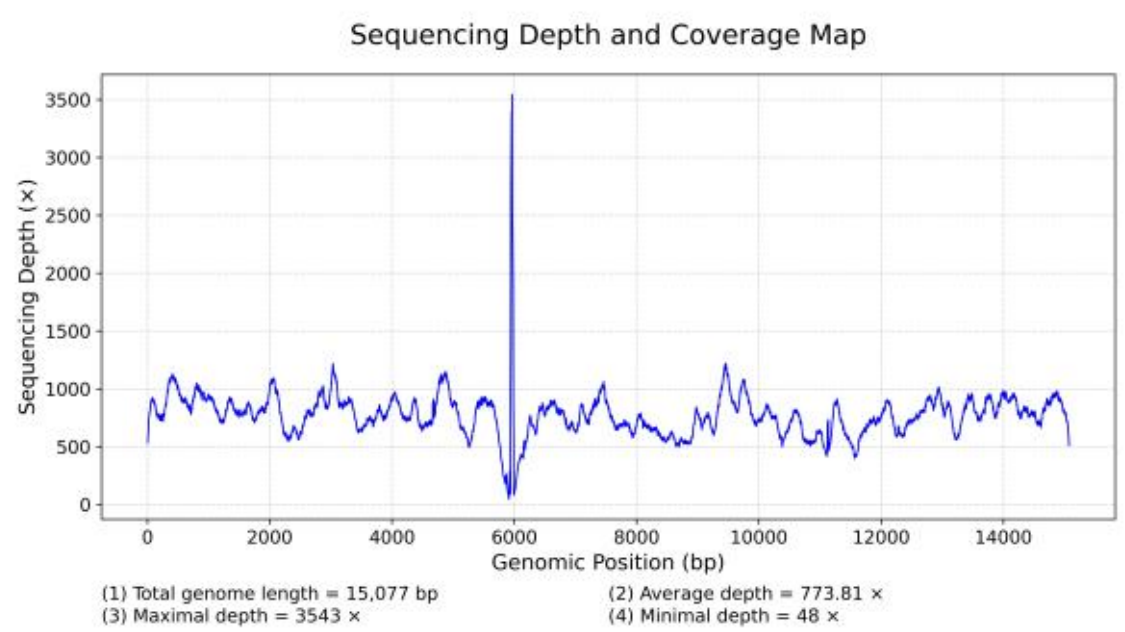

M. zhenggulen L03

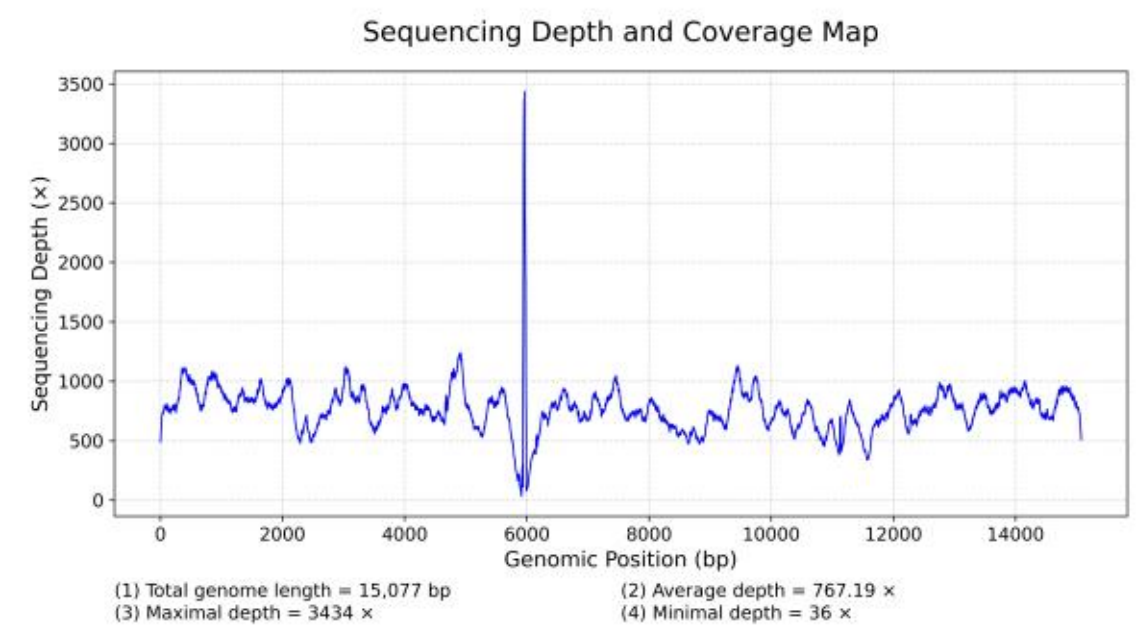

M. zhenggulen L04

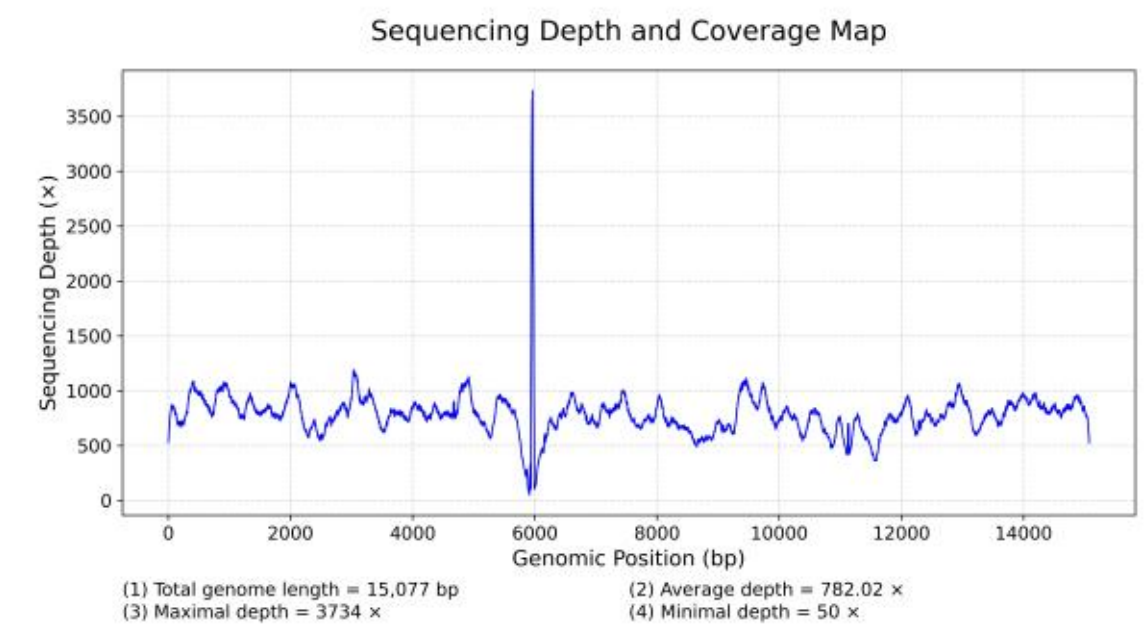

Mesenchytraeus sp. L02

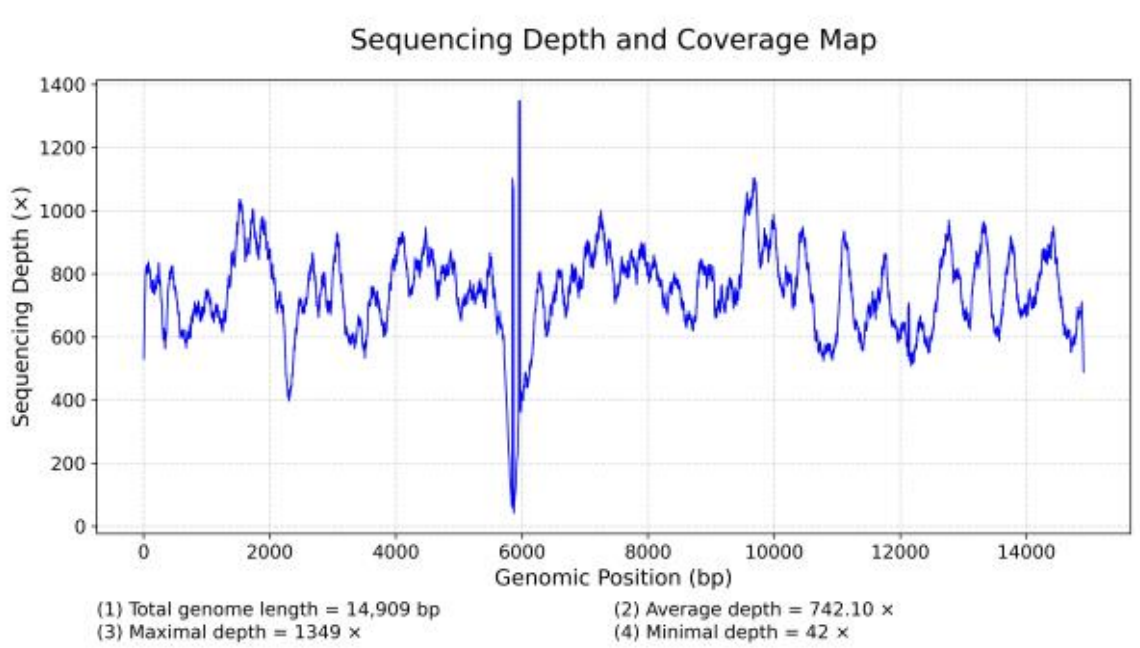

Mesenchytraeus sp. L03

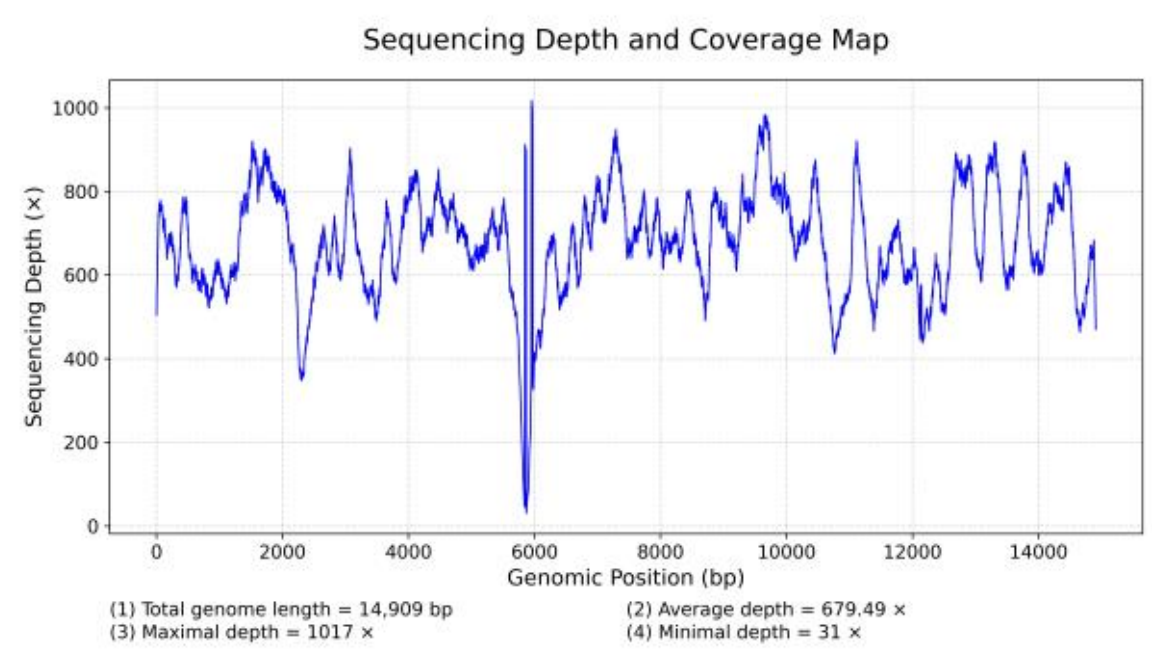

Mesenchytraeus sp. L04

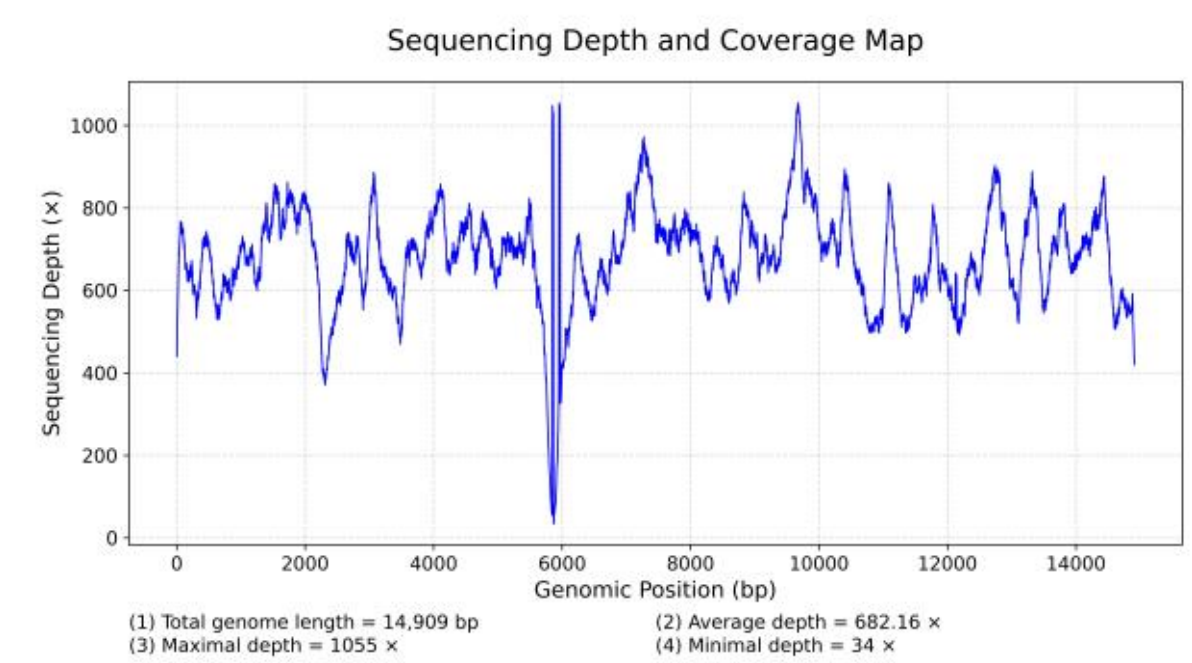

M. infradiverticulus L02

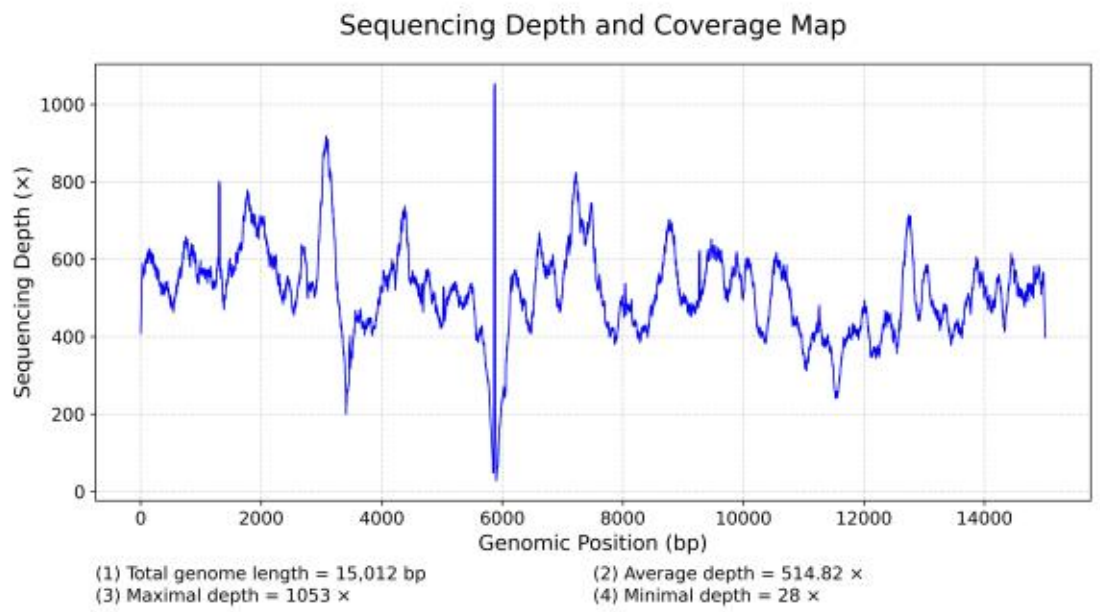

M. infradiverticulus L03

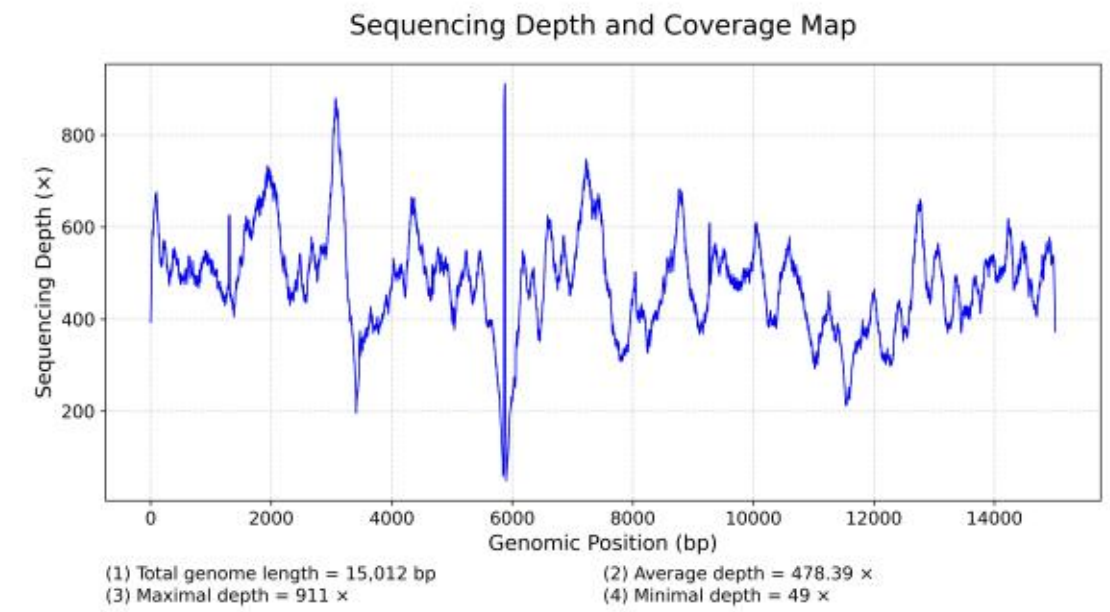

M. infradiverticulus L04

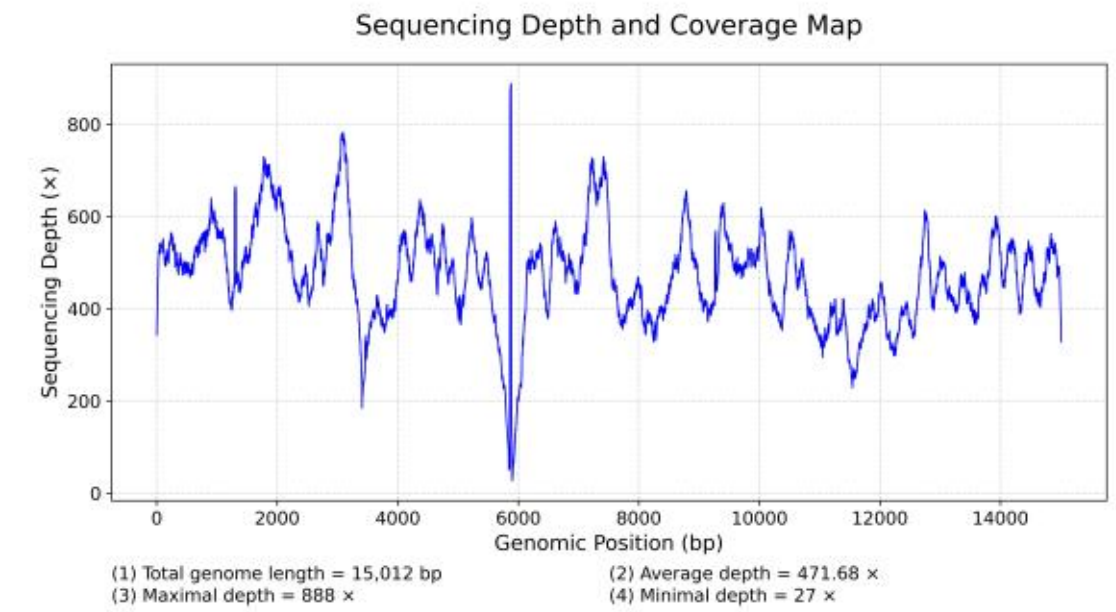

M. anisodiverticulus L02

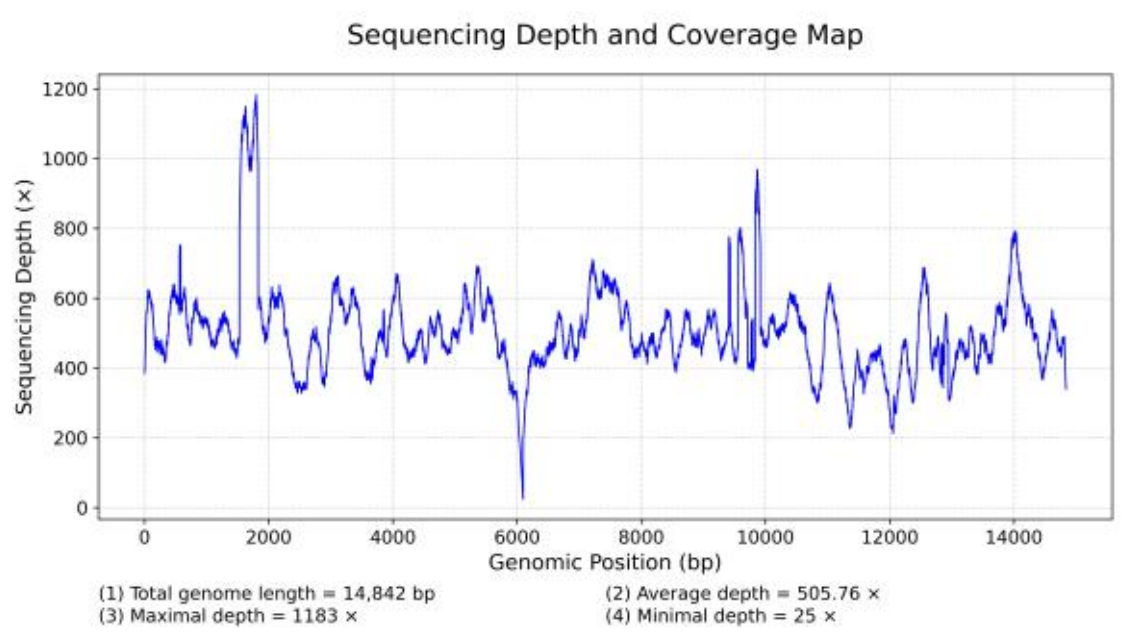

M. anisodiverticulus L03

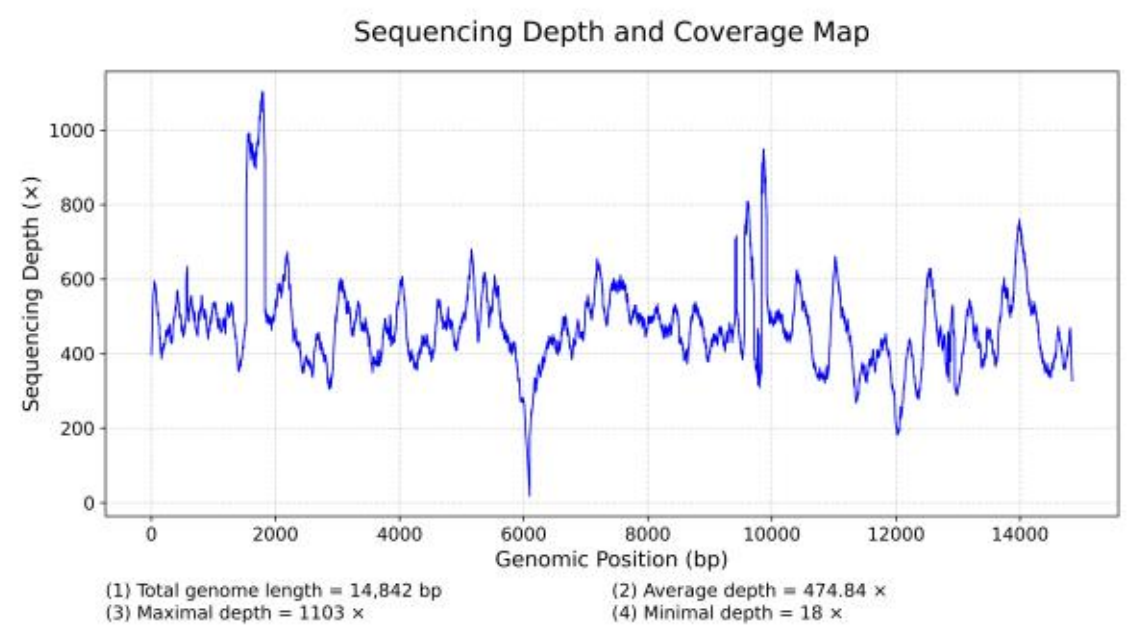

M. anisodiverticulus L04

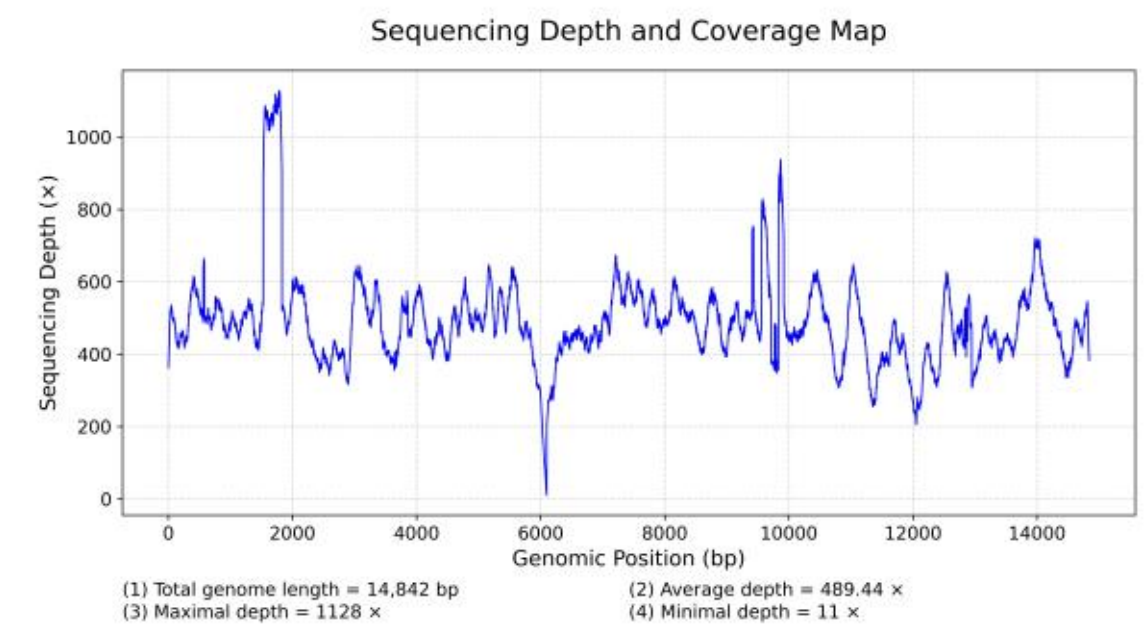

Supplement: Supplementary file 2 [file ECE3-16-e74293-s002.pdf]
