# Supplementary material for: De Novo Assembly and Comparative Analysis of the Complete Mitochondrial Genome of Mesenchytraeus (Annelida, Enchytraeidae)
Source: Ecol Evol. 2026 Sep 4;16(9):e74293. doi: 10.1002/ece3.74293 (PMC13543304; doi:10.1002/ece3.74293)
Supplement: Supplementary file 3 [file ECE3-16-e74293-s003.pdf]

Table S1. Organization and structure of the mitochondrial genomes of the different specimens of *Mesenchytraeus* studied included in this study

Specimen 1：CJJ6 *Mesenchytraeus gigachaetus* 707

Gene	Strand	From	To	Size(pb)	Start	Stop	Anti-codon	Intergenic bases(bp)
COX1	+	1	1537	1537	*AUG*	*U--*		0
tRNA-Asn	+	1538	1601	64			*GUU*	0
COX2	+	1603	2281	679	*AUG*	*U--*		1
tRNA-Asp	+	2282	2343	62			*GUC*	0
ATP8	+	2346	2502	157	*AUG*	*U--*		2
tRNA-Tyr	+	2503	2566	64			*GUA*	0
tRNA-Gly	+	2567	2626	60			*UCC*	0
COX3	+	2628	3405	778	*AUG*	*U--*		1
tRNA-Gln	+	3406	3474	69			*UUG*	0
ND6	+	3475	3951	477	*AUG*	*UAA*		0
CYTB	+	3944	5081	1138	*AUG*	*U--*		-8
tRNA-Trp	+	5082	5142	61			*UCA*	0
ATP6	+	5144	5839	696	*AUG*	*UAA*		1
Putative Control Region	+	5840	6336	497				0
tRNA-Arg	+	6337	6393	57			*UCG*	0
tRNA-His	+	6395	6455	61			*GUG*	1
ND5	+	6456	8190	1735	*AUG*	*U--*		0
tRNA-Phe	+	8191	8251	61			*GAA*	0
tRNA-Glu	+	8252	8313	62			*UUC*	0
tRNA-Pro	+	8314	8375	62			*UGG*	0
tRNA-Thr	+	8376	8436	61			*UGU*	0
ND4L	+	8437	8733	297	*AUG*	*UAA*		0
ND4	+	8727	10083	1357	*AUG*	*U--*		-7
tRNA-Cys	+	10084	10145	62			*GCA*	0
tRNA-Met	+	10146	10208	63			*CAU*	0
s-rRNA	+	10209	10974	766				0
tRNA-Val	+	10975	11037	63			*UAC*	0
l-rRNA	+	11038	12284	1247				0
tRNA-Leu1	+	12285	12345	61			*UAG*	0
tRNA-Ala	+	12346	12403	58			*UGC*	0
tRNA-Ser1	+	12404	12470	67			*UGA*	0
tRNA-Leu2	+	12471	12532	62			*UAA*	0
ND1	+	12533	13470	938	*AUG*	*UA-*		0
tRNA-Ile	+	13471	13532	62			*GAU*	0
tRNA-Lys	+	13533	13595	63			*UUU*	0
ND3	+	13596	13947	352	*AUG*	*U--*		0
tRNA-Ser2	+	13948	14011	64			*UCU*	0
ND2	+	14012	15014	1003	*AUG*	*U--*		0

Specimen 2：CJJ144 *Mesenchytraeus spermatoglomeratus* 706

Gene	Strand	From	To	Size(pb)	Start	Stop	Anti-codon	Intergenic bases(bp)
COX1	+	1	1537	1537	*AUG*	*U--*		0
tRNA-Asn	+	1538	1601	64			*GUU*	0
COX2	+	1603	2281	679	*AUG*	*U--*		1
tRNA-Asp	+	2282	2342	61			*GUC*	0
ATP8	+	2345	2501	157	*AUG*	*U--*		2
tRNA-Tyr	+	2502	2564	63			*GUA*	0
tRNA-Gly	+	2565	2624	60			*UCC*	0
COX3	+	2626	3404	779	*AUG*	*UA-*		1
tRNA-Gln	+	3408	3476	69			*UUG*	3
ND6	+	3478	3954	477	*AUG*	*UAA*		1
CYTB	+	3947	5084	1138	*AUG*	*U--*		-8
tRNA-Trp	+	5085	5145	61			*UCA*	0
ATP6	+	5147	5842	696	*AUG*	*UAA*		1
Putative Control Region	+	5843	6320	478				0
tRNA-Arg	+	6321	6378	58			*UCG*	0
tRNA-His	+	6380	6440	61			*GUG*	1
ND5	+	6441	8175	1735	*AUG*	*U--*		0
tRNA-Phe	+	8176	8235	60			*GAA*	0
tRNA-Glu	+	8236	8299	64			*UUC*	0
tRNA-Pro	+	8300	8361	62			*UGG*	0
tRNA-Thr	+	8362	8426	65			*UGU*	0
ND4L	+	8427	8723	297	*AUG*	*UAA*		0
ND4	+	8717	10073	1357	*AUG*	*U--*		-7
tRNA-Cys	+	10074	10135	62			*GCA*	0
tRNA-Met	+	10136	10197	62			*CAU*	0
s-rRNA	+	10198	10961	764				0
tRNA-Val	+	10962	11025	64			*UAC*	0
l-rRNA	+	11026	12271	1246				0
tRNA-Leu1	+	12272	12333	62			*UAG*	0
tRNA-Ala	+	12334	12391	58			*UGC*	0
tRNA-Ser1	+	12392	12458	67			*UGA*	0
tRNA-Leu2	+	12459	12520	62			*UAA*	0
ND1	+	12521	13457	937	*AUG*	*U--*		0
tRNA-Ile	+	13458	13520	63			*GAU*	0
tRNA-Lys	+	13521	13583	63			*UUU*	0
ND3	+	13584	13935	352	*AUG*	*U--*		0
tRNA-Ser2	+	13936	13999	65			*UCU*	0
ND2	+	14000	15002	1003	*AUG*	*U--*		0

Specimen 3：CJJ147 *Mesenchytraeus similigigachaetus* 684

Gene	Strand	From	To	Size(pb)	Start	Stop	Anti-codon	Intergenic bases(bp)
COX1	+	1	1537	1537	*AUG*	*U--*		0
tRNA-Asn	+	1538	1601	64			*GUU*	0
COX2	+	1603	2281	679	*AUG*	*U--*		1
tRNA-Asp	+	2282	2342	61			*GUC*	0
ATP8	+	2345	2501	157	*AUG*	*U--*		2
tRNA-Tyr	+	2502	2570	69			*GUA*	0
tRNA-Gly	+	2571	2630	60			*UCC*	0
COX3	+	2632	3411	780	*AUG*	*UAA*		1
tRNA-Gln	+	3415	3483	69			*UUG*	3
ND6	+	3485	3961	477	*AUG*	*UAA*		1
CYTB	+	3954	5091	1138	*AUG*	*U--*		-8
tRNA-Trp	+	5092	5152	61			*UCA*	0
ATP6	+	5154	5849	696	*AUG*	*UAA*		1
Putative Control Region	+	5850	6337	488				0
tRNA-Arg	+	6338	6398	61			*UCG*	0
tRNA-His	+	6400	6460	61			*GUG*	1
ND5	+	6461	8192	1732	*AUG*	*U--*		0
tRNA-Phe	+	8193	8252	60			*GAA*	0
tRNA-Glu	+	8253	8312	60			*UUC*	0
tRNA-Pro	+	8316	8378	63			*UGG*	3
tRNA-Thr	+	8379	8440	62			*UGU*	0
ND4L	+	8441	8737	297	*AUG*	*UAA*		0
ND4	+	8731	10087	1357	*AUG*	*U--*		-7
tRNA-Cys	+	10089	10151	63			*GCA*	1
tRNA-Met	+	10152	10213	62			*CAU*	0
s-rRNA	+	10214	10995	782				0
tRNA-Val	+	10996	11060	65			*UAC*	0
l-rRNA	+	11061	12314	1254				0
tRNA-Leu1	+	12315	12376	62			*UAG*	0
tRNA-Ala	+	12377	12435	59			*UGC*	0
tRNA-Ser1	+	12436	12502	67			*UGA*	0
tRNA-Leu2	+	12503	12565	63			*UAA*	0
ND1	+	12566	13504	939	*AUG*	*UAA*		0
tRNA-Ile	+	13505	13567	63			*GAU*	0
tRNA-Lys	+	13568	13631	64			*UUU*	0
ND3	+	13632	13983	352	*AUG*	*U--*		0
tRNA-Ser2	+	13984	14047	64			*UCU*	0
ND2	+	14048	15050	1003	*AUG*	*U--*		0

Specimen 4：CJJ190 *Mesenchytraeus duodiverticulus* 688

Gene	Strand	From	To	Size(pb)	Start	Stop	Anti-codon	Intergenic bases(bp)
COX1	+	1	1537	1537	*AUG*	*U--*		0
tRNA-Asn	+	1538	1602	65			*GUU*	0
COX2	+	1604	2282	679	*AUG*	*U--*		1
tRNA-Asp	+	2283	2346	64			*GUC*	0
ATP8	+	2349	2505	157	*AUG*	*U--*		2
tRNA-Tyr	+	2506	2567	62			*GUA*	0
tRNA-Gly	+	2568	2627	60			*UCC*	0
COX3	+	2629	3408	780	*AUG*	*UAA*		1
tRNA-Gln	+	3409	3477	69			*UUG*	0
ND6	+	3479	3955	477	*AUG*	*UAA*		1
CYTB	+	3948	5085	1138	*AUG*	*U--*		-8
tRNA-Trp	+	5086	5146	61			*UCA*	0
ATP6	+	5148	5843	696	*AUG*	*UAA*		1
Putative Control Region	+	5844	6285	442				0
tRNA-Arg	+	6286	6344	59			*UCG*	0
tRNA-His	+	6346	6406	61			*GUG*	1
ND5	+	6407	8141	1735	*AUG*	*U--*		0
tRNA-Phe	+	8143	8204	62			*GAA*	1
tRNA-Glu	+	8206	8270	65			*UUC*	1
tRNA-Pro	+	8272	8334	63			*UGG*	1
tRNA-Thr	+	8335	8399	65			*UGU*	0
ND4L	+	8400	8696	297	*AUG*	*UAA*		0
ND4	+	8690	10046	1357	*AUG*	*U--*		-7
tRNA-Cys	+	10047	10108	62			*GCA*	0
tRNA-Met	+	10109	10170	62			*CAU*	0
s-rRNA	+	10171	10936	766				0
tRNA-Val	+	10937	10999	63			*UAC*	0
l-rRNA	+	11000	12248	1249				0
tRNA-Leu1	+	12249	12310	62			*UAG*	0
tRNA-Ala	+	12311	12368	58			*UGC*	0
tRNA-Ser1	+	12369	12434	66			*UGA*	0
tRNA-Leu2	+	12435	12497	63			*UAA*	0
ND1	+	12498	13435	938	*AUG*	*UA-*		0
tRNA-Ile	+	13436	13498	63			*GAU*	0
tRNA-Lys	+	13499	13561	63			*UUU*	0
ND3	+	13562	13914	353	*AUG*	*UA-*		0
tRNA-Ser2	+	13915	13978	64			*UCU*	0
ND2	+	13979	14981	1003	*AUG*	*U--*		0

Specimen 5：CJJ191 *Mesenchytraeus duodiverticulus* 687

Gene	Strand	From	To	Size(pb)	Start	Stop	Anti-codon	Intergenic bases(bp)
COX1	+	1	1537	1537	*AUG*	*U--*		0
tRNA-Asn	+	1538	1602	65			*GUU*	0
COX2	+	1604	2282	679	*AUG*	*U--*		1
tRNA-Asp	+	2283	2346	64			*GUC*	0
ATP8	+	2349	2505	157	*AUG*	*U--*		2
tRNA-Tyr	+	2506	2567	62			*GUA*	0
tRNA-Gly	+	2568	2627	60			*UCC*	0
COX3	+	2629	3408	780	*AUG*	*UAA*		1
tRNA-Gln	+	3409	3477	69			*UUG*	0
ND6	+	3479	3955	477	*AUG*	*UAA*		1
CYTB	+	3948	5085	1138	*AUG*	*U--*		-8
tRNA-Trp	+	5086	5146	61			*UCA*	0
ATP6	+	5148	5843	696	*AUG*	*UAA*		1
Putative Control Region	+	5844	6312	469				0
tRNA-Arg	+	6313	6371	59			*UCG*	0
tRNA-His	+	6373	6433	61			*GUG*	1
ND5	+	6434	8168	1735	*AUG*	*U--*		0
tRNA-Phe	+	8170	8231	62			*GAA*	1
tRNA-Glu	+	8233	8297	65			*UUC*	1
tRNA-Pro	+	8299	8361	63			*UGG*	1
tRNA-Thr	+	8362	8426	65			*UGU*	0
ND4L	+	8427	8723	297	*AUG*	*UAA*		0
ND4	+	8717	10073	1357	*AUG*	*U--*		-7
tRNA-Cys	+	10074	10135	62			*GCA*	0
tRNA-Met	+	10136	10197	62			*CAU*	0
s-rRNA	+	10198	10963	766				0
tRNA-Val	+	10964	11026	63			*UAC*	0
l-rRNA	+	11027	12275	1249				0
tRNA-Leu1	+	12276	12337	62			*UAG*	0
tRNA-Ala	+	12338	12395	58			*UGC*	0
tRNA-Ser1	+	12396	12461	66			*UGA*	0
tRNA-Leu2	+	12462	12524	63			*UAA*	0
ND1	+	12525	13462	938	*AUG*	*UA-*		0
tRNA-Ile	+	13463	13525	63			*GAU*	0
tRNA-Lys	+	13526	13588	63			*UUU*	0
ND3	+	13589	13941	353	*AUG*	*UA-*		0
tRNA-Ser2	+	13942	14005	64			*UCU*	0
ND2	+	14006	15008	1003	*AUG*	*U--*		0

Specimen 6：CJJ194 *Mesenchytraeus fokulen* 403

Gene	Strand	From	To	Size(pb)	Start	Stop	Anti-codon	Intergenic bases(bp)
COX1	+	1	1537	1537	*AUG*	*U--*		0
tRNA-Asn	+	1538	1604	67			*GUU*	0
COX2	+	1606	2284	679	*AUG*	*U--*		1
tRNA-Asp	+	2285	2345	61			*GUC*	0
ATP8	+	2348	2504	157	*AUG*	*U--*		2
tRNA-Tyr	+	2505	2566	62			*GUA*	0
tRNA-Gly	+	2567	2626	60			*UCC*	0
COX3	+	2628	3407	780	*AUG*	*UAA*		1
tRNA-Gln	+	3408	3476	69			*UUG*	0
ND6	+	3478	3954	477	*AUG*	*UAA*		1
CYTB	+	3947	5084	1138	*AUG*	*U--*		-8
tRNA-Trp	+	5085	5145	61			*UCA*	0
ATP6	+	5147	5842	696	*AUG*	*UAG*		1
Putative Control Region	+	5843	6308	466				0
tRNA-Arg	+	6309	6366	58			*UCG*	0
tRNA-His	+	6368	6427	60			*GUG*	1
ND5	+	6428	8162	1735	*AUG*	*U--*		0
tRNA-Phe	+	8163	8225	63			*GAA*	0
tRNA-Glu	+	8227	8289	63			*UUC*	1
tRNA-Pro	+	8290	8351	62			*UGG*	0
tRNA-Thr	+	8352	8413	62			*UGU*	0
ND4L	+	8414	8710	297	*AUG*	*UAA*		0
ND4	+	8704	10060	1357	*AUG*	*U--*		-7
tRNA-Cys	+	10061	10122	62			*GCA*	0
tRNA-Met	+	10123	10184	62			*CAU*	0
s-rRNA	+	10185	10951	767				0
tRNA-Val	+	10952	11013	62			*UAC*	0
l-rRNA	+	11014	12260	1247				0
tRNA-Leu1	+	12261	12322	62			*UAG*	0
tRNA-Ala	+	12323	12380	58			*UGC*	0
tRNA-Ser1	+	12381	12446	66			*UGA*	0
tRNA-Leu2	+	12447	12510	64			*UAA*	0
ND1	+	12511	13448	938	*AUG*	*UA-*		0
tRNA-Ile	+	13449	13513	65			*GAU*	0
tRNA-Lys	+	13514	13576	63			*UUU*	0
ND3	+	13577	13930	354	*AUG*	*UAA*		0
tRNA-Ser2	+	13931	13994	64			*UCU*	0
ND2	+	13995	15003	1009	*AUG*	*U--*		0

Specimen 7：CJJ201 *Mesenchytraeus digitalisdiverticulus* 686

Gene	Strand	From	To	Size(pb)	Start	Stop	Anti-codon	Intergenic bases(bp)
COX1	+	1	1537	1537	*AUG*	*U--*		0
tRNA-Asn	+	1538	1601	64			*GUU*	0
COX2	+	1603	2281	679	*AUG*	*U--*		1
tRNA-Asp	+	2282	2344	63			*GUC*	0
ATP8	+	2347	2503	157	*AUG*	*U--*		2
tRNA-Tyr	+	2504	2577	64			*GUA*	0
tRNA-Gly	+	2578	2638	61			*UCC*	0
COX3	+	2640	3419	780	*AUG*	*UAA*		1
tRNA-Gln	+	3423	3491	69			*UUG*	3
ND6	+	3493	3969	477	*AUG*	*UAA*		1
CYTB	+	3962	5099	1138	*AUG*	*U--*		-8
tRNA-Trp	+	5100	5160	61			*UCA*	0
ATP6	+	5162	5869	708	*AUG*	*UAG*		1
Putative Control Region	+	5870	6312	443				0
tRNA-Arg	+	6313	6374	62			*UCG*	0
tRNA-His	+	6376	6437	62			*GUG*	1
ND5	+	6438	8169	1732	*AUG*	*U--*		0
tRNA-Phe	+	8170	8232	63			*GAA*	0
tRNA-Glu	+	8234	8295	62			*UUC*	1
tRNA-Pro	+	8300	8363	64			*UGG*	4
tRNA-Thr	+	8364	8425	62			*UGU*	0
ND4L	+	8426	8722	297	*AUG*	*UAA*		0
ND4	+	8716	10072	1357	*AUG*	*U--*		-7
tRNA-Cys	+	10074	10136	63			*GCA*	1
tRNA-Met	+	10137	10198	62			*CAU*	0
s-rRNA	+	10199	10976	778				0
tRNA-Val	+	10977	11039	63			*UAC*	0
l-rRNA	+	11040	12297	1258				0
tRNA-Leu1	+	12299	12360	62			*UAG*	1
tRNA-Ala	+	12361	12419	59			*UGC*	0
tRNA-Ser1	+	12420	12486	67			*UGA*	0
tRNA-Leu2	+	12487	12548	62			*UAA*	0
ND1	+	12549	13486	938	*AUG*	*UA-*		0
tRNA-Ile	+	13487	13552	66			*GAU*	0
tRNA-Lys	+	13553	13616	64			*UUU*	0
ND3	+	13617	13968	352	*AUG*	*U--*		0
tRNA-Ser2	+	13969	14032	64			*UCU*	0
ND2	+	14033	15035	1003	*AUG*	*U--*		0

Specimen 8：CJJ207 *Mesenchytraeus ngulen* 709

Gene	Strand	From	To	Size(pb)	Start	Stop	Anti-codon	Intergenic bases(bp)
COX1	+	1	1537	1537	*AUG*	*U--*		0
tRNA-Asn	+	1538	1601	64			*GUU*	0
COX2	+	1603	2281	679	*AUG*	*U--*		1
tRNA-Asp	+	2282	2342	61			*GUC*	0
ATP8	+	2345	2501	157	*AUG*	*U--*		2
tRNA-Tyr	+	2502	2563	62			*GUA*	0
tRNA-Gly	+	2564	2623	60			*UCC*	0
COX3	+	2625	3404	780	*AUG*	*UAA*		1
tRNA-Gln	+	3405	3473	69			*UUG*	0
ND6	+	3475	3951	477	*AUG*	*UAA*		1
CYTB	+	3944	5081	1138	*AUG*	*U--*		-8
tRNA-Trp	+	5082	5142	61			*UCA*	0
ATP6	+	5144	5839	696	*AUG*	*UAA*		1
Putative Control Region	+	5840	6300	461				0
tRNA-Arg	+	6301	6358	58			*UCG*	0
tRNA-His	+	6360	6419	60			*GUG*	1
ND5	+	6420	8154	1735	*AUG*	*U--*		0
tRNA-Phe	+	8155	8217	63			*GAA*	0
tRNA-Glu	+	8219	8284	66			*UUC*	1
tRNA-Pro	+	8285	8346	62			*UGG*	0
tRNA-Thr	+	8347	8407	61			*UGU*	0
ND4L	+	8408	8704	297	*AUG*	*UAA*		0
ND4	+	8698	10054	1357	*AUG*	*U--*		-7
tRNA-Cys	+	10055	10116	62			*GCA*	0
tRNA-Met	+	10117	10178	62			*CAU*	0
s-rRNA	+	10179	10940	762				0
tRNA-Val	+	10941	11002	62			*UAC*	0
l-rRNA	+	11003	12250	1248				0
tRNA-Leu1	+	12251	12313	63			*UAG*	0
tRNA-Ala	+	12314	12371	58			*UGC*	0
tRNA-Ser1	+	12372	12437	66			*UGA*	0
tRNA-Leu2	+	12438	12499	62			*UAA*	0
ND1	+	12500	13437	938	*AUG*	*UA-*		0
tRNA-Ile	+	13438	13501	64			*GAU*	0
tRNA-Lys	+	13502	13564	63			*UUU*	0
ND3	+	13565	13916	352	*AUG*	*U--*		0
tRNA-Ser2	+	13917	13981	65			*UCU*	0
ND2	+	13982	14987	1006	*AUG*	*U--*		0

Specimen 9：CJJ216 *Mesenchytraeus monodiverticulus* 710

Gene	Strand	From	To	Size(pb)	Start	Stop	Anti-codon	Intergenic bases(bp)
COX1	+	1	1537	1537	*AUG*	*U--*		0
tRNA-Asn	+	1538	1605	68			*GUU*	0
COX2	+	1607	2285	679	*AUG*	*U--*		1
tRNA-Asp	+	2286	2346	61			*GUC*	0
ATP8	+	2349	2505	157	*AUG*	*U--*		2
tRNA-Tyr	+	2506	2567	62			*GUA*	0
tRNA-Gly	+	2568	2627	60			*UCC*	0
COX3	+	2629	3408	780	*AUG*	*UAA*		1
tRNA-Gln	+	3409	3477	69			*UUG*	0
ND6	+	3479	3955	477	*AUG*	*UAA*		1
CYTB	+	3948	5085	1138	*AUG*	*U--*		-8
tRNA-Trp	+	5086	5146	61			*UCA*	0
ATP6	+	5148	5843	696	*AUG*	*UAA*		1
Putative Control Region	+	5844	6368	525				0
tRNA-Arg	+	6369	6426	58			*UCG*	0
tRNA-His	+	6428	6487	60			*GUG*	1
ND5	+	6488	8222	1735	*AUG*	*U--*		0
tRNA-Phe	+	8223	8285	63			*GAA*	0
tRNA-Glu	+	8287	8349	63			*UUC*	1
tRNA-Pro	+	8350	8411	62			*UGG*	0
tRNA-Thr	+	8412	8473	62			*UGU*	0
ND4L	+	8474	8770	297	*AUG*	*UAA*		0
ND4	+	8764	10120	1357	*AUG*	*U--*		-7
tRNA-Cys	+	10121	10182	62			*GCA*	0
tRNA-Met	+	10183	10244	62			*CAU*	0
s-rRNA	+	10245	11011	767				0
tRNA-Val	+	11012	11073	62			*UAC*	0
l-rRNA	+	11074	12322	1249				0
tRNA-Leu1	+	12323	12384	62			*UAG*	0
tRNA-Ala	+	12385	12442	58			*UGC*	0
tRNA-Ser1	+	12443	12508	66			*UGA*	0
tRNA-Leu2	+	12509	12570	62			*UAA*	0
ND1	+	12571	13508	938	*AUG*	*UA-*		0
tRNA-Ile	+	13509	13572	64			*GAU*	0
tRNA-Lys	+	13573	13637	65			*UUU*	0
ND3	+	13638	13989	352	*AUG*	*U--*		0
tRNA-Ser2	+	13990	14054	65			*UCU*	0
ND2	+	14055	15069	1015	*AUG*	*U--*		0

Specimen 10：CJJ225 *Mesenchytraeus parvdiverticulus* 685

Gene	Strand	From	To	Size(pb)	Start	Stop	Anti-codon	Intergenic bases(bp)
COX1	+	1	1537	1537	*AUG*	*U--*		0
tRNA-Asn	+	1538	1600	63			*GUU*	0
COX2	+	1602	2280	679	*AUG*	*U--*		1
tRNA-Asp	+	2281	2341	61			*GUC*	0
ATP8	+	2344	2500	157	*AUG*	*U--*		2
tRNA-Tyr	+	2501	2563	63			*GUA*	0
tRNA-Gly	+	2564	2623	60			*UCC*	0
COX3	+	2625	3404	780	*AUG*	*UAA*		1
tRNA-Gln	+	3408	3476	69			*UUG*	3
ND6	+	3478	3954	477	*AUG*	*UAA*		1
CYTB	+	3947	5084	1138	*AUG*	*U--*		-8
tRNA-Trp	+	5085	5145	61			*UCA*	0
ATP6	+	5147	5842	696	*AUG*	*UAA*		1
Putative Control Region	+	5843	6299	457				0
tRNA-Arg	+	6300	6357	58			*UCG*	0
tRNA-His	+	6359	6419	61			*GUG*	1
ND5	+	6420	8154	1735	*AUG*	*U--*		0
tRNA-Phe	+	8156	8216	61			*GAA*	1
tRNA-Glu	+	8217	8278	62			*UUC*	0
tRNA-Pro	+	8279	8340	62			*UGG*	0
tRNA-Thr	+	8341	8401	61			*UGU*	0
ND4L	+	8402	8698	297	*AUG*	*UAA*		0
ND4	+	8692	10048	1357	*AUG*	*U--*		-7
tRNA-Cys	+	10049	10110	62			*GCA*	0
tRNA-Met	+	10111	10172	62			*CAU*	0
s-rRNA	+	10173	10939	767				0
tRNA-Val	+	10940	11003	64			*UAC*	0
l-rRNA	+	11004	12256	1253				0
tRNA-Leu1	+	12257	12318	62			*UAG*	0
tRNA-Ala	+	12319	12376	58			*UGC*	0
tRNA-Ser1	+	12377	12443	67			*UGA*	0
tRNA-Leu2	+	12444	12505	62			*UAA*	0
ND1	+	12506	13442	937	*AUG*	*U--*		0
tRNA-Ile	+	13443	13506	64			*GAU*	0
tRNA-Lys	+	13507	13569	63			*UUU*	0
ND3	+	13570	13921	352	*AUG*	*U--*		0
tRNA-Ser2	+	13922	13985	64			*UCU*	0
ND2	+	13986	14988	1003	*AUG*	*U--*		0

Specimen 11：CJJ228 *Mesenchytraeus zhenggulen* 712

Gene	Strand	From	To	Size(pb)	Start	Stop	Anti-codon	Intergenic bases(bp)
COX1	+	1	1537	1537	*AUG*	*U--*		0
tRNA-Asn	+	1538	1604	67			*GUU*	0
COX2	+	1606	2284	679	*AUG*	*U--*		1
tRNA-Asp	+	2285	2345	62			*GUC*	0
ATP8	+	2348	2504	157	*AUG*	*U--*		2
tRNA-Tyr	+	2505	2566	62			*GUA*	0
tRNA-Gly	+	2567	2626	60			*UCC*	0
COX3	+	2628	3407	780	*AUG*	*UAA*		1
tRNA-Gln	+	3408	3476	69			*UUG*	0
ND6	+	3478	3954	477	*AUG*	*UAA*		1
CYTB	+	3947	5084	1138	*AUG*	*U--*		-8
tRNA-Trp	+	5085	5145	61			*UCA*	0
ATP6	+	5147	5842	696	*AUG*	*UAA*		1
Putative Control Region	+	5843	6387	545				0
tRNA-Arg	+	6388	6445	58			*UCG*	0
tRNA-His	+	6447	6506	60			*GUG*	1
ND5	+	6507	8241	1735	*AUG*	*U--*		0
tRNA-Phe	+	8242	8304	63			*GAA*	0
tRNA-Glu	+	8306	8368	63			*UUC*	1
tRNA-Pro	+	8369	8429	61			*UGG*	0
tRNA-Thr	+	8430	8491	62			*UGU*	0
ND4L	+	8492	8788	297	*AUG*	*UAA*		0
ND4	+	8782	10138	1357	*AUG*	*U--*		-7
tRNA-Cys	+	10139	10200	62			*GCA*	0
tRNA-Met	+	10201	10265	65			*CAU*	0
s-rRNA	+	10266	11030	765				0
tRNA-Val	+	11031	11092	62			*UAC*	0
l-rRNA	+	11093	12342	1250				0
tRNA-Leu1	+	12343	12404	62			*UAG*	0
tRNA-Ala	+	12405	12462	58			*UGC*	0
tRNA-Ser1	+	12463	12528	66			*UGA*	0
tRNA-Leu2	+	12529	12590	62			*UAA*	0
ND1	+	12591	13528	938	*AUG*	*UA-*		0
tRNA-Ile	+	13529	13591	63			*GAU*	0
tRNA-Lys	+	13592	13654	63			*UUU*	0
ND3	+	13655	14006	352	*AUG*	*U--*		0
tRNA-Ser2	+	14007	14071	64			*UCU*	0
ND2	+	14072	15077	1006	*AUG*	*U--*		0

Specimen 12：CJJ253 *Mesenchytraeus* sp. 704

Gene	Strand	From	To	Size(pb)	Start	Stop	Anti-codon	Intergenic bases(bp)
COX1	+	1	1537	1537	*AUG*	*U--*		0
tRNA-Asn	+	1538	1600	63			*GUU*	0
COX2	+	1602	2280	679	*AUG*	*U--*		1
tRNA-Asp	+	2281	2341	61			*GUC*	0
ATP8	+	2344	2500	157	*AUG*	*U--*		2
tRNA-Tyr	+	2501	2562	62			*GUA*	0
tRNA-Gly	+	2563	2622	60			*UCC*	0
COX3	+	2624	3403	780	*AUG*	*UAA*		1
tRNA-Gln	+	3406	3474	69			*UUG*	2
ND6	+	3476	3952	477	*AUG*	*UAA*		1
CYTB	+	3945	5082	1138	*AUG*	*U--*		-8
tRNA-Trp	+	5083	5144	62			*UCA*	0
ATP6	+	5146	5841	696	*AUG*	*UAA*		1
Putative Control Region	+	5842	6209	368				0
tRNA-Arg	+	6210	6269	60			*UCG*	0
tRNA-His	+	6271	6331	61			*GUG*	1
ND5	+	6332	8066	1735	*AUG*	*U--*		0
tRNA-Phe	+	8068	8128	61			*GAA*	1
tRNA-Glu	+	8130	8193	64			*UUC*	1
tRNA-Pro	+	8198	8261	64			*UGG*	4
tRNA-Thr	+	8262	8322	61			*UGU*	0
ND4L	+	8323	8619	297	*AUG*	*UAA*		0
ND4	+	8613	9969	1357	*AUG*	*U--*		-7
tRNA-Cys	+	9970	10031	62			*GCA*	0
tRNA-Met	+	10032	10094	63			*CAU*	0
s-rRNA	+	10095	10866	772				0
tRNA-Val	+	10867	10928	62			*UAC*	0
l-rRNA	+	10929	12173	1245				0
tRNA-Leu1	+	12174	12236	63			*UAG*	0
tRNA-Ala	+	12237	12295	59			*UGC*	0
tRNA-Ser1	+	12296	12362	67			*UGA*	0
tRNA-Leu2	+	12363	12426	64			*UAA*	0
ND1	+	12427	13364	938	*AUG*	*UA-*		0
tRNA-Ile	+	13365	13426	62			*GAU*	0
tRNA-Lys	+	13427	13489	63			*UUU*	0
ND3	+	13490	13842	353	*AUG*	*UA-*		0
tRNA-Ser2	+	13843	13906	64			*UCU*	0
ND2	+	13907	14909	1003	*AUG*	*U--*		0

Specimen 13：CJJ258 *Mesenchytraeus infradiverticulus* 689

Gene	Strand	From	To	Size(pb)	Start	Stop	Anti-codon	Intergenic bases(bp)
COX1	+	1	1537	1537	*AUG*	*U--*		0
tRNA-Asn	+	1538	1600	63			*GUU*	0
COX2	+	1602	2280	679	*AUG*	*U--*		1
tRNA-Asp	+	2281	2340	60			*GUC*	0
ATP8	+	2343	2499	157	*AUG*	*U--*		2
tRNA-Tyr	+	2500	2563	64			*GUA*	0
tRNA-Gly	+	2564	2623	60			*UCC*	0
COX3	+	2625	3404	780	*AUG*	*UAA*		1
tRNA-Gln	+	3408	3476	69			*UUG*	3
ND6	+	3478	3954	477	*AUG*	*UAA*		1
CYTB	+	3947	5084	1138	*AUG*	*U--*		-8
tRNA-Trp	+	5085	5145	61			*UCA*	0
ATP6	+	5147	5842	696	*AUG*	*UAA*		1
Putative Control Region	+	5843	6303	461				0
tRNA-Arg	+	6304	6364	61			*UCG*	0
tRNA-His	+	6366	6426	61			*GUG*	1
ND5	+	6427	8161	1735	*AUG*	*U--*		0
tRNA-Phe	+	8162	8223	62			*GAA*	0
tRNA-Glu	+	8225	8286	62			*UUC*	1
tRNA-Pro	+	8289	8351	63			*UGG*	2
tRNA-Thr	+	8352	8414	63			*UGU*	0
ND4L	+	8415	8711	297	*AUG*	*UAA*		0
ND4	+	8705	10061	1357	*AUG*	*U--*		-7
tRNA-Cys	+	10062	10124	63			*GCA*	0
tRNA-Met	+	10125	10186	62			*CAU*	0
s-rRNA	+	10187	10962	776				0
tRNA-Val	+	10963	11024	62			*UAC*	0
l-rRNA	+	11025	12277	1253				0
tRNA-Leu1	+	12279	12340	62			*UAG*	1
tRNA-Ala	+	12341	12399	59			*UGC*	0
tRNA-Ser1	+	12400	12466	67			*UGA*	0
tRNA-Leu2	+	12467	12528	62			*UAA*	0
ND1	+	12529	13467	939	*AUG*	*UAA*		0
tRNA-Ile	+	13468	13530	63			*GAU*	0
tRNA-Lys	+	13531	13593	63			*UUU*	0
ND3	+	13594	13945	352	*AUG*	*U--*		0
tRNA-Ser2	+	13946	14009	64			*UCU*	0
ND2	+	14010	15012	1003	*AUG*	*U--*		0

Specimen14：CJJ265 *Mesenchytraeus anisodiverticulus* 705

Gene	Strand	From	To	Size(pb)	Start	Stop	Anti-codon	Intergenic bases(bp)
COX1	+	1	1537	1537	*AUG*	*U--*		0
tRNA-Asn	+	1538	1601	64			*GUU*	0
COX2	+	1603	2281	679	*AUG*	*U--*		1
tRNA-Asp	+	2282	2345	64			*GUC*	0
ATP8	+	2348	2504	157	*AUG*	*U--*		2
tRNA-Tyr	+	2505	2577	73			*GUA*	0
tRNA-Gly	+	2578	2637	60			*UCC*	0
COX3	+	2639	3418	780	*AUG*	*UAA*		1
tRNA-Gln	+	3425	3493	69			*UUG*	6
ND6	+	3495	3971	477	*AUG*	*UAA*		1
CYTB	+	3964	5101	1138	*AUG*	*U--*		-8
tRNA-Trp	+	5102	5163	62			*UCA*	0
ATP6	+	5165	5866	702	*AUG*	*UAA*		1
Putative Control Region	+	5867	6098	232				0
tRNA-Arg	+	6099	6159	61			*UCG*	0
tRNA-His	+	6161	6222	62			*GUG*	1
ND5	+	6223	7957	1735	*AUG*	*U--*		0
tRNA-Phe	+	7958	8020	63			*GAA*	0
tRNA-Glu	+	8022	8082	61			*UUC*	1
tRNA-Pro	+	8088	8151	64			*UGG*	5
tRNA-Thr	+	8152	8213	62			*UGU*	0
ND4L	+	8214	8510	297	*AUG*	*UAA*		0
ND4	+	8504	9860	1357	*AUG*	*U--*		-7
tRNA-Cys	+	9861	9923	63			*GCA*	0
tRNA-Met	+	9929	9990	62			*CAU*	5
s-rRNA	+	9991	10778	788				0
tRNA-Val	+	10779	10840	62			*UAC*	0
l-rRNA	+	10841	12097	1257				0
tRNA-Leu1	+	12098	12160	63			*UAG*	0
tRNA-Ala	+	12161	12219	59			*UGC*	0
tRNA-Ser1	+	12220	12286	67			*UGA*	0
tRNA-Leu2	+	12288	12349	62			*UAA*	1
ND1	+	12350	13288	939	*AUG*	*UAA*		0
tRNA-Ile	+	13289	13351	63			*GAU*	0
tRNA-Lys	+	13352	13415	64			*UUU*	0
ND3	+	13416	13767	352	*AUG*	*U--*		0
tRNA-Ser2	+	13773	13836	64			*UCU*	5
ND2	+	13837	14842	1006	*AUG*	*U--*		0
