# Supplementary material for: De Novo Assembly and Comparative Analysis of the Complete Mitochondrial Genome of Mesenchytraeus (Annelida, Enchytraeidae)
Source: Ecol Evol. 2026 Sep 4;16(9):e74293. doi: 10.1002/ece3.74293 (PMC13543304; doi:10.1002/ece3.74293)
Supplement: Supplementary file 4 [file ECE3-16-e74293-s004.pdf]

Table S2b. Inverted Repeat Finder output for the DL sequence of *Mesenchytraeus* species

```
Sequence: gigachaetus-DL

    Indices: 299--323,441--465  Loop: 117  Score: 50
    Left Length: 25  Right Length: 25  Average length: 25.0
    3 most common centers (desc):  764  0  0

        289 >> (LF) AAAAAAGGGC
        475 << (RF) AAAATTACCA


        299 >> CGTGTAGCGGTGGTGCATTTTAGCA >> 323
        465 << ************************* << 441

Statistics
Matches: 25 (100.00%),  Mismatches: 0 (0.00%), Indels: 0 (0.00%)
ATpairs: 48.00%,  CGpairs: 52.00%,  GTpairs: 0.00%
```

```
Sequence: similigigachaetus-DL

    Indices: 265--291,424--450  Loop: 132  Score: 54
    Left Length: 27  Right Length: 27  Average length: 27.0
    3 most common centers (desc):  715  0  0

        255 >> (LF) AAAAAATTCT
        460 << (RF) ACAACTAACG


        265 >> ATGGGATTTGCAGTCGGGCCGATTTTG >> 291
        450 << *************************** << 424

Statistics
Matches: 27 (100.00%),  Mismatches: 0 (0.00%), Indels: 0 (0.00%)
ATpairs: 48.15%,  CGpairs: 51.85%,  GTpairs: 0.00%
```

```
Sequence: fokulen-DL

    Indices: 253--283,409--439  Loop: 125  Score: 53
    Left Length: 31  Right Length: 31  Average length: 31.0
    3 most common centers (desc):  692  0  0

        243 >> (LF) AAAAGCAAGG
        449 << (RF) ATTGTAAAAT


        253 >> ATGGGGGAATTGCGGCCGACGACAAATGCAG >> 283
        439 << ************************C****** << 409

Statistics
Matches: 30 (96.77%),  Mismatches: 1 (3.23%), Indels: 0 (0.00%)
ATpairs: 40.00%,  CGpairs: 60.00%,  GTpairs: 0.00%
```

```
Sequence: Mesenchytraeus_sp-DL
    Indices: 200--247,290--338  Loop: 42  Score: 64
    Left Length: 48  Right Length: 49  Average length: 48.5
    3 most common centers (desc):  538  537  0

        190 >> (LF) GGAAAAAAAT
        348 << (RF) GTAAAATTAC


        200 >> GCGTGTTAGATTGTGGGCGTTTCGCACTGAA-ATTTTGACCAT-ATAATT >>
247
        338 << *******************************G*G**-******G****** <<
290

Statistics
Matches: 46 (92.00%),  Mismatches: 1 (2.00%), Indels: 3 (6.00%)
ATpairs: 58.70%,  CGpairs: 41.30%,  GTpairs: 0.00%
```
