# Supplementary material for: De Novo Assembly and Comparative Analysis of the Complete Mitochondrial Genome of Mesenchytraeus (Annelida, Enchytraeidae)
Source: Ecol Evol. 2026 Sep 4;16(9):e74293. doi: 10.1002/ece3.74293 (PMC13543304; doi:10.1002/ece3.74293)
Supplement: Supplementary file 5 [file ECE3-16-e74293-s005.pdf]

Supplementary Figure S2. *H*-strand secondary structures of the long unassigned regions putatively corresponding to the control regions of the different species of *Mesenchytraeus*. Shown plots correspond to the minimum energy structures predicted by mfold (Zuker, 2003) .

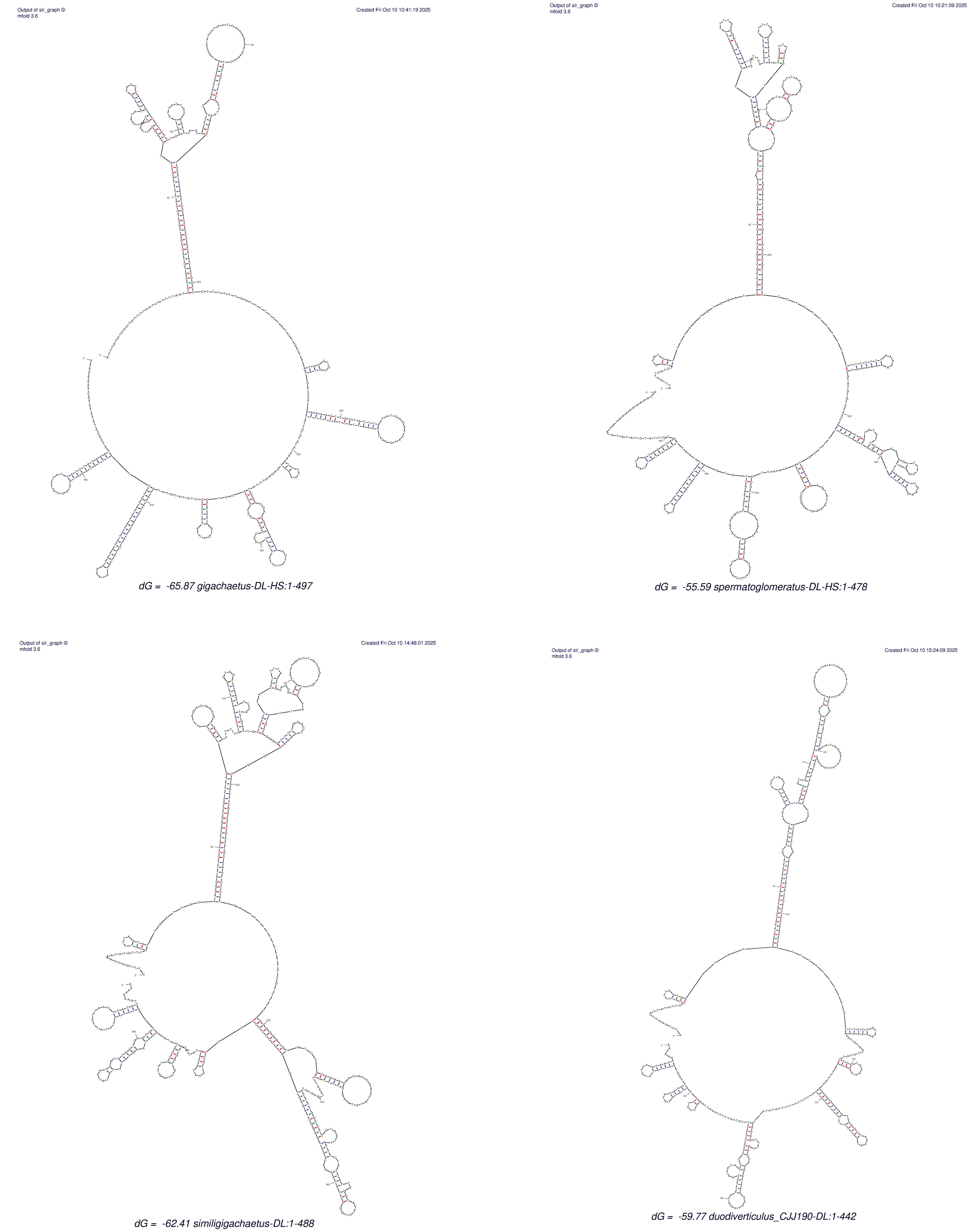

Output of sir_graph ©
mfold 3.6

Created Fri Oct 10 15:42:11 2025

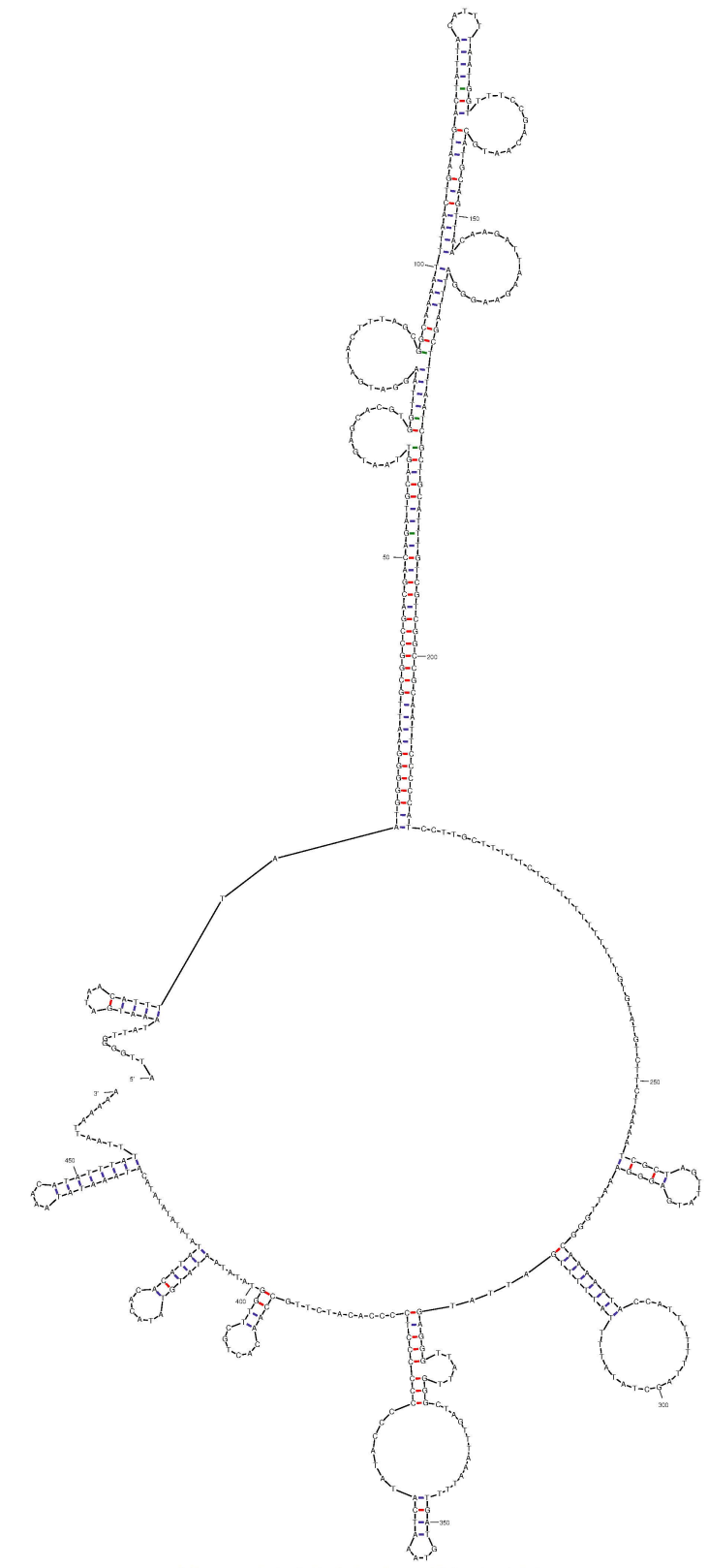

dG = -61.92 fokulen-DL:1-466

Output of sir_graph ©
mfold 3.6

Created Fri Oct 10 15:45:08 2025

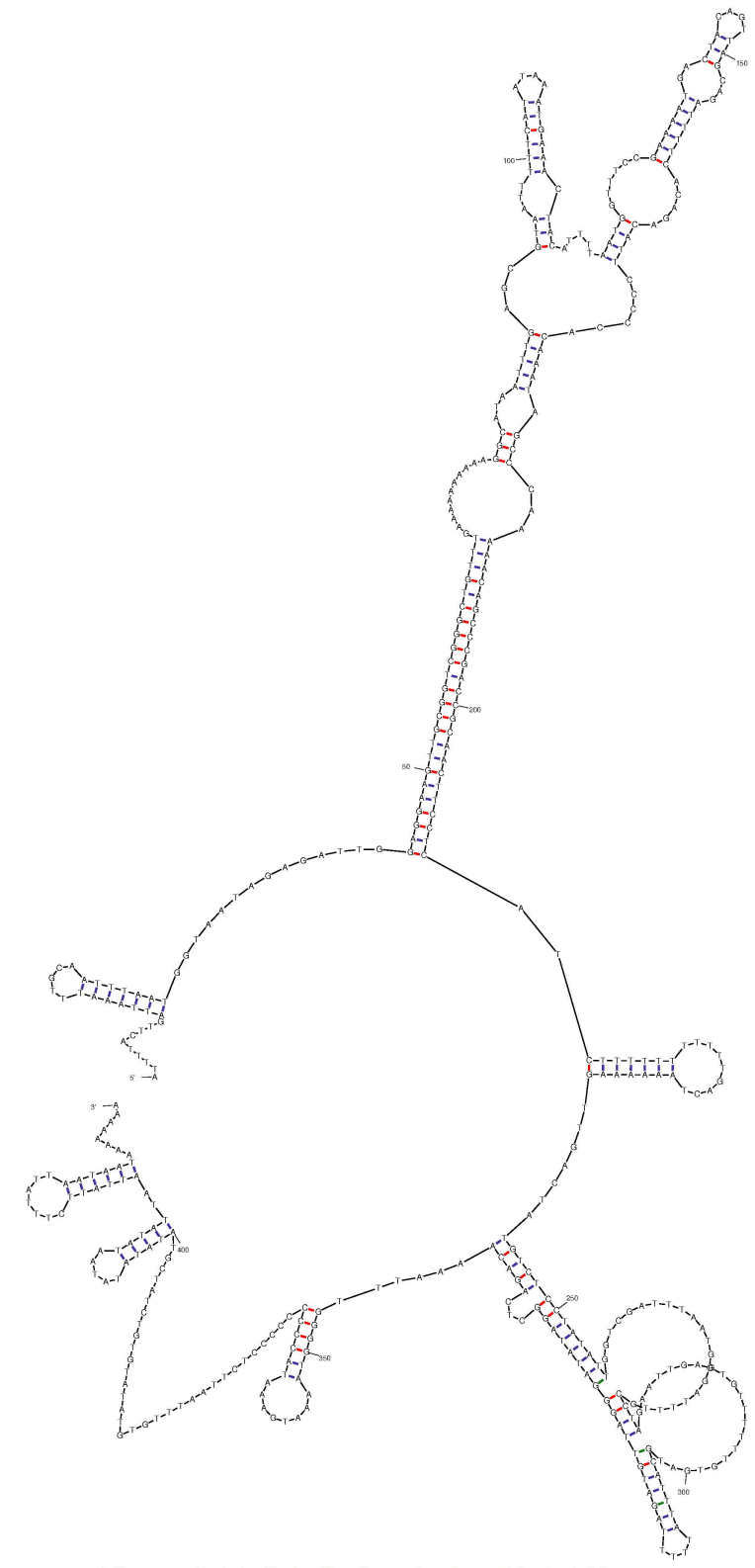

dG = -52.14 digitalisdiverticulus-DL:1-443

Output of sir_graph ©
mfold 3.6

Created Fri Oct 10 15:47:01 2025

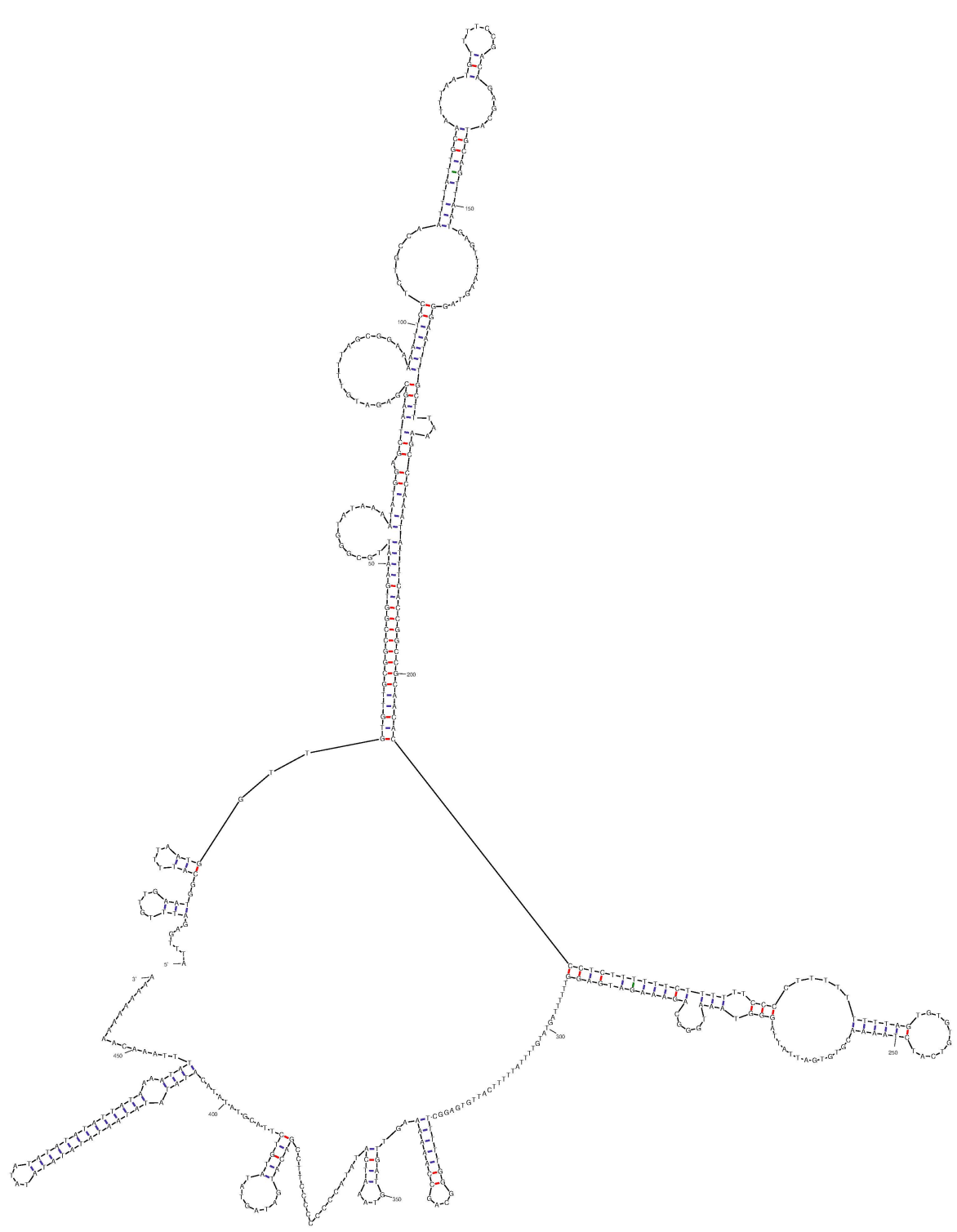

dG = -51.35 ngulen-DL:1-461

Output of sir_graph ©
mfold 3.6

Created Fri Oct 10 15:49:09 2025

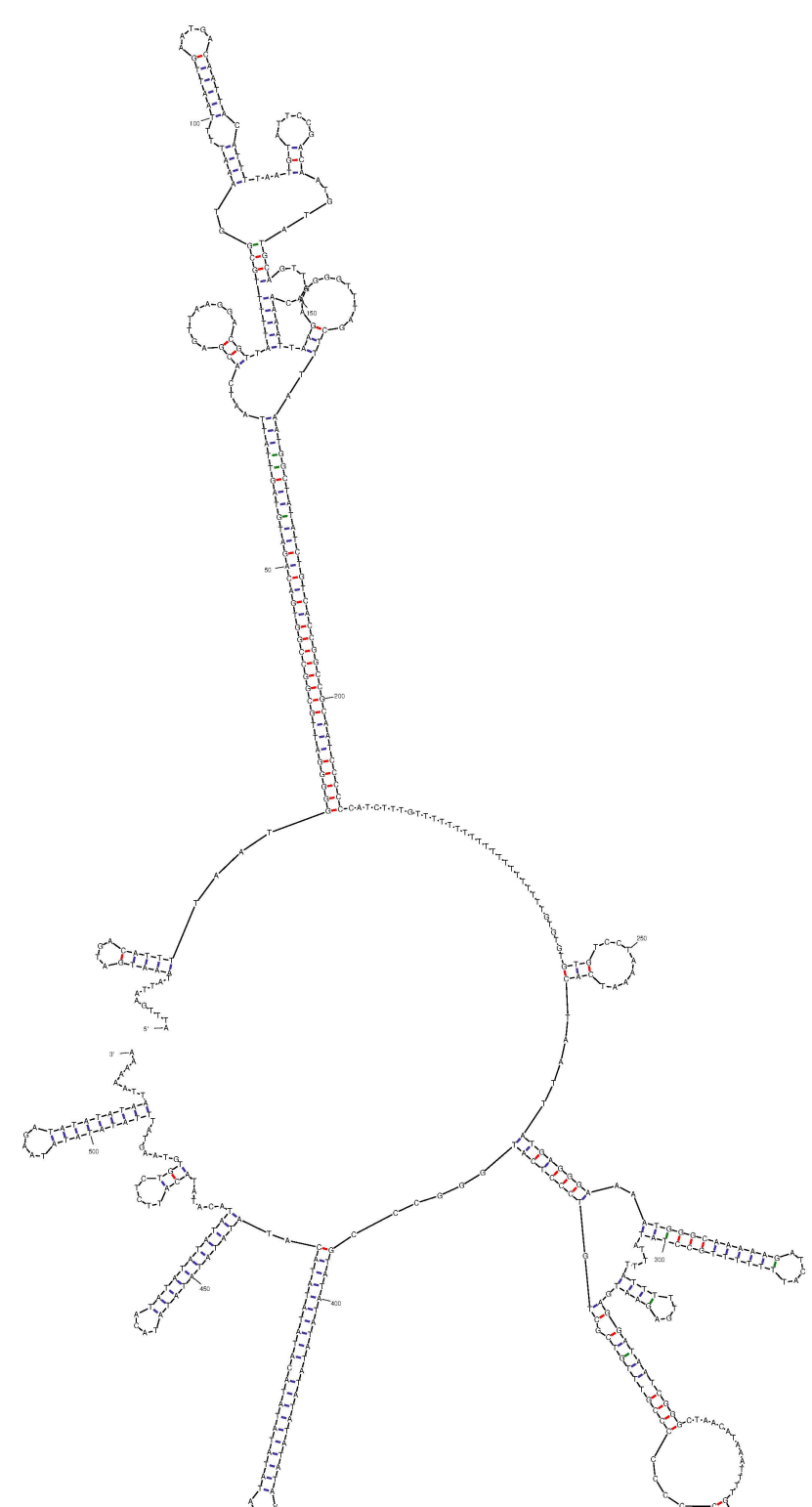

dG = -77.55 monodiverticulus-DL:1-525

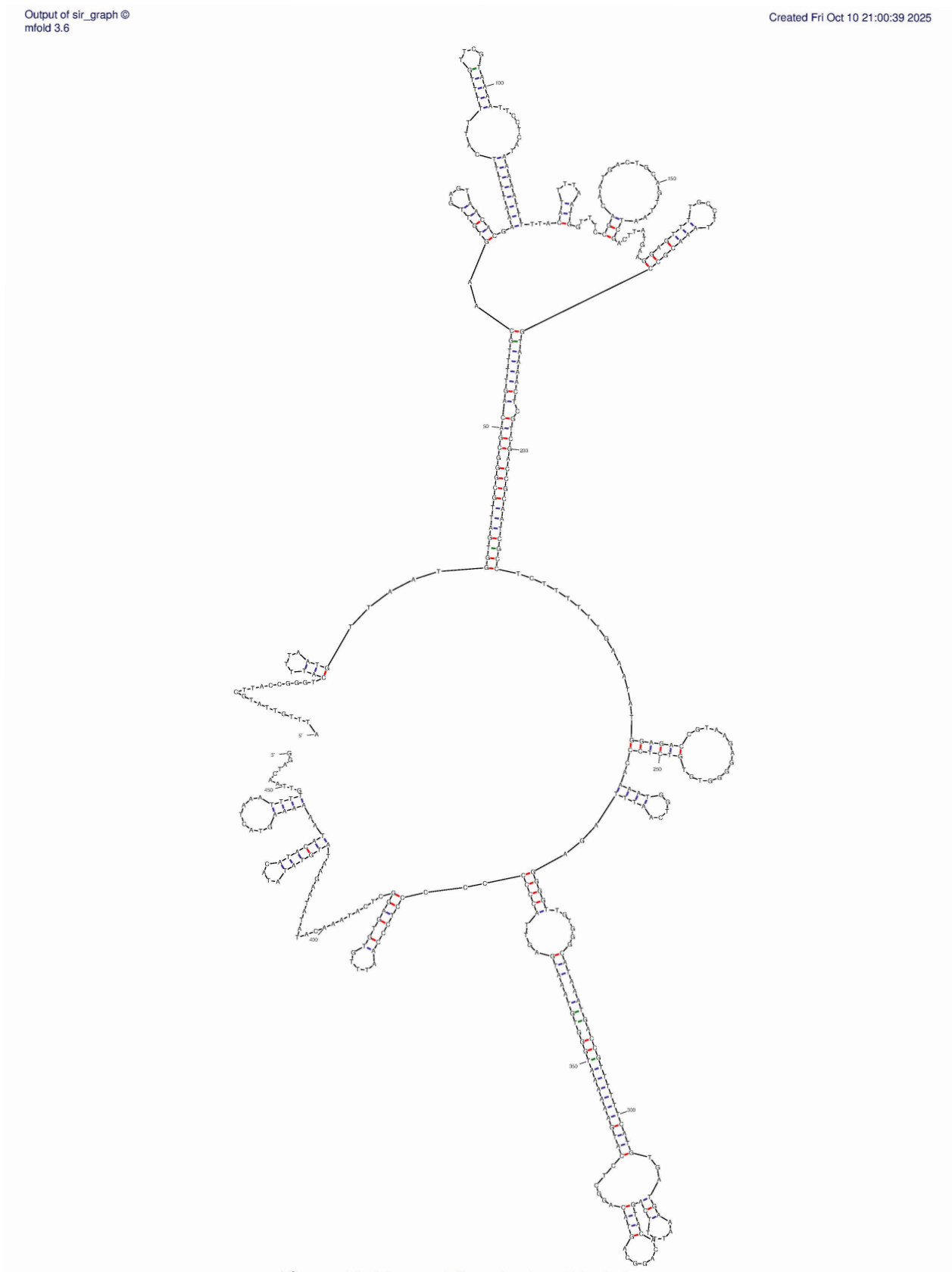

dG = -48.36 parvidiverticulus-DL:1-457

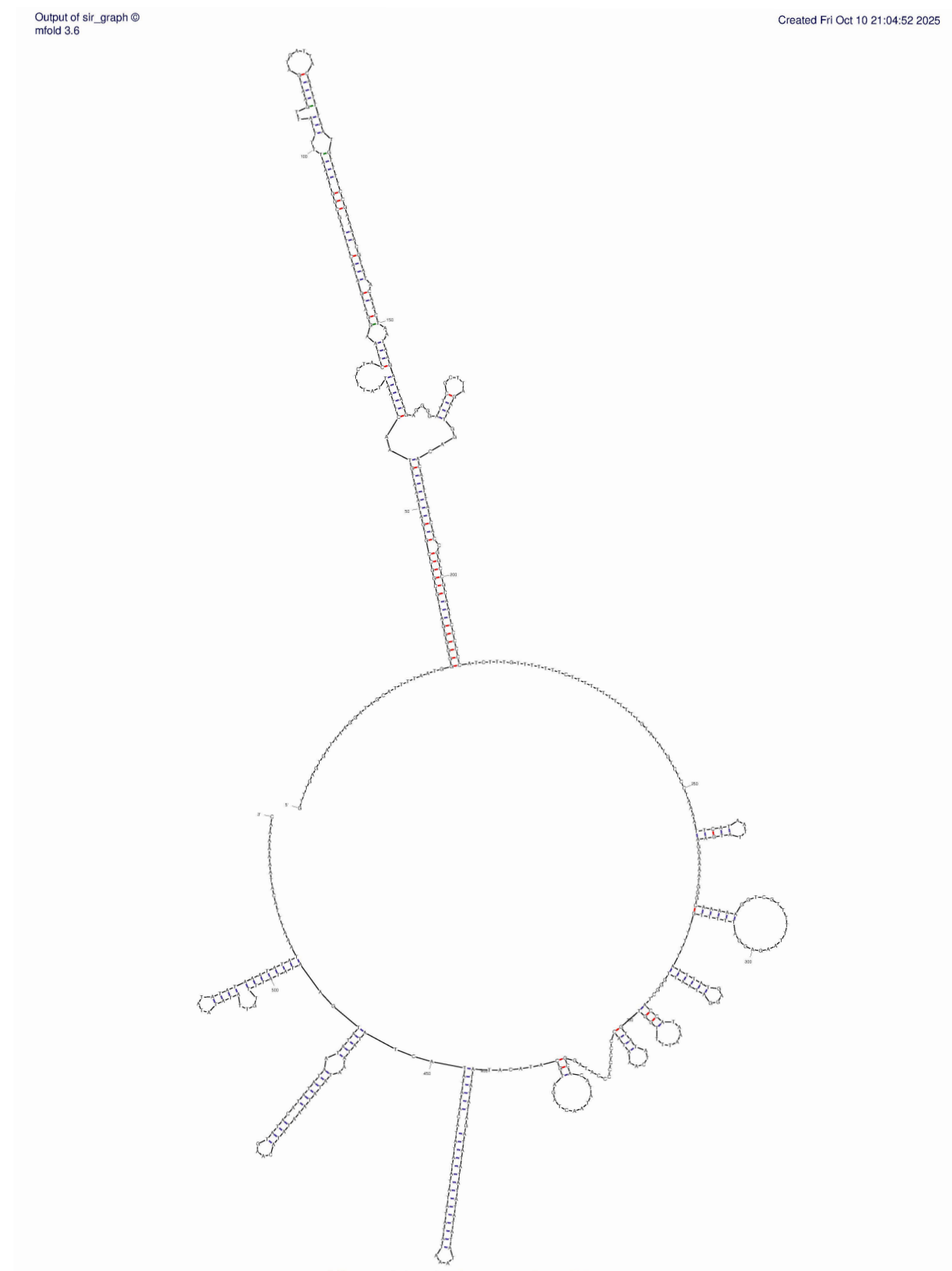

dG = -61.51 zhenggulen-DL:1-545

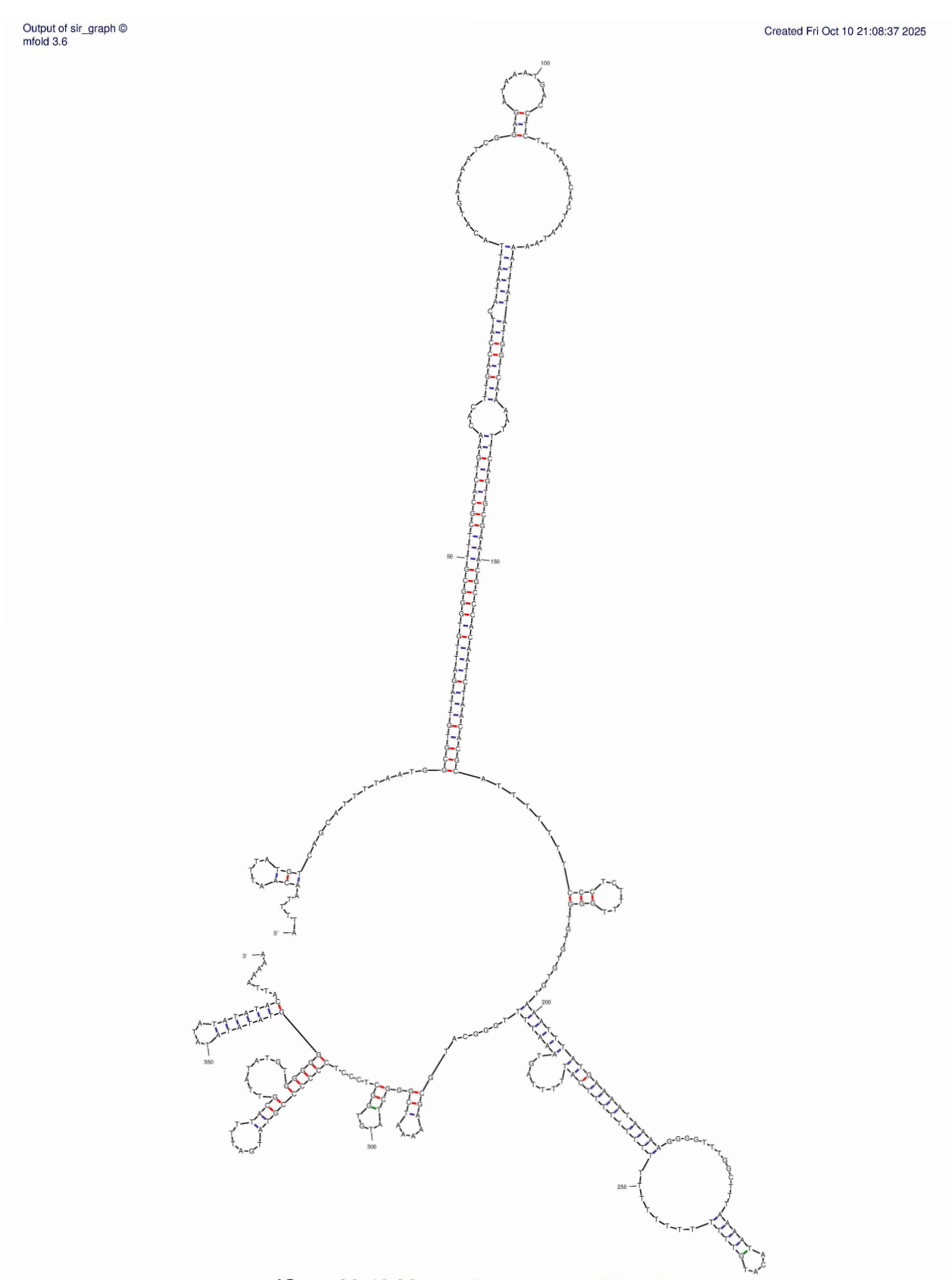

dG = -62.49 Mesenchytraeus_sp-DL:1-368

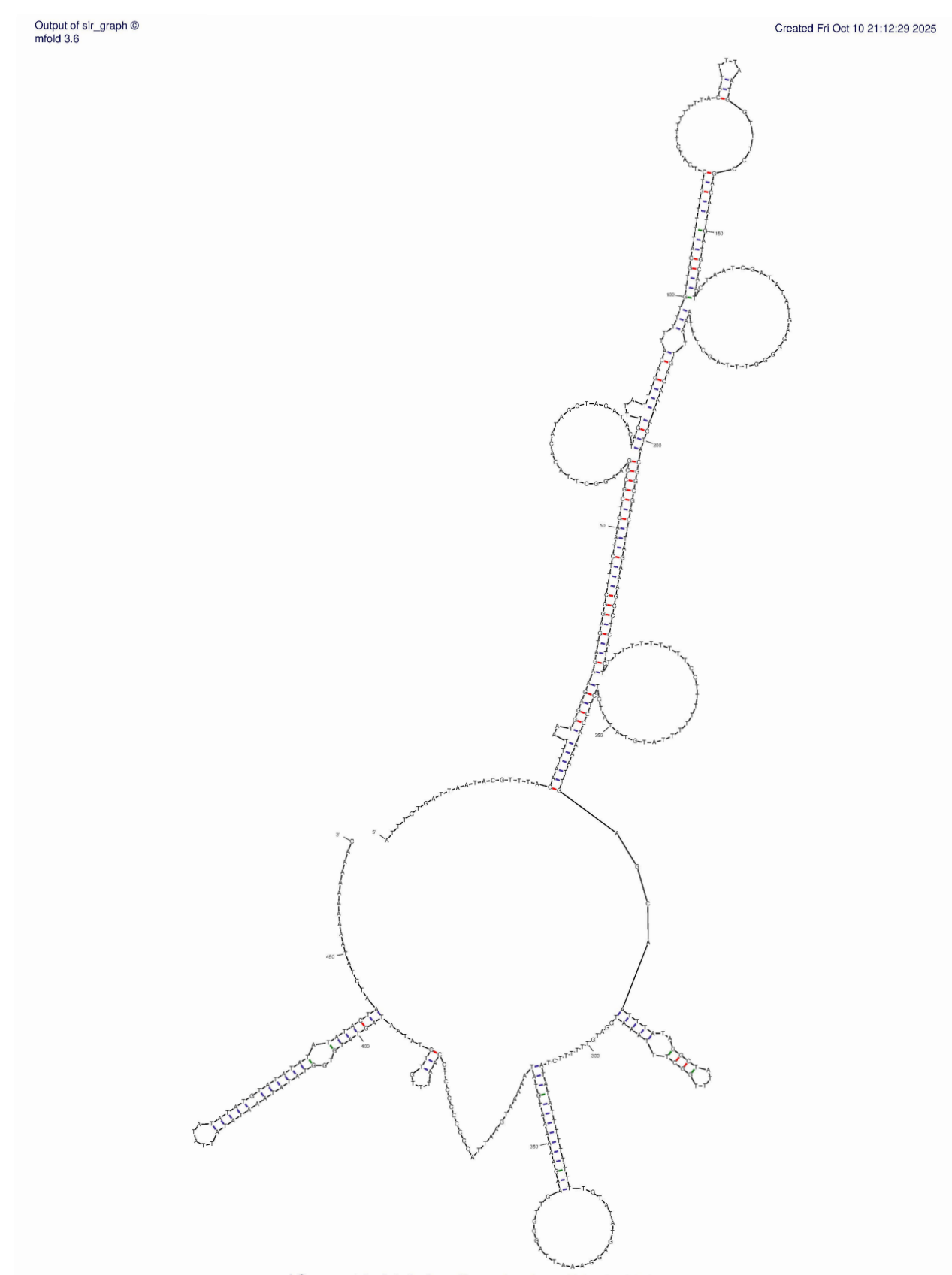

dG = -48.00 infradiverticulus-DL:1-461

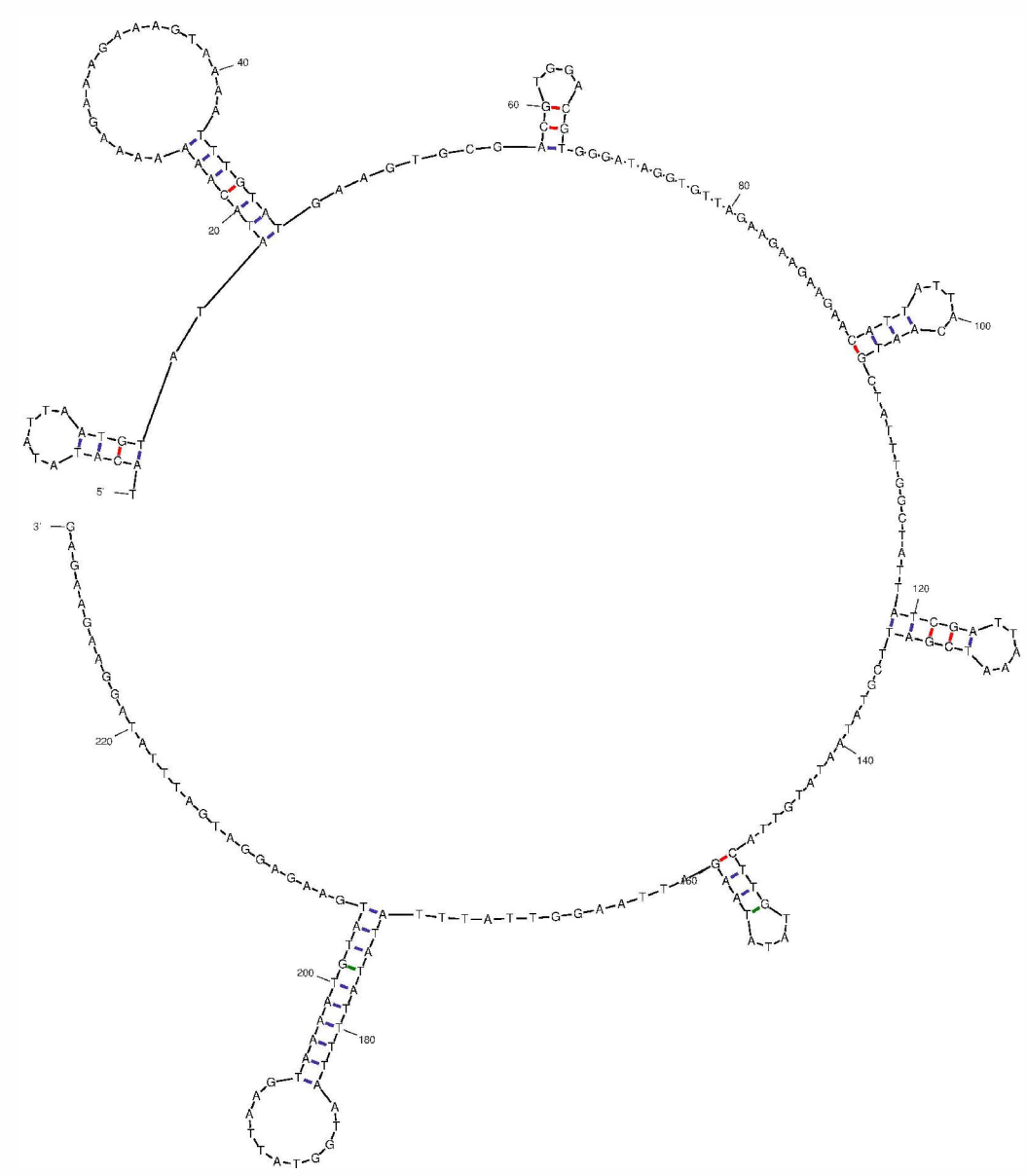

dG = -16.51 anisodiverticulus-DL:1-231
